# Three-component *N*-alkenylation of azoles with alkynes and iodine(III) electrophile: synthesis of multisubstituted *N*-vinylazoles

**DOI:** 10.3762/bjoc.20.79

**Published:** 2024-04-22

**Authors:** Jun Kikuchi, Roi Nakajima, Naohiko Yoshikai

**Affiliations:** 1 Graduate School of Pharmaceutical Sciences, Tohoku University, 6-3 Aoba, Aramaki, Aoba-ku, Sendai 980-8578, Japan

**Keywords:** alkynes, azoles, cross-coupling, hypervalent iodine

## Abstract

A stereoselective *N*-alkenylation of azoles with alkynes and iodine(III) electrophile is reported. The reaction between various azoles and internal alkynes is mediated by benziodoxole triflate as the electrophile in a *trans*-fashion, affording azole-bearing vinylbenziodoxoles in moderate to good yields. The tolerable azole nuclei include pyrazole, indazole, 1,2,3-triazole, benzotriazole, and tetrazole. The iodanyl group in the product can be leveraged as a versatile synthetic handle, allowing for the preparation of hitherto inaccessible types of densely functionalized *N*-vinylazoles.

## Introduction

**Scheme 1:**
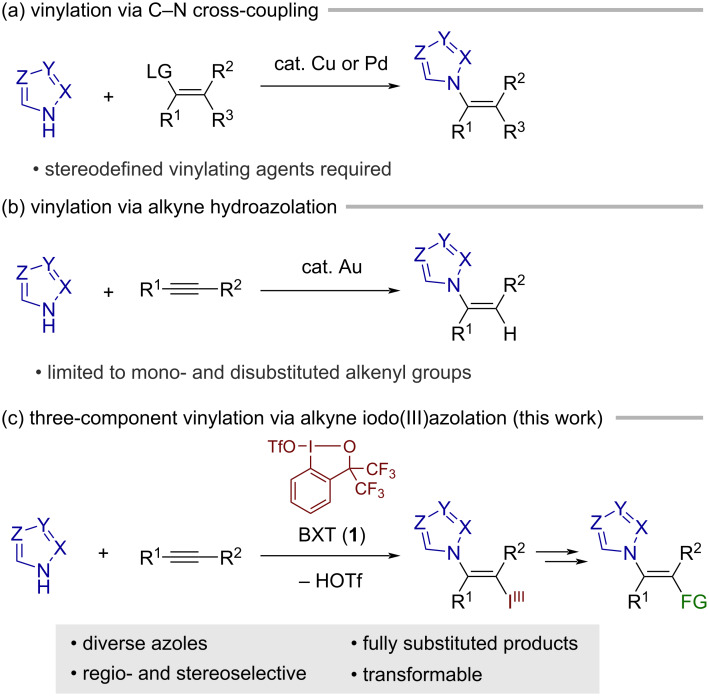
Synthesis of *N*-vinylazoles.

*N*-Functionalized azoles are prevalent in bioactive natural products and pharmaceutical agents, including antifungal drugs [1–3], and hence their selective preparation has attracted considerable attention from the synthetic community. Compared to methods for the de novo construction of azole heterocycles, direct functionalization of the azole N–H bond offers the unique merit of enabling rapid access to structurally diverse *N*-functionalized azoles because one versatile method would potentially apply to various azole nuclei. In this context, the *N*-alkenylation of azoles represents an attractive transformation due to the occurrence of the *N*-vinylazole motif in bioactive compounds and the synthetic utility of its olefinic C=C bond. The most extensively explored approach to this transformation is the transition metal-catalyzed C–N coupling between azoles and vinylating agents, including vinyl halides [4], boronates [5], sulfonium salts [6–8], and iodonium salts [9], which usually occurs with the retention of the stereochemistry of the vinylating agents (Scheme 1a). Nonetheless, this approach is not necessarily suited for the stereoselective preparation of densely substituted *N*-vinylazoles because preparing the requisite multisubstituted vinylating agents, preferably with well-defined stereochemistry, is a nontrivial task.

The addition of azoles to alkynes represents an alternative approach to *N*-vinylazoles. For example, Nolan and co-workers recently reported a gold-catalyzed addition of azoles to alkynes (hydroazolation; Scheme 1b) [10]. The gold catalysis encompassed various azoles such as pyrazole, indazole, and (benzo)triazole, exhibiting high *Z*-selectivity. In addition, Cao et al. reported a gold-catalyzed addition of 5-substituted tetrazoles to terminal alkynes [11]. Analogous hydroazolation reactions of alkynes have also been achieved under other metal-catalyzed conditions [12–13] or base-mediated conditions [14], with varying scopes of azoles and alkynes. Despite such advances, the hydroazolation approach is intrinsically limited to the preparation of mono- or disubstituted vinylazoles. Herein, we report on a three-component *N*-vinylation reaction of azoles with alkynes and iodine(III) electrophile, benziodoxole triflate (BXT, **1**; Scheme 1c). Displaying exclusive *trans*-selectivity, the reaction tolerates a broad range of azoles, including pyrazole, 1,2,3-triazole, tetrazole, indazole, and benzotriazole, with internal alkynes as coupling partners. The resulting products represent a new class of functionalized vinylbenziodoxoles (VBXs) [15–21], which have recently emerged as unique vinylating agents [22–25]. Thus, the follow-up transformation of the iodanyl group in the present products allows for the preparation of hitherto inaccessible types of densely functionalized vinylazoles with tetrasubstituted olefinic moiety.

## Results and Discussion

Our group has demonstrated benziodoxole triflate (BXT) [26] and related compounds as a versatile iodine(III) electrophile for the inter- and intramolecular difunctionalization of alkynes with various heteroatom and carbon nucleophiles [27–34]. Specifically, intermolecular *trans*-iodo(III)functionalization of alkynes has been achieved using oxygen nucleophiles such as alcohols [28,32], ethers [33], carboxylic acids [31], phosphate esters [31], and sulfonic acids [31]. On the other hand, nitrogen-based nucleophiles amenable to this reaction manifold have thus far been limited to nitriles in the context of Ritter-type iodo(III)amidation [29]. In light of the significance of vinylated azoles, our attention was attracted to the feasibility of iodo(III)azolation using various azoles (a single example of iodo(III)azolation using pyrazole was reported in [28]).

Table 1 summarizes the results of the optimization of the reaction conditions for the vinylation of pyrazole (**2a**) with 1-phenyl-1-propyne (**3a**) and BXT (**1**). Upon examination of various reaction parameters, the desired three-component *N*-alkenylation was found to proceed smoothly by reacting **3a** with excess amounts of **1** (2 equiv) and **2a** (5 equiv) in MeCN (0.2 M) at room temperature, affording the *trans*-difunctionalized product **4aa** as a single regio- and stereoisomer in 77% yield (Table 1, entry 1). Decreasing or increasing the concentration did not improve the yield of **4aa** (Table 1, entries 2 and 3). The reaction became rather sluggish in different solvents such as HFIP and Et_2_O (Table 1, entries 4 and 5). By reducing the equivalents of **2a** to 3 equiv and 2 equiv, the yield of **4aa** dropped to 66% and 55%, respectively (Table 1, entries 6 and 7). The addition of a base such as K_2_CO_3_ completely shut down the desired reaction (Table 1, entry 8). It is worth noting that the replacement of **1** with *N*-iodosuccinimide, a common iodine(I) electrophile, failed to promote an analogous iodoazolation reaction, highlighting the unique utility of the iodine(III) electrophile in the present alkyne difunctionalization.

**Table 1:** Optimization of reaction conditions.^a^

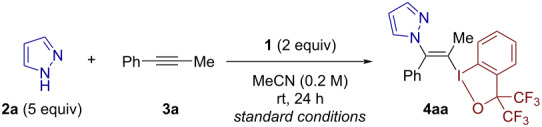		

Entry	Deviation from standard conditions	Yield (%)^b^

1	none	77^c^
2	*c* = 0.1 M	50
3	*c* = 0.4 M	70
4	HFIP as the solvent (0.2 M)	16
5	Et_2_O as the solvent (0.2 M)	22
6	3 equiv of **2a**	66
7	2 equiv of **2a**	55
8	2 equiv of **2a**, 2 equiv of K_2_CO_3_	trace

^a^The reaction was performed on a 0.1 mmol scale; ^b^Determined by ^1^H NMR using 1,1,2,2-tetrachloroethane as an internal standard; ^c^Isolated yield.

**Scheme 2:**
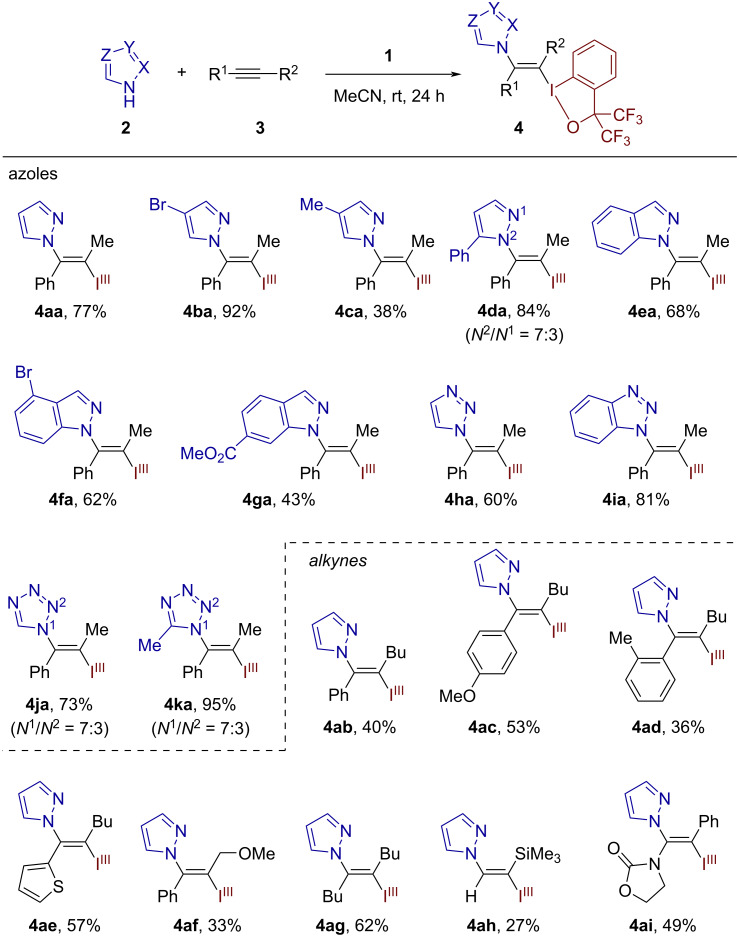
Scope of three-component *N*-alkenylation of azoles.

With the standard conditions (Table 1, entry 1) in hand, we explored the scope of the three-component *N*-vinylation (Scheme 2). First, various azoles were subjected to the vinylation reaction using alkyne **3a** and BXT (**1**). 4-Bromo and 4-methylpyrazoles afforded the desired products **4ba** and **4ca** in 92% and 38% yields, respectively. 3-Phenylpyrazole underwent competitive alkenylation at the N1 and N2 positions, affording the N2-alkenylated product **4da** and its N1-regioisomer in 84% overall yield in a ratio of 7:3. Indazole and its 4-bromo and 6-methoxycarbonyl analogues afforded the expected N1-alkenylated products **4ea**–**4ga** in 43–68% yields. 1,2,3-Triazole and benzotriazole both smoothly participated in the reaction to give their respective products **4ha** and **4ia** in 60% and 81% yields, respectively. Tetrazole and 5-methyltetrazole both proved to be excellent substrates. Regardless of the presence or absence of the 5-substituent, they underwent preferential alkenylation at the N1 position over the N2 position with the identical regioselectivity of 7:3 (see **4ja** and **4ka**). Among other five-membered aromatic azacycles, imidazole completely failed to undergo the present *N*-alkenylation, whereas the reaction of 1,2,4-triazole was too sluggish to allow for the isolation and unambiguous characterization of the expected product.

Next, the reaction of pyrazole (**2a**) was explored using different alkynes. A series of (hetero)aryl(alkyl)alkynes were successfully engaged as reaction partners to give the products **4ab**–**4af** in moderate yields, displaying tolerance to *o*-tolyl (**4ad**), 2-thienyl (**4ae**), and methoxymethyl (**4af**) groups. A dialkylalkyne such as 5-decyne smoothly underwent the difunctionalization reaction to afford the product **4ag** in 62% yield. While sluggish, trimethylsilylacetylene was selectively azolated at the terminal position (see **4ah**), which could be rationalized by the better stabilization of a partial positive charge at this position by the β-silyl substituent. Finally, an oxazolidinone-substituted ynamide also proved to undergo iodo(III)azolation in a regio- and stereoselective fashion to give the product **4ai** in a moderate yield. Note that terminal alkynes such as phenylacetylene also took part in the reaction, albeit in a much-diminished yield (7% by ^1^H NMR; data not shown). Observing no byproducts originating from phenylacetylene, we speculate that the lack of reactivity stems from the relatively low electron density of the terminal alkyne, which likely leads to direct coordination of pyrazole to the iodine(III) reagent.

**Scheme 3:**
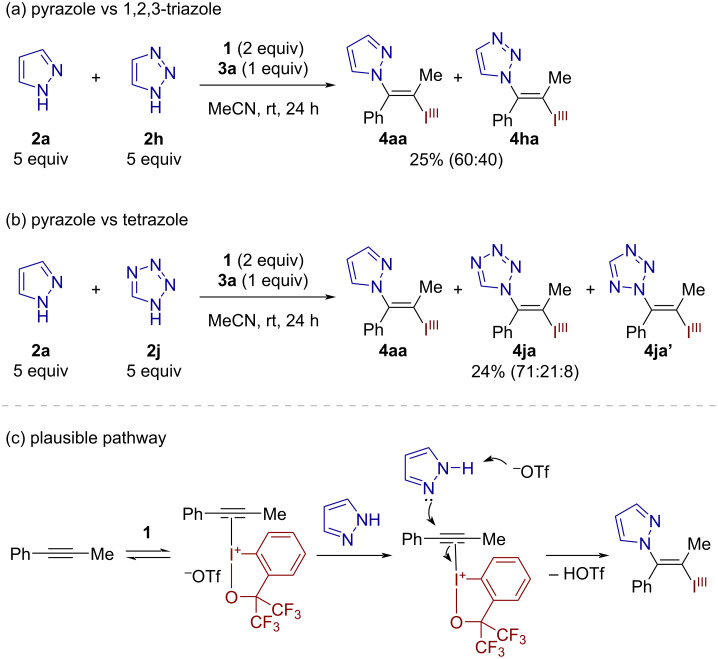
Competition experiments and plausible reaction pathway.

To probe the relative reactivity of different azoles, competition experiments were performed. The reaction of excess (5 equiv each) pyrazole (**2a**) and 1,2,3-triazole (**2h**) with the alkyne **3a** and **1** afforded a mixture of the corresponding products **4aa** and **4ha** in a ratio of 60:40 (Scheme 3a). Performed in the same manner, the competition between **2a** and tetrazole (**2j**) resulted in **4aa** as the major adduct, accompanied by the tetrazole adducts **4ja** and **4ja’** (the ratio **4aa**/**4ja**/**4ja’** = 71:21:8; Scheme 3b). Superficially, these results appear correlated with the acidity of the corresponding azoles (p*K*_a_ value: pyrazole, 19.8; 1,2,3-triazole, 13.9; tetrazole, 8.2), with the lowest-acidic pyrazole being the most competitive. However, we rather surmise that the Lewis basicity of the proton-free nitrogen atom of the azole would have more direct relevance to the results of the competition experiments, where the least Lewis basic tetrazole was the least competitive. Along with this conjecture, the present reaction is proposed to involve reversible complexation between the alkyne and the cationic iodine(III) electrophile and subsequent *trans*-addition of the azole nucleophile, the latter step being coupled with concomitant deprotonation of the N–H bond by the triflate anion (Scheme 3c). It is important to note that the azole nucleophile preferentially adds to the carbon atom that can better stabilize a positive charge, as demonstrated by the regioselectivities observed with unsymmetrical alkynes. The failure of imidazole to participate in the iodo(III)azolation may be attributed to its much greater Lewis basicity compared to other azoles, likely killing the reactivity of the iodine(III) electrophile by direct coordination.

**Scheme 4:**
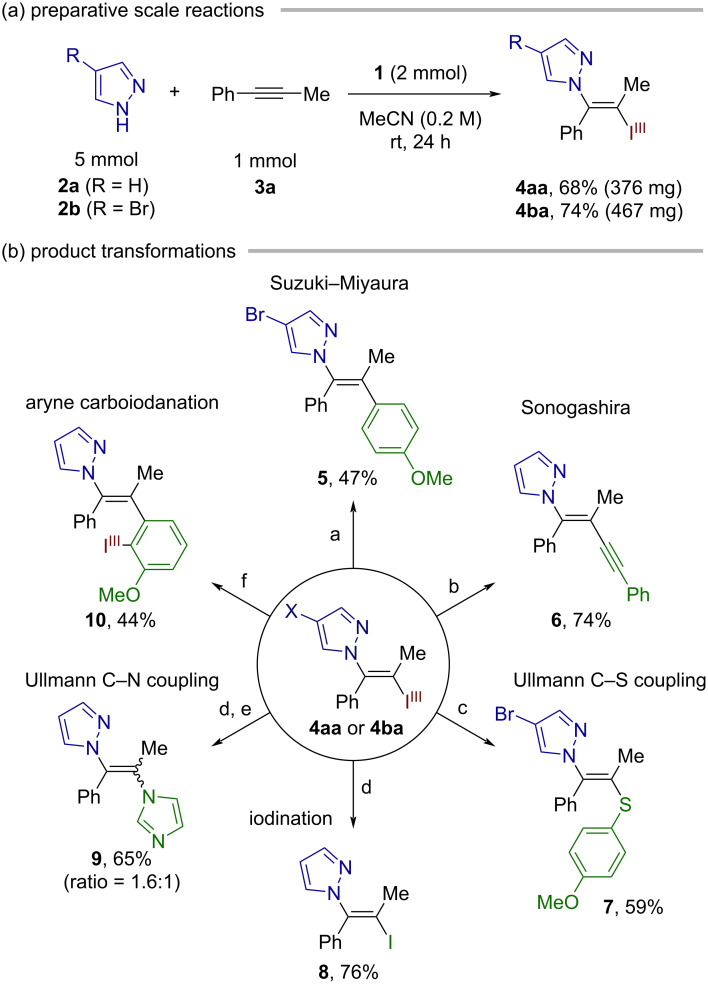
Preparative-scale reaction and product transformations. Reaction conditions: (a) Pd(PPh_3_)_4_, 4-MeOC_6_H_4_B(OH)_2_, Cs_2_CO_3_, DMF/H_2_O, 60 °C, 18 h. (b) Pd(OAc)_2_, PPh_3_, CuI, phenylacetylene, Et_3_N, 50 °C, 5 h. (c) CuI, neocuproine, 4-MeOC_6_H_4_SH, NaO*t-*Bu, toluene, 110 °C, 13 h. (d) CuI, ʟ-proline, DMF, 80 °C, 14 h. (e) CuI, imidazole, Cs_2_CO_3_, DMF, 120 °C, 15 h. (f) 3-Methoxy-2-(trimethylsilyl)phenyl triflate, CsF, MeCN, rt, 18 h.

The present reaction could be performed on a preparative scale. Thus, 1 mmol-scale synthesis of the vinylazoles **4aa** and **4ba** could be successfully performed in 68% and 74% yields, respectively (Scheme 4a). Furthermore, the iodanyl group on these products serves as a versatile handle for downstream transformations, thus allowing for the stereoselective preparation of various trisubstituted *N*-vinylazoles (Scheme 4b). Pd-catalyzed C–C couplings such as Suzuki–Miyaura and Sonogashira couplings on **4aa** or **4ba** afforded the desired products **5** and **6** in 47% and 74% yields, respectively. In the former case, the C–Br bond on the pyrazole moiety remained intact, highlighting the superior leaving group ability of the BX group. Cu-catalyzed Ullmann coupling between **4ba** and 4-methoxybenzenethiol furnished the N,S-substituted olefin **7** in 59% yield. The treatment of **4aa** with stoichiometric CuI and ʟ-proline effected the iodine(III)-to-iodine(I) conversion to give the vinyl iodide **8** in 76% yield. Compound **8** was used for the Ullmann coupling with imidazole, producing the vicinal *N*-heterocycle-substituted olefin **9** as a mixture of stereoisomers in 65% yield. Finally, **4aa** proved to be a viable nucleophilic VBX for the carboiodanation of 3-methoxybenzyne [35], furnishing the new *ortho*-alkenylated arylbenziodoxole **10** with exclusive C–C bond formation at the distal aryne carbon [36].

## Conclusion

In summary, we have reported a three-component *N*-vinylation reaction of azoles with alkynes and iodine(III) electrophile. The present reaction represents a rare example of the installation of stereodefined trisubstituted alkenyl groups into the azole core, encompassing various azole nucleophiles including pyrazole, indazole, 1,2,3-triazole, benzotriazole, and tetrazole. The follow-up transformation of the iodanyl group provides a means to prepare hitherto inaccessible types of alkenylated azoles. Further exploration of the three-component alkenylation of nitrogen and other heteroatom nucleophiles is currently underway.

## Supporting Information

File 1 Experimental procedures and characterization data of new compounds.

## Data Availability

All data that supports the findings of this study is available in the published article and/or the supporting information to this article.
