# Supplementary material for: Three-component N-alkenylation of azoles with alkynes and iodine(III) electrophile: synthesis of multisubstituted N-vinylazoles
Source: Beilstein J Org Chem. 2024 Apr 22;20:891–7. doi: 10.3762/bjoc.20.79 (PMC11070964; doi:10.3762/bjoc.20.79)

BEILSTEIN JOURNAL OF ORGANIC CHEMISTRY

Supporting Information

for

Three-component *N*-alkenylation of azoles with alkynes and iodine(III) electrophile: synthesis of multisubstituted *N*-vinylazoles

Jun Kikuchi, Roi Nakajima and Naohiko Yoshikai

Beilstein J. Org. Chem. **2024,** *20,* 891–897. doi:10.3762/bjoc.20.79

Experimental procedures and characterization data of new compounds

License and Terms: This is a supporting information file under the terms of the Creative Commons Attribution License (https://creativecommons.org/licenses/by/4.0). Please note that the reuse, redistribution and reproduction in particular requires that the author(s) and source are credited and that individual graphics may be subject to special legal provisions.
The license is subject to the *Beilstein Journal of Organic Chemistry* terms and conditions: (https://www.beilstein-journals.org/bjoc/terms)

Contents

1. Materials and methods S2

2. General procedures and product characterization..S4

3. Product transformations S24

4. References..S29

5. NMR Spectra S30

1. Materials and methods

General. All reactions dealing with air- or moisture-sensitive compounds were performed by standard Schlenk technique in oven-dried reaction vessels under an argon atmosphere. Analytical thin-layer chromatography (TLC) was performed on Merck 60 F254 silica gel plates. Flash chromatography was performed using 40–50 μm silica gel (silica gel 60N, Kanto Chemical). ^{1}H, ^{13}C{^{1}H}, and ^{19}F{^{1}H} NMR spectra were recorded on JEOL JNM-ECA600 (600 MHz) spectrometers. ^{1}H and ^{13}C{^{1}H} NMR spectra are reported in parts per million (ppm) downfield from an internal standard, tetramethylsilane (0 ppm) and $CHCl_3$ (77.0 ppm), respectively. ^{19}F{^{1}H} NMR spectra are referenced to external standard (CF_3CO_2H, –76.6 ppm). Structural assignments were made with additional information from the NOESY experiment. Melting points were determined with a MPA100 OptiMelt apparatus. High-resolution mass spectra (HRMS) were recorded on a JEOL JMS-700 spectrometer equipped with a double-focusing mass analyzer or a JMS-T100GC spectrometer equipped with a TOF mass analyzer.

Materials. Unless otherwise noted, commercial reagents were purchased from TCI, Kanto Chemical, Sigma-Aldrich, or other commercial suppliers and used as received. Anhydrous MeCN was purchased from FUJIFILM Wako Pure Chemical and was used as received. DMF and toluene were distilled over CaH_2 and stored under argon in the presence of molecular sieves 4 Å. 3,3-Bis(trifluoromethyl)-1λ^3-benzo[*d*][1,2]iodaoxol-1(3*H*)-yl trifluoromethanesulfonate (benziodoxole triflate, BXT, **1**)[1] and alkynes **3c**–**f**[2] and **3i**[3] were prepared according to the literature procedures and spectral data for these compounds showed good agreement with the literature data.

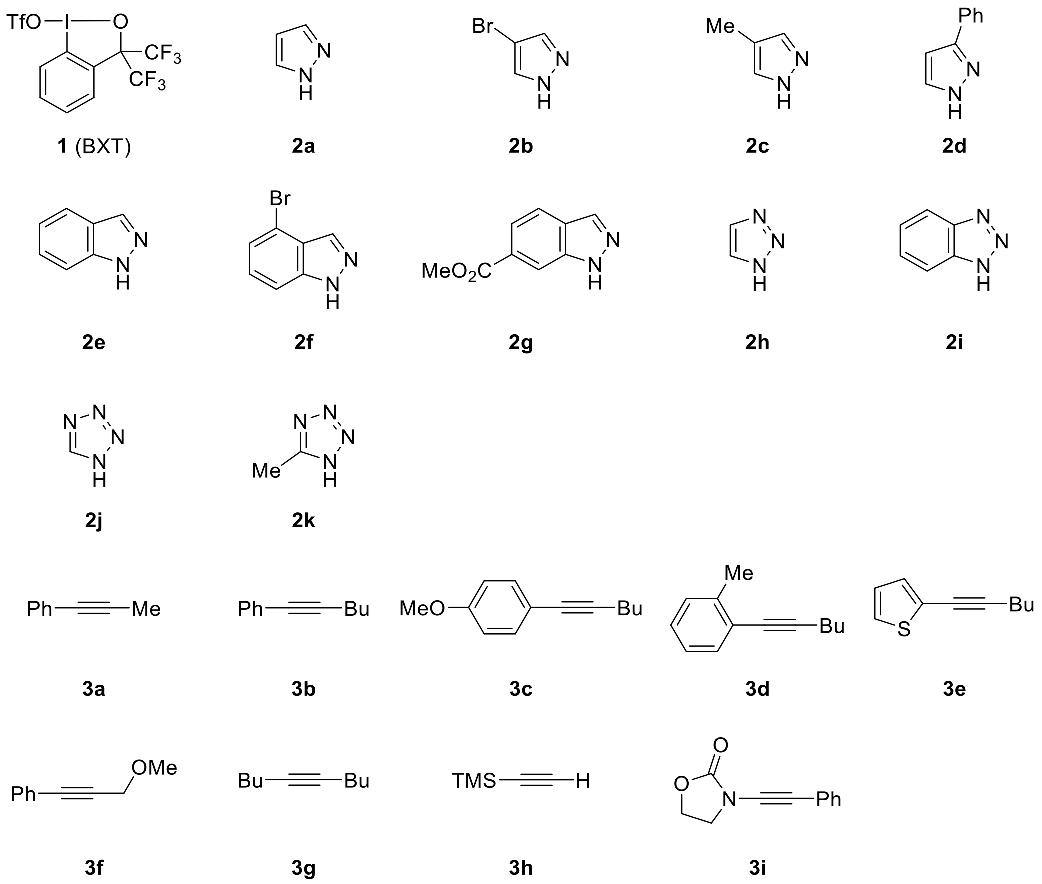

Figure S1. Starting materials used in this study.

2. General procedures and product characterization

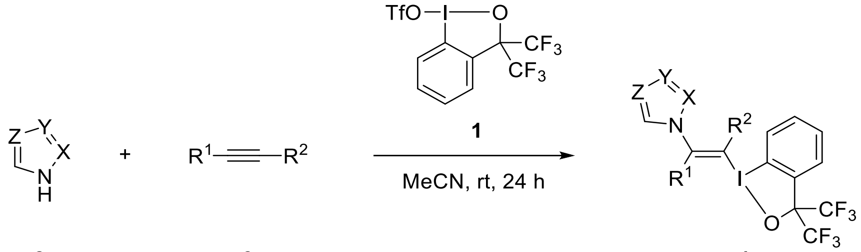

2 **3** **4**

General procedure: Under an argon atmosphere, a 4 mL vial equipped with a magnetic stir bar was charged sequentially with azole **2** (0.50 mmol, 5.0 equiv), alkyne **3** (0.10 mmol), and MeCN (0.5 mL), followed by the addition of **1** (103.6 mg, 0.20 mmol, 2.0 equiv). The resulting mixture was stirred at room temperature for 24 h. Saturated Na_2CO_3 aq. (4 mL) was added, and then the mixture was extracted with EtOAc (5 mL × 3). The combined organic layer was washed with H_2O (5 mL) and brine (5 mL), dried over Na_2SO_4, and concentrated under reduced pressure. The residue was purified by flash chromatography on silica gel to afford the desired product.

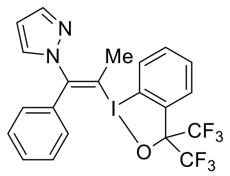

(*E*)-1-(2-(3,3-Bis(trifluoromethyl)-1λ^3-benzo[*d*][1,2]iodaoxol-1(3*H*)-yl)-1-phenylprop-1-en-1-yl)-1*H*-pyrazole (4aa): Synthesized by the general procedure (42.5 mg, 77%); yellow solid; R_f 0.18 (hexane/EtOAc = 1/1); m.p. 108.1-110.3 °C; ^{1}H NMR (600 MHz, $CDCl_3$) δ 7.86 (d, J = 7.2 Hz, 1H), 7.75 (d, J = 1.2 Hz, 1H), 7.69-7.64 (m, 3H), 7.40 (tt, J = 7.2 Hz, 1.2 Hz, 1H), 7.33-7.29 (m, 2H), 7.26-7.25 (m, 1H), 7.15-7.12 (m, 2H), 6.38 (dd, J = 2.4 Hz, 1.8 Hz, 1H), 2.83 (s, 3H); ^{13}C{^{1}H} NMR (151 MHz, $CDCl_3$) δ 148.0, 141.1, 137.5, 132.4, 131.7, 131.6, 130.6, 130.54, 130.49, 128.94, 12.89, 127.3, 123.8 (q, $J_{C\text{-}F}$ = 291.7 Hz), 116.5, 110.7, 107.1, 81.4-80.6 (m), 24.8; ^{19}F{^{1}H} NMR (565 MHz, $CDCl_3$) δ -76.0; HRMS (FAB) *m/z*: [M + H]$^+$ calcd for $C_{21}H_{16}F_6IN_2O$ 553.0206; found, 553.0233.

1 mmol-scale synthesis of 4aa: Under an argon atmosphere, a 20 mL two-necked flask equipped with a magnetic stir bar was charged sequentially with **2a** (340.4 mg, 5.0 mmol, 5.0 equiv), **3a** (116.2 mg, 1.0 mmol), and MeCN (5.0 mL), followed by the addition of **1** (1.04 g, 2.0 mmol, 2.0 equiv). The resulting mixture was stirred at room temperature for 24 h. Saturated Na_2CO_3 aq. (5 mL) was added, and then the mixture was extracted with EtOAc (15 mL × 3). The combined organic layer was washed with H_2O (15 mL) and brine (15 mL), dried over Na_2SO_4, and concentrated under reduced pressure. The residue was purified by flash chromatography on silica gel to afford **4aa** (375.6 mg, 68%).

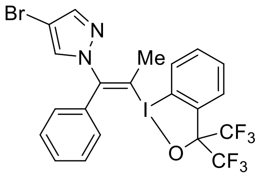

(*E*)-1-(2-(3,3-Bis(trifluoromethyl)-1λ³-benzo[*d*][1,2]iodaoxol-1(3*H*)-yl)-1-phenylprop-1-en-1-yl)-4-bromo-1*H*-pyrazole (4ba): Synthesized by the general procedure (58.1 mg, 92%); white solid; R_f 0.33 (hexane/EtOAc = 2/1); m.p. 40.4-42.1 °C; ^{1}H NMR (600 MHz, $CDCl_3$) δ 7.86 (d, J = 6.0 Hz, 1H), 7.70 (s, 1H), 7.68-7.63 (m, 3H), 7.42-7.38 (m, 1H), 7.33-7.29 (m, 2H), 7.27 (s, 1H), 7.13 (d, J = 7.8 Hz, 2H), 2.81 (s, 3H); ^{13}C{^{1}H} NMR (151 MHz, $CDCl_3$) δ147.2, 141.7, 136.9, 132.5, 131.6, 131.5, 130.8, 130.67, 130.63, 129.1, 128.9, 127.3, 123.8 (q, $J_{C\text{-}F}$ = 291.7 Hz), 117.7, 110.5, 95.6, 81.2-80.8 (m), 24.7; ^{19}F{^{1}H} NMR (565 MHz, $CDCl_3$) δ -75.9; HRMS (FAB) *m*/*z*: $[M + H]^+$ calcd for $C_{21}H_{15}BrF_6IN_2O$ 630.9311; found, 630.9306.

1 mmol-scale synthesis of 4ba: Under an argon atmosphere, a 20 mL two-necked flask equipped with a magnetic stir bar was charged sequentially with **2b** (734.9 mg, 5.0 mmol, 5.0 equiv), **3a** (116.2 mg, 1.0 mmol), and MeCN (5.0 mL), followed by the addition of **1** (1.04 g, 2.0 mmol, 2.0 equiv). The resulting mixture was stirred at room temperature for 24 h. Saturated Na_2CO_3 aq. (5 mL) was added, and then the mixture was extracted with EtOAc (15 mL × 3).

The combined organic layer was washed with H_2O (15 mL) and brine (15 mL), dried over Na_2SO_4, and concentrated under reduced pressure. The residue was purified by flash chromatography on silica gel to afford **4ba** (467.1 mg, 74%).

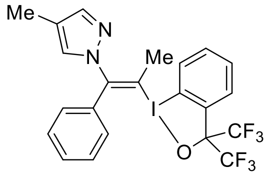

(*E*)-1-(2-(3,3-Bis(trifluoromethyl)-1λ^3-benzo[*d*][1,2]iodaoxol-1(3*H*)-yl)-1-phenylprop-1-en-1-yl)-4-methyl-1*H*-pyrazole (4ca): Synthesized by the general procedure (21.5 mg, 38%); white solid; R_f 0.21 (hexane/EtOAc = 1/1); m.p. 112.5-114.5 °C; ^{1}H NMR (600 MHz, $CDCl_3$) δ 7.85 (d, J = 6.6 Hz, 1H), 7.70-7.62 (m, 3H), 7.55 (s, 1H), 7.39 (t, J = 7.8 Hz, 1H), 7.31 (t, J = 7.8 Hz, 2H), 7.13 (d, J = 7.8 Hz, 2H), 6.99 (s, 1H), 2.84 (s, 3H), 2.07 (s, 3H); ^{13}C{^{1}H} NMR (151 MHz, $CDCl_3$) δ 148.1, 142.2, 137.7, 132.4, 131.6, 130.6, 130.52, 130.50, 130.2, 129.0, 128.9, 127.3, 123.9 (q, J = 294.1 Hz), 117.8, 115.2, 110.6, 81.4-81.0 (m), 24.8, 8.8; ^{19}F{^{1}H} NMR (565 MHz, $CDCl_3$) δ -76.0; HRMS (FAB) *m*/*z*: $[M + H]^+$ calcd for $C_{22}H_{18}F_6IN_2O$ 567.0363; found, 567.0376.

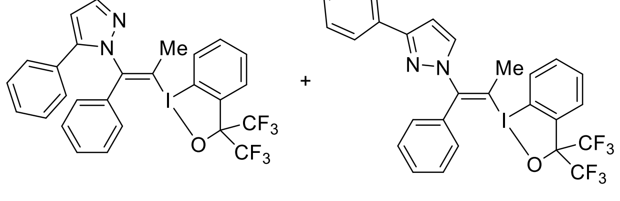

4da **4da'**

(*E*)-1-(2-(3,3-Bis(trifluoromethyl)-1λ^3-benzo[*d*][1,2]iodaoxol-1(3*H*)-yl)-1-phenylprop-1-en-1-yl)-5-phenyl-1*H*-pyrazole (4da) and (*E*)-1-(2-(3,3-Bis(trifluoromethyl)-1λ^3-benzo[*d*][1,2]iodaoxol-1(3*H*)-yl)-1-phenylprop-1-en-1-yl)-3-phenyl-1*H*-pyrazole (4da'): Synthesized by the general procedure and obtained as regioisomeric mixtures (52.8 mg, 84%, ratio = 7/3 as determined by ^{1}H NMR), which were separated by column chromatography on

silica gel; the structural assignments for the regioisomers were made by NOESY analysis (see below); **4da**: white solid; R_f 0.37 (hexane/EtOAc = 1/1); m.p. 170.0-172.4 °C; ^{1}H NMR (600 MHz, $CDCl_3$) δ 7.85 (d, J = 7.2 Hz, 1H), 7.79 (d, J = 1.8 Hz, 1H), 7.65 (t, J = 7.2 Hz, 1H), 7.55 (t, J = 6.6 Hz, 1H), 7.41-7.35 (m, 2H), 7.33 (t, J = 7.5 Hz, 2H), 7.26-7.21 (m, 3H), 7.13 (t, J = 7.5 Hz, 2H), 7.01-6.97 (m, 2H), 6.48 (d, J = 1.8 Hz, 1H), 2.40 (s, 3H); ^{13}C{^{1}H} NMR (151 MHz, $CDCl_3$) δ 148.6, 145.1, 141.2, 137.1, 132.6, 131.5, 130.6, 130.1, 130.0, 128.9, 128.6 128.5, 128.3, 128.2, 127.4, 123.8 (q, $J_{C\text{-}F}$ = 292.0 Hz), 123.0, 110.7, 106.9, 81.2-80.8 (m), 24.3; ^{19}F{^{1}H} NMR (565 MHz, $CDCl_3$) δ -76.0; HRMS (FAB) *m*/*z*: $[M + H]^+$ calcd for $C_{27}H_{20}F_6IN_2O$ 629.0519; found, 629.0535.; **4da'**: white solid; R_f 0.45 (hexane/EtOAc = 1/1); m.p. 181.9-184.2 °C; ^{1}H NMR (600 MHz, $CDCl_3$) δ 7.90-7.88 (m, 2H), 7.88-7.85 (m, 1H), 7.74-7.72 (m, 1H), 7.70-7.64 (m, 2H), 7.46 (t, J = 7.8 Hz, 2H), 7.42 (tt, J = 7.8 Hz, 1.2 Hz, 1H), 7.38 (tt, J = 7.8 Hz, 1.8 Hz, 1H), 7.33 (t, J= 7.8 Hz, 2H), 7.21-7.19 (m, 2H), 7.19 (d, J = 1.8 Hz, 1H), 6.67 (d, J = 2.4 Hz, 1H), 3.00 (s, 3H); ^{13}C{^{1}H} NMR (151 MHz, $CDCl_3$) δ 153.1, 147.5, 137.5, 133.3, 132.4, 131.7, 130.6, 130.5, 129.2, 129.0, 128.8, 128.6, 127.3, 125.9, 123.9 (q, $J_{C\text{-}F}$ = 295.7 Hz), 115.7, 110.7, 104.6, 81.3-80.9 (m), 25.2; ^{19}F{^{1}H} NMR (565 MHz, $CDCl_3$) δ -76.0; HRMS (FAB) *m*/*z*: $[M + H]^+$ calcd for $C_{27}H_{20}F_6IN_2O$ 629.0519; found, 629.0535.

NOESY spectra of **4da**

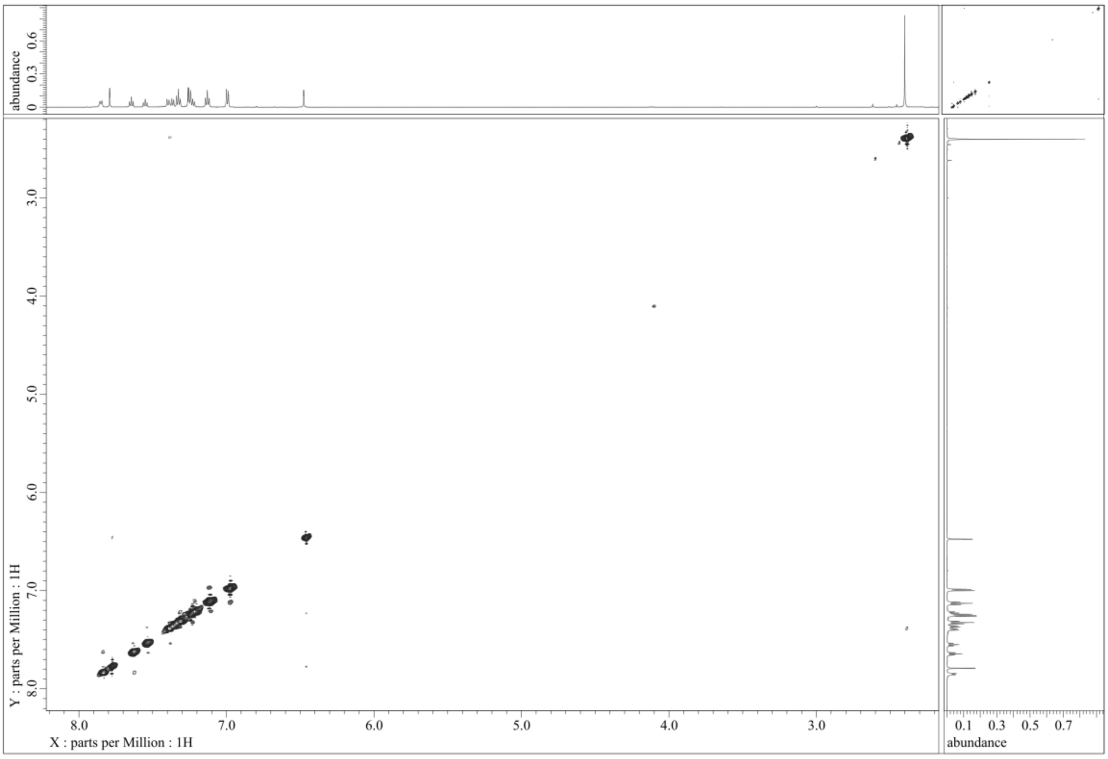

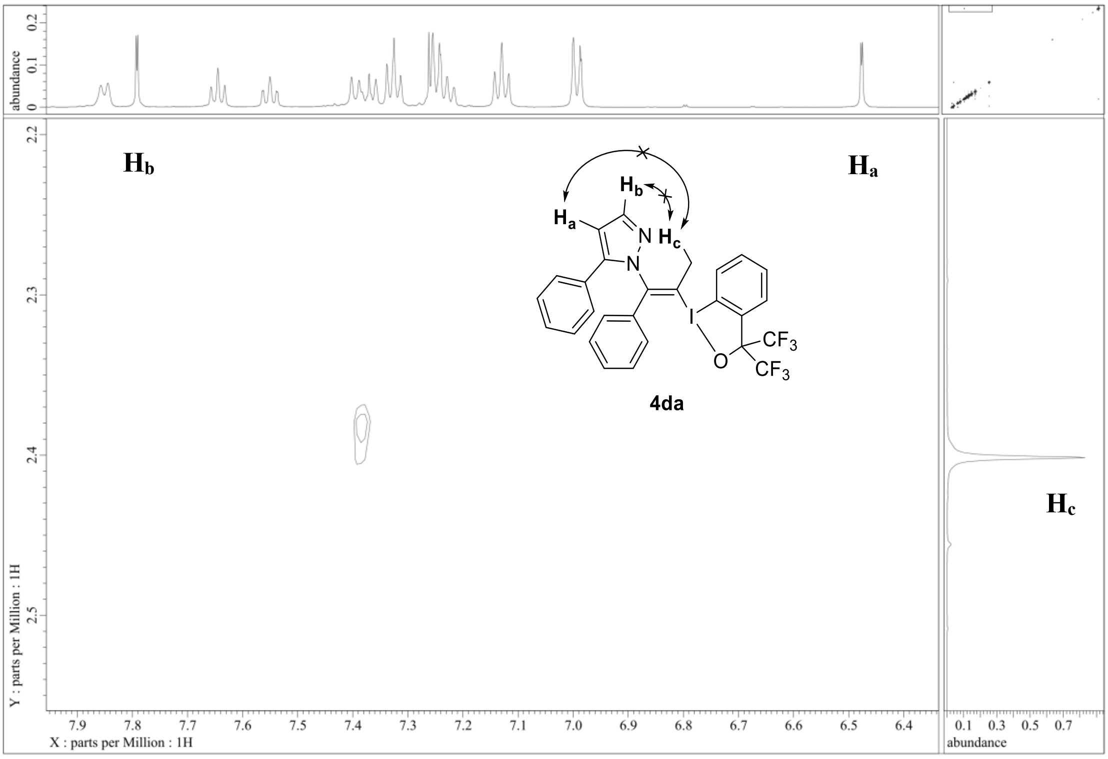

NOESY spectra of **4da'**

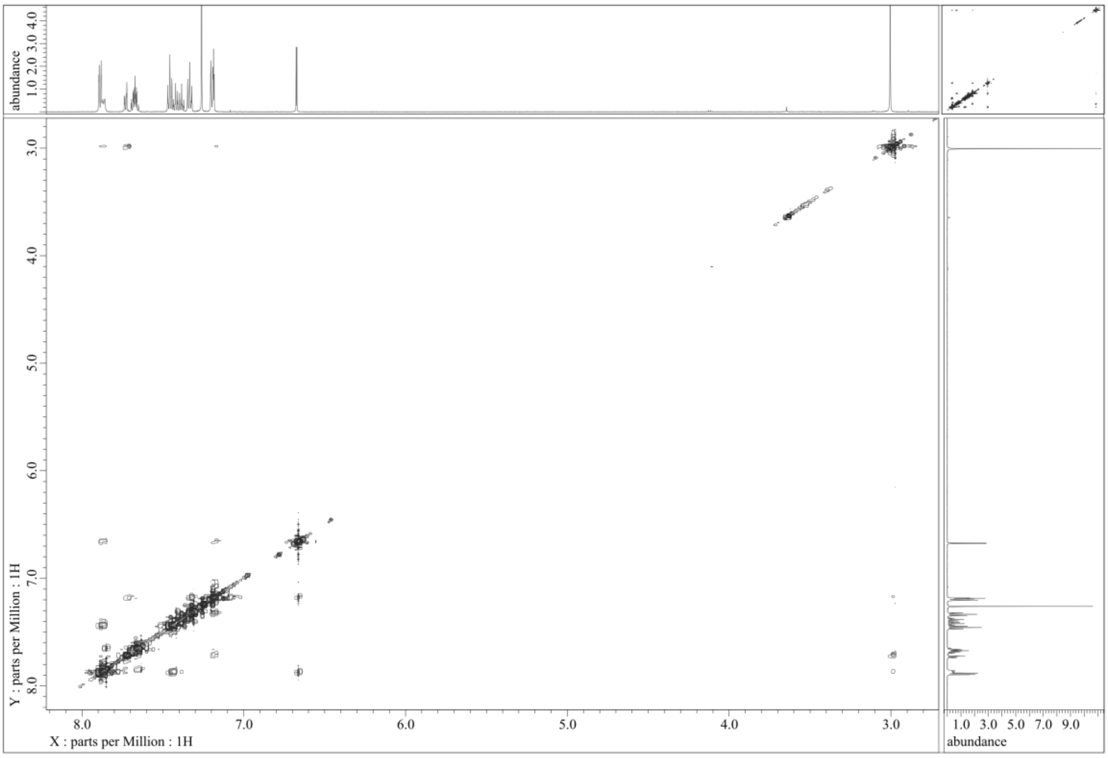

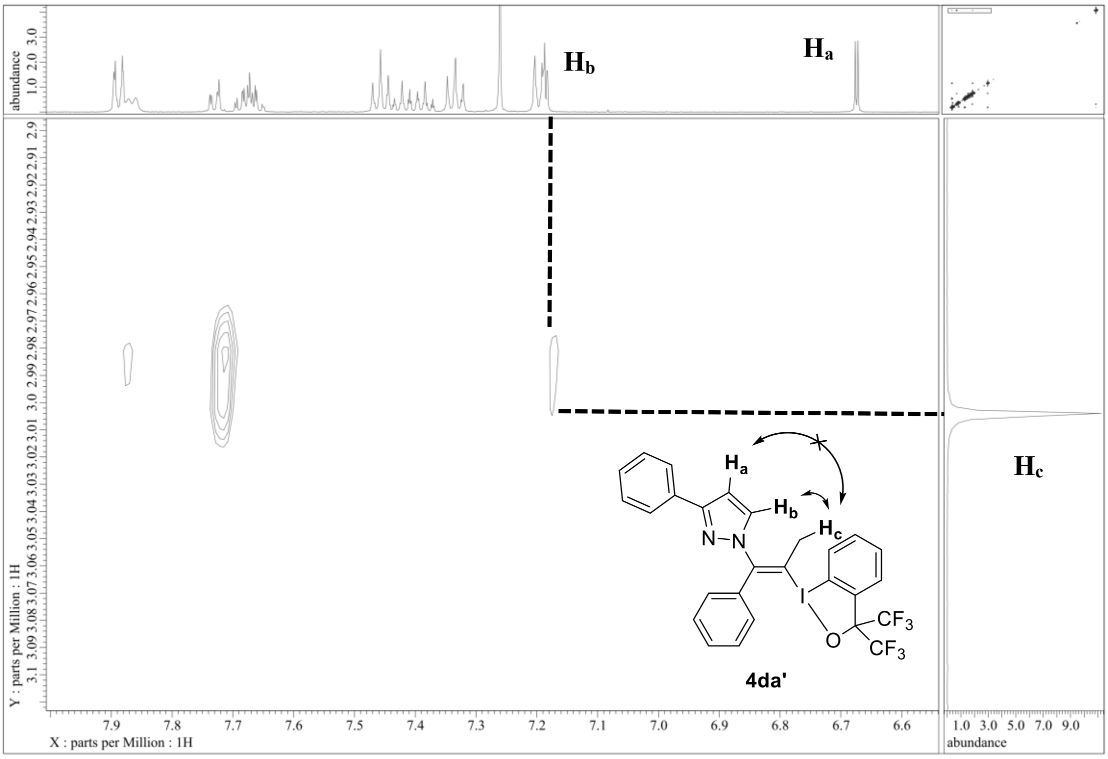

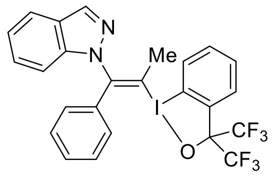

(*E*)-1-(2-(3,3-Bis(trifluoromethyl)-1λ^3-benzo[*d*][1,2]iodaoxol-1(3*H*)-yl)-1-phenylprop-1-en-1-yl)-1*H*-indazole (4ea): Synthesized by the general procedure (40.9 mg, 68%); yellow solid; R_f 0.17 (hexane/EtOAc = 1/1); m.p. 184.0-186.2 °C; ^{1}H NMR (600 MHz, $CDCl_3$) δ 7.88 (d, J = 7.2 Hz, 1H), 7.83 (s, 1H), 7.77 (d, J = 9.0 Hz, 1H), 7.75-7.67 (m, 3H), 7.62 (d, J = 8.4 Hz, 1H), 7.40 (t, J = 7.8 Hz, 1H), 7.37-7.33 (m, 1H), 7.31 (t, J = 8.0 Hz, 2H), 7.18 (d, J = 7.8 Hz, 2H), 7.12 (dd, J = 8.4 Hz, 6.6 Hz, 1H), 2.80 (s, 3H); $^{13}C\{^1H\}$ NMR (151 MHz, $CDCl_3$) δ 149.3, 148.5, 137.4, 132.7, 131.5, 130.7, 129.0, 128.8, 127.5, 127.4, 125.1, 123.8 (q, J_{C-F} = 292.0 Hz), 123.0, 121.8, 120.8, 120.4, 117.9, 110.6, 81.2-80.8 (m), 24.7 (the number of signals was less than expected by two due to signal overlap); $^{19}F\{^1H\}$ NMR (565 MHz, $CDCl_3$) δ -75.9; HRMS (FAB) *m/z*: $[M + H]^+$ calcd for $C_{25}H_{18}F_6IN_2O$ 603.0363; found, 603.0358.

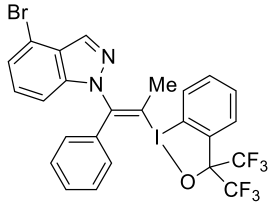

(*E*)-1-(2-(3,3-Bis(trifluoromethyl)-1λ^3-benzo[*d*][1,2]iodaoxol-1(3*H*)-yl)-1-phenylprop-1-en-1-yl)-4-bromo-1*H*-indazole (4fa): Synthesized by the general procedure (42.2 mg, 62%); white solid; R_f 0.13 (hexane/EtOAc = 5/2); m.p. 206.8-209.1 °C; ^{1}H NMR (600 MHz, $CDCl_3$) δ 7.89 (d, J = 7.2 Hz, 1H), 7.85 (s, 1H), 7.74-7.68 (m, 4H), 7.43 (t, J = 7.5 Hz, 1H), 7.33 (t, J = 8.0 Hz, 2H), 7.30 (d, J = 7.2 Hz, 1H), 7.23-7.18 (m, 3H), 2.76 (s, 3H); $^{13}C\{^1H\}$ NMR (151 MHz, $CDCl_3$) δ 149.2, 148.2, 137.0, 132.7, 131.5, 130.9, 130.8, 129.1, 128.8, 128.0, 127.3, 126.0, 125.5, 124.0, 123.8 (q, J_{C-F} = 296.4 Hz), 122.1, 117.2, 113.2, 110.6, 81.3-80.8 (m), 24.7 (the number of signals was less than expected by one due to signal overlap); $^{19}F\{^1H\}$ NMR

(565 MHz, $CDCl_3$) δ -75.9; HRMS (FAB) *m*/*z*: $[M + H]^+$ calcd for $C_{25}H_{17}BrF_6IN_2O$ 680.9468; found, 680.9479.

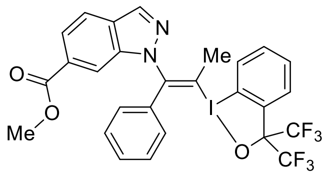

Methyl (*E*)-1-(2-(3,3-bis(trifluoromethyl)-1λ^3-benzo[*d*][1,2]iodaoxol-1(3*H*)-yl)-1-phenylprop-1-en-1-yl)-1*H*-indazole-6-carboxylate (4ga): Synthesized by the general procedure (28.4 mg, 43%); yellow solid; R_f 0.28 (hexane/EtOAc = 1/1); m.p. 196.0-197.6 °C; ^{1}H NMR (600 MHz, $CDCl_3$) δ 8.60-8.58 (m, 1H), 7.89 (d, *J* = 7.2 Hz, 1H), 7.86-7.84 (m, 1H), 7.75-7.68 (m, 4H), 7.65 (d, *J* = 9.0 Hz, 1H), 7.42 (t, *J* = 7.2 Hz, 1H), 7.33 (t, *J* = 7.8 Hz, 2H), 7.20 (d, *J* = 7.2 Hz, 2H), 3.98 (s, 3H), 2.80 (s, 3H); $^{13}C\{^1H\}$ NMR (151 MHz, $CDCl_3$) δ 167.1, 148.5, 148.1, 137.1, 132.7, 131.5, 130.9, 130.8, 129.3, 129.1, 128.8, 127.3, 125.3, 123.8 (q, J_{C-F} = 291.0 Hz), 123.5, 122.5, 122.0, 121.8, 120.5, 110.6, 81.2-80.8 (m), 52.3, 24.7 (the number of signals was less than expected by one due to signal overlap); $^{19}F\{^1H\}$ NMR (565 MHz, $CDCl_3$) δ -75.9; HRMS (FAB) *m*/*z*: $[M + H]^+$ calcd for $C_{27}H_{20}F_6IN_2O_3$ 661.0417; found, 661.0414.

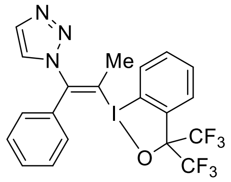

(*E*)-1-(2-(3,3-Bis(trifluoromethyl)-1λ^3-benzo[*d*][1,2]iodaoxol-1(3*H*)-yl)-1-phenylprop-1-en-1-yl)-1*H*-1,2,3-triazole (4ha): Synthesized by the general procedure (33.1 mg, 60%); white solid; R_f 0.38 (EtOAc); m.p. 160.0-162.3 °C; ^{1}H NMR (600 MHz, $CDCl_3$) δ 7.87 (d, *J* = 7.2 Hz, 1H), 7.79 (d, *J* = 1.2 Hz, 1H), 7.72-7.66 (m, 3H), 7.45-7.41 (m, 2H), 7.35 (t, *J* = 7.8 Hz, 2H), 7.16-7.14 (m, 2H), 2.75 (s, 3H); $^{13}C\{^1H\}$ NMR (151 MHz, $CDCl_3$) δ 144.3, 136.3, 133.8,

132.8, 131.4, 131.0, 130.8, 129.3, 128.8, 127.2, 124.9, 123.8 (q, $J_{C\text{-}F}$ = 292.8 Hz), 122.6, 110.4, 81.2-80.6 (m), 24.7 (the number of signals was less than expected by one due to signal overlap); $^{19}F\{^1H\}$ NMR (565 MHz, $CDCl_3$) δ -76.0; HRMS (FAB) *m/z*: $[M + H]^+$ calcd for $C_{20}H_{15}F_6IN_3O$ 554.0159; found, 554.0166.

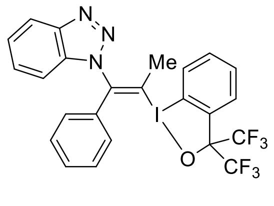

(*E*)-1-(2-(3,3-Bis(trifluoromethyl)-1λ^3-benzo[*d*][1,2]iodaoxol-1(3*H*)-yl)-1-phenylprop-1-en-1-yl)-1*H*-benzo[*d*][1,2,3]triazole (4ia): Synthesized by the general procedure (48.9 mg, 81%); white solid; R_f 0.37 (hexane/EtOAc = 1/1); m.p. 164.1-166.1 °C; ^1H NMR (600 MHz, $CDCl_3$) δ 8.13 (d, *J* = 8.4 Hz, 1H), 7.89 (d, *J* = 7.2 Hz, 1H), 7.80 (d, *J* = 7.8 Hz, 1H), 7.73 (td, *J* = 7.2 Hz, 1.2 Hz, 1H), 7.71-7.67 (m, 1H), 7.44-7.35 (m, 3H), 7.33 (t, *J* = 7.8 Hz, 2H), 7.20 (d, *J* = 6.6 Hz, 2H), 6.84 (d, *J* = 8.4 Hz, 1H), 2.71 (s, 3H); $^{13}C\{^1H\}$ NMR (151 MHz, $CDCl_3$) δ 145.7, 144.3, 135.5, 132.8, 132.7, 131.5, 131.0, 130.7 (2C), 129.1, 129.0, 128.6, 127.2, 124.6, 123.8 (q, $J_{C\text{-}F}$ = 292.0 Hz), 123.1, 120.3, 110.7, 110.5, 81.3-80.9 (m), 24.8; $^{19}F\{^1H\}$ NMR (565 MHz, $CDCl_3$) δ -75.9; HRMS (FAB) *m/z*: $[M + H]^+$ calcd for $C_{24}H_{17}F_6IN_3O$ 604.0315; found, 604.0326.

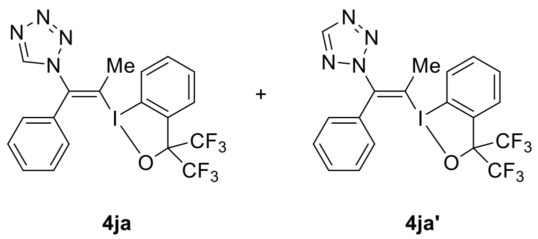

4ja **4ja'**

(*E*)-1-(2-(3,3-Bis(trifluoromethyl)-1λ^3-benzo[*d*][1,2]iodaoxol-1(3*H*)-yl)-1-phenylprop-1-en-1-yl)-1*H*-tetrazole (4ja) and (*E*)-2-(2-(3,3-bis(trifluoromethyl)-1λ^3-benzo[*d*][1,2]iodaoxol-1(3*H*)-yl)-1-phenylprop-1-en-1-yl)-2*H*-tetrazole (4ja'):

Synthesized by the general procedure and obtained as regioisomeric mixtures (40.5 mg, 73%, ratio = 7/3 as determined by ^{1}H NMR), which were separated by column chromatography on silica gel; the structural assignments for the regioisomers were made by NOESY analysis (see below); **4ja**: yellow solid; R_f 0.50 (EtOAc); m.p. 176.4-177.6 °C; ^{1}H NMR (600 MHz, $CDCl_3$) δ 8.46 (s, 1H), 7.89 (d, *J* = 6.6 Hz, 1H), 7.75-7.70 (m, 2H), 7.59-7.56 (m, 1H), 7.48 (tt, *J* = 7.8 Hz, 1.2 Hz, 1H), 7.38 (t, *J* = 7.8 Hz, 2H), 7.16 (d, *J* = 7.8 Hz, 2H), 2.76 (s, 3H); ^{13}C{^{1}H} NMR (151 MHz, $CDCl_3$) δ 143.1, 140.6, 135.3, 132.9, 131.5, 131.4, 131.0, 130.9, 129.6, 128.7, 127.2, 125.0, 123.7 (q, $J_{C\text{-}F}$ = 294.3 Hz), 110.5, 81.2-80.8 (m), 24.4; ^{19}F NMR (565 MHz, $CDCl_3$) δ -75.9; HRMS (FAB) *m*/*z*: [M + H]$^+$ calcd for $C_{19}H_{14}F_6IN_4O$ 555.0111; found, 555.0132.; **4ja'**: yellow solid; R_f 0.26 (hexane/EtOAc = 1/1); m.p. 138.1-139.6 °C; ^{1}H NMR (600 MHz, $CDCl_3$) 8.68 (s, 1H), 7.89 (d, *J* = 6.6 Hz, 1H), 7.75-7.70 (m, 2H), 7.63-7.61 (m, 1H), 7.43 (tt, *J* = 7.2 Hz, 1.2 Hz, 1H), 7.37-7.33 (m, 2H), 7.20-7.18 (m, 2H), 2.67 (s, 3H); ^{13}C{^{1}H} NMR (151 MHz, $CDCl_3$) δ 152.8, 144.3, 135.1, 132.9, 131.4, 131.0, 130.98, 130.89, 129.1, 128.7, 127.2, 126.0, 123.7 (q, $J_{C\text{-}F}$ = 291.6 Hz), 110.5, 81.4-80.6 (m), 24.2; ^{19}F{^{1}H} NMR (565 MHz, $CDCl_3$) δ -75.9; HRMS (FAB) *m*/*z*: [M + H]$^+$ calcd for $C_{19}H_{14}F_6IN_4O$ 555.0111; found, 555.0124.

NOESY spectra of **4ja**

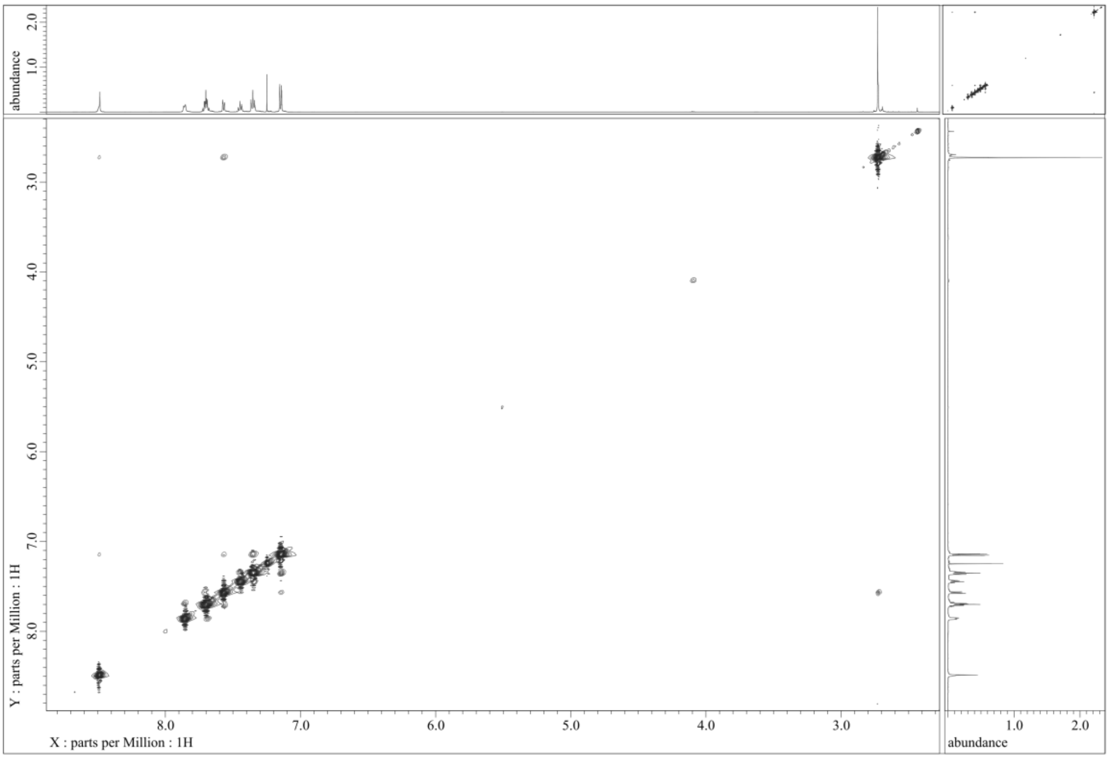

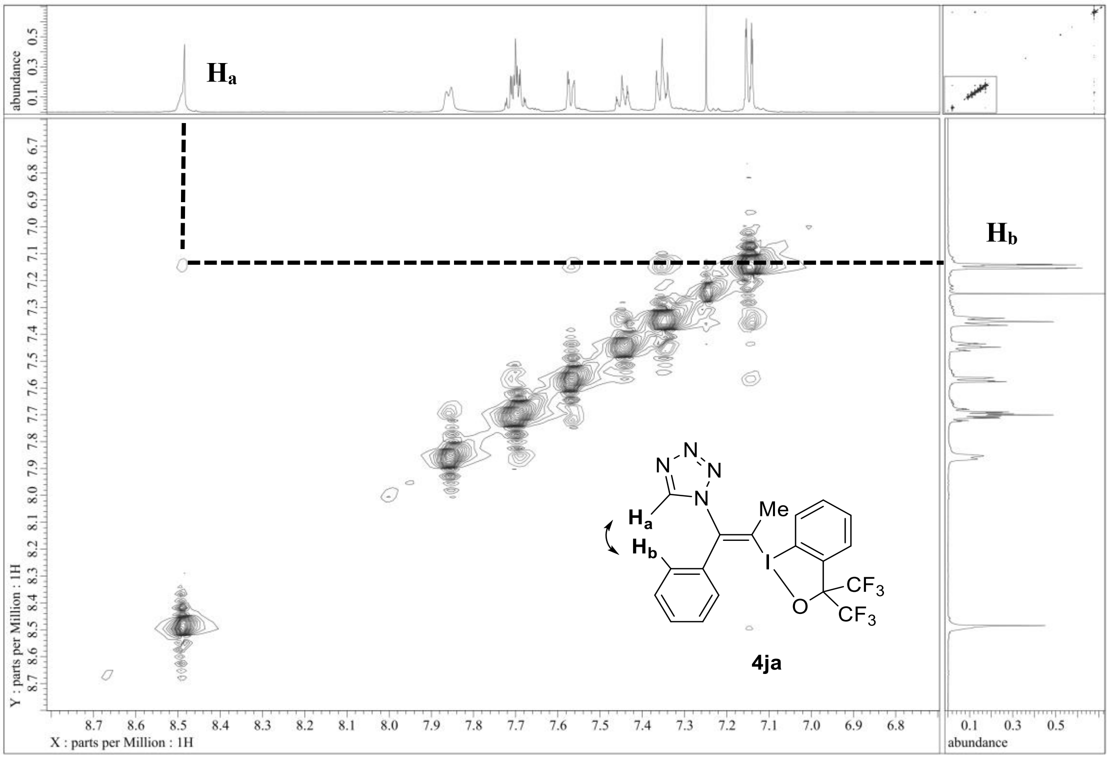

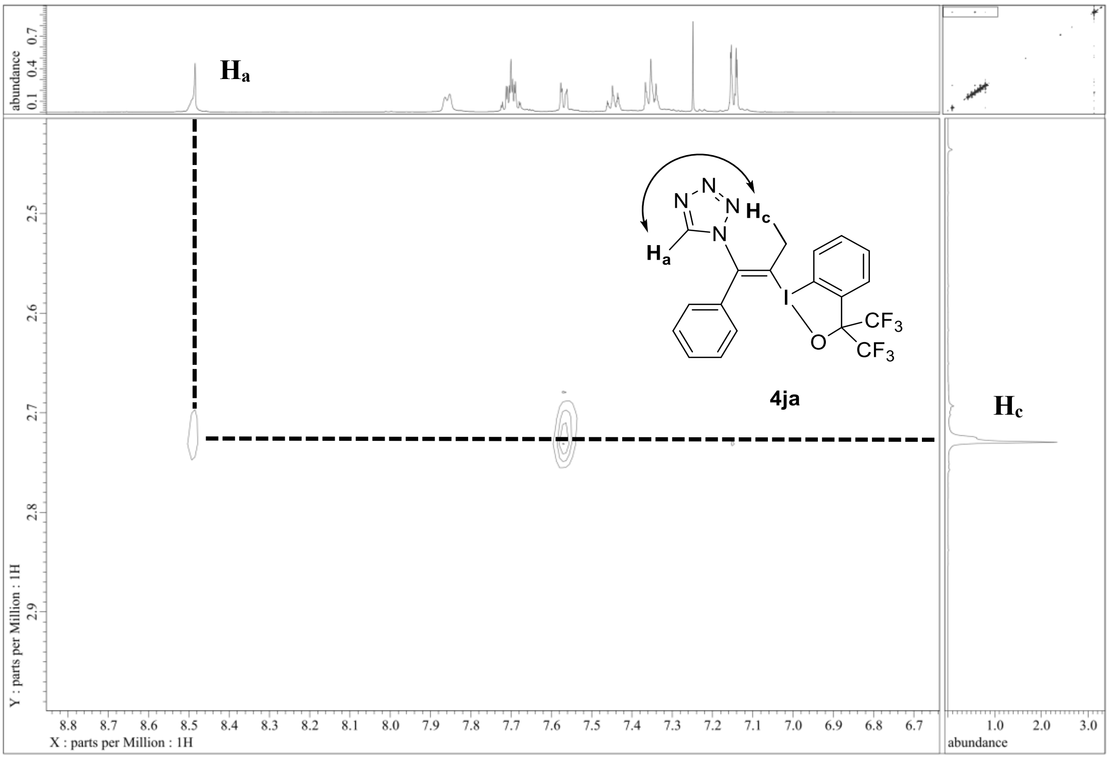

Ha
Hc
4ja
abundance
Y : parts per Million : 1H
X : parts per Million : 1H
CF3

NOESY spectra of **4ja’**

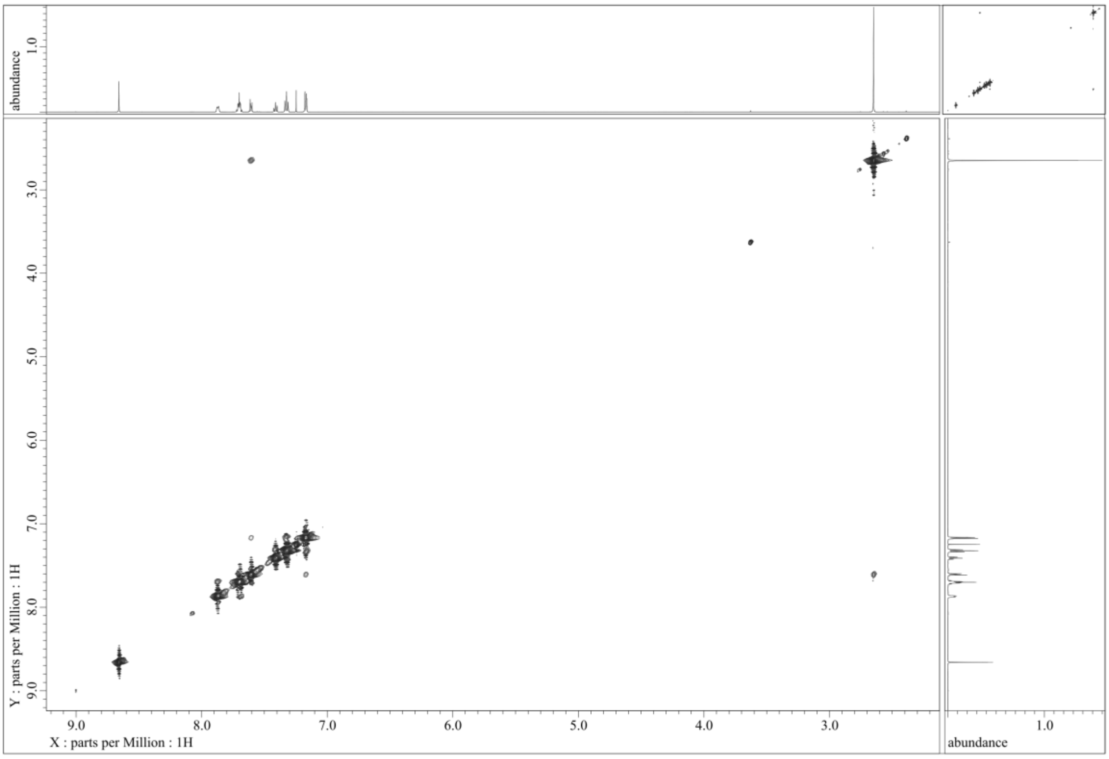

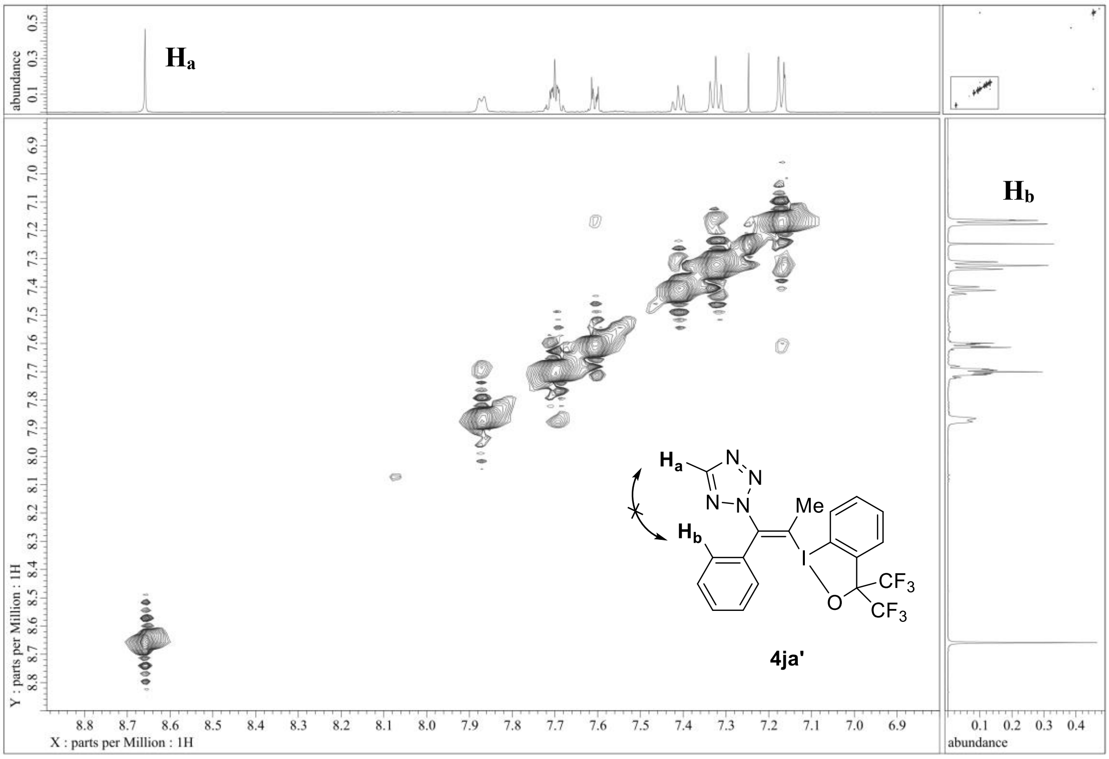

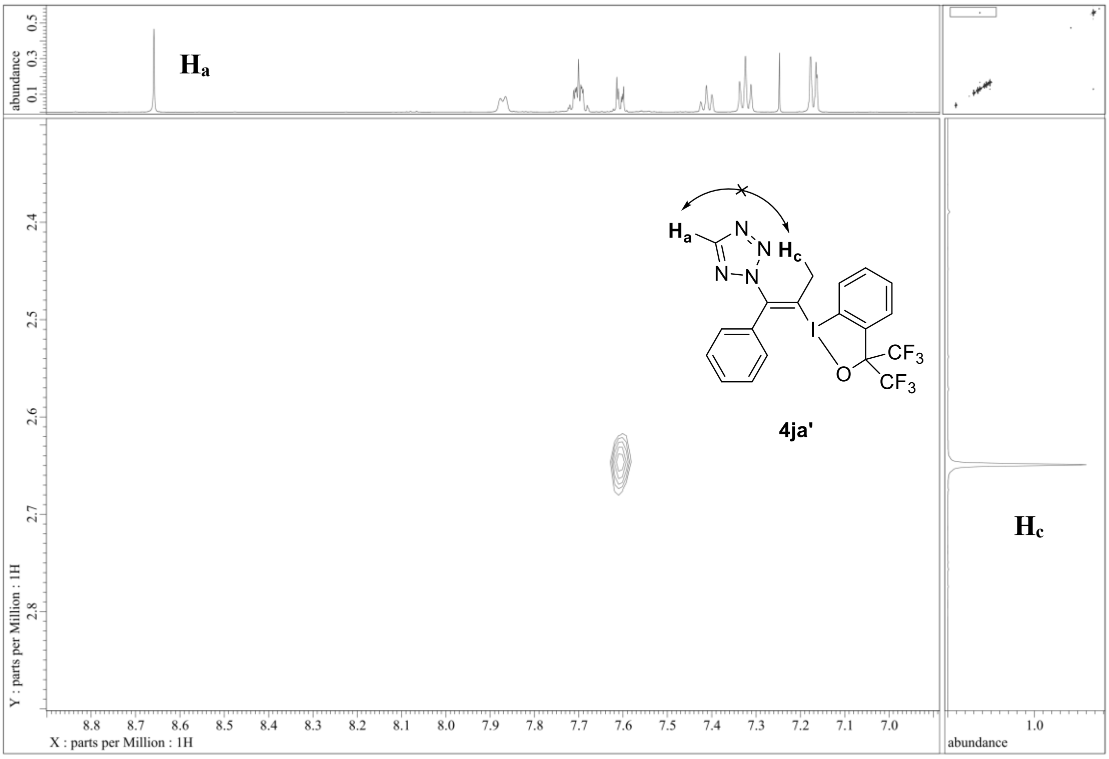
H_a
H_c
4ja'
CF_3
CF_3
X : parts per Million : 1H
Y : parts per Million : 1H
abundance

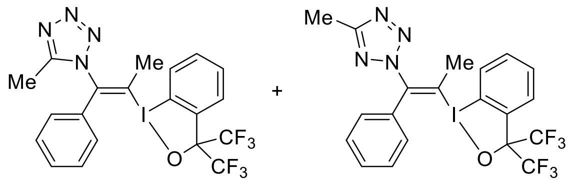

4ka 4ka'

(E)-1-(2-(3,3-Bis(trifluoromethyl)-1λ^3-benzo[*d*][1,2]iodaoxol-1(3*H*)-yl)-1-phenylprop-1-en-1-yl)-5-methyl-1*H*-tetrazole (4ka) and (E)-2-(2-(3,3-bis(trifluoromethyl)-1λ^3-benzo[*d*][1,2]iodaoxol-1(3*H*)-yl)-1-phenylprop-1-en-1-yl)-5-methyl-2*H*-tetrazole (4ka'): Synthesized by the general procedure and obtained as regioisomeric mixtures (54.0 mg, 95%, ratio = 7/3 as determined by ^{1}H NMR), which were separated by column chromatography on silica gel; the structural assignments for the regioisomers were made by analogy with compound **4ja** and **4ja'**; **4ka**: white solid; R_f 0.53 (hexane/EtOAc = 1/2); m.p. 172.4-174.6 °C; ^{1}H NMR (600 MHz, $CDCl_3$) δ 7.87 (d, J = 7.2 Hz, 1H), 7.76-7.72 (m, 1H), 7.72-7.68 (m, 1H), 7.65 (dd, J = 8.4 Hz, 1.2 Hz, 1H), 7.43 (tt, J = 7.2 Hz, 1.2 Hz, 1H), 7.38-7.33 (m, 2H), 7.23-7.19 (m, 2H), 2.54 (s, 3H), 2.28 (s, 3H); $^{13}C\{^1H\}$ NMR (151 MHz, $CDCl_3$) δ 152.0, 141.5, 134.3, 133.0, 131.4, 131.2, 131.0, 130.9, 129.3, 128.8 127.6, 127.1, 123.7 (q, $J_{C\text{-}F}$ = 291.7 Hz), 110.1, 81.2-80.8 (m), 24.3, 9.3; $^{19}F\{^1H\}$ NMR (565 MHz, $CDCl_3$) δ -75.9; HRMS (FAB) *m*/*z*: $[M + H]^+$ calcd for $C_{20}H_{16}F_6IN_4O$ 569.0267; found, 569.0280.; **4ka'**; white solid; R_f 0.35 (hexane/EtOAc = 1/1); m.p. 163.0-165.0 °C; ^{1}H NMR (600 MHz, $CDCl_3$) δ 7.88 (d, J = 7.2 Hz, 1H), 7.73-7.68 (m, 2H), 7.63-7.60 (m, 1H), 7.42 (tt, J = 7.2 Hz, 0.6 Hz, 1H), 7.33 (t, J = 7.8 Hz, 2H), 7.19-7.16 (m, 2H), 2.68 (s, 3H), 2.61 (s, 3H); $^{13}C\{^1H\}$ NMR (151 MHz, $CDCl_3$) δ 163.2, 144.6, 135.3, 132.9, 131.4, 131.0, 130.90, 130.87, 129.1, 128.7, 127.3, 125.2, 123.7 (q, J = 291.3 Hz), 110.5, 81.2-80.8 (m), 24.3, 11.0; $^{19}F\{^1H\}$ NMR (565 MHz, $CDCl_3$) δ -75.9; HRMS (FAB) *m*/*z*: $[M + H]^+$ calcd for $C_{20}H_{16}F_6IN_4O$ 569.0267; found 569.0246.

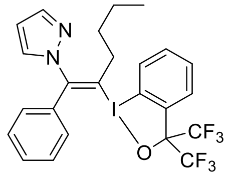

(*E*)-1-(2-(3,3-Bis(trifluoromethyl)-1λ^3-benzo[*d*][1,2]iodaoxol-1(3*H*)-yl)-1-phenylhex-1-en-1-yl)-1*H*-pyrazole (4ab): Synthesized by the general procedure (23.6 mg, 40%); white solid; R_f 0.55 (hexane/EtOAc = 1/1); m.p. 129.9-131.2 °C; ^{1}H NMR (600 MHz, $CDCl_3$) δ 7.82 (d, J = 7.2 Hz, 1H), 7.76-7.73 (m, 2H), 7.66-7.60 (m, 2H), 7.38 (t, J = 7.5 Hz, 1H), 7.32-7.28 (m, 2H), 7.19 (d, J = 1.8 Hz, 1H), 7.15-7.11 (m, 2H), 6.37-6.36 (m, 1H), 3.10-2.90 (m, 2H), 1.70-1.61 (m, 2H), 1.40-1.31 (m, 2H), 0.88 (t, J = 7.5 Hz, 3H); ^{13}C{^{1}H} NMR (151 MHz, $CDCl_3$) δ 147.7, 141.2, 137.9, 132.4, 131.9, 131.7, 130.6, 130.5, 129.1, 129.0, 127.6, 127.2, 125.0, 124.0 (q, $J_{C\text{-}F}$ = 292.8 Hz), 111.1, 107.3, 81.4-81.0 (m), 36.3, 32.1, 22.4, 13.7; ^{19}F{^{1}H} NMR (565 MHz, $CDCl_3$) δ -75.8; HRMS (FAB) *m*/*z*: [M + H]$^+$ calcd for $C_{24}H_{22}F_6IN_2O$ 595.0676; found, 595.0691.

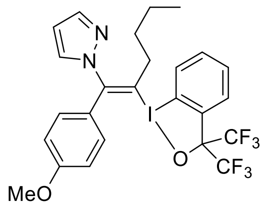

(*E*)-1-(2-(3,3-Bis(trifluoromethyl)-1λ^3-benzo[*d*][1,2]iodaoxol-1(3*H*)-yl)-1-(4-methoxyphenyl)hex-1-en-1-yl)-1*H*-pyrazole (4ac): Synthesized by the general procedure (33.1 mg, 53%); white solid; R_f 0.20 (hexane/EtOAc = 3/2); m.p. 42.4-44.0 °C; ^{1}H NMR (600 MHz, $CDCl_3$) δ 7.83 (d, J = 7.2 Hz, 1H), 7.75 (dd, J = 8.4 Hz, 1.8 Hz, 1H), 7.73 (d, J = 1.8 Hz, 1H), 7.66-7.60 (m, 2H), 7.22 (d, J = 2.4 Hz, 1H), 7.08-7.05 (m, 2H), 6.81-6.78 (m, 2H), 6.38-6.36 (m, 1H), 3.78 (s, 3H), 3.10-2.70 (m, 2H), 1.67-1.61 (m, 2H), 1.37-1.30 (m, 2H), 0.87 (t, J = 7.2 Hz, 3H); ^{13}C{^{1}H} NMR (151 MHz, $CDCl_3$) δ 161.3, 147.8, 141.1, 132.2, 131.8, 131.6, 130.7, 130.5, 130.3, 130.0, 127.6, 123.9 (q, J = 292.5 Hz), 123.7, 114.3, 111.0, 107.2, 81.3-80.9 (m), 55.4, 36.3, 32.0, 22.3, 13.6; ^{19}F{^{1}H} NMR (565 MHz, $CDCl_3$) δ -75.9; HRMS (FAB)

m/z: $[M + H]^+$ calcd for $C_{25}H_{24}F_6IN_2O_2$ 625.0781; found, 625.0776.

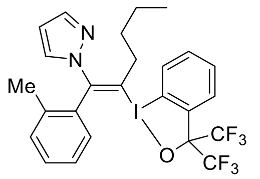

(*E*)-1-(2-(3,3-Bis(trifluoromethyl)-1λ³-benzo[*d*][1,2]iodaoxol-1(3*H*)-yl)-1-(*o*-tolyl)hex-1-en-1-yl)-1*H*-pyrazole (4ad): Synthesized by the general procedure (21.9 mg, 36%); white solid; R_f 0.13 (hexane/EtOAc = 4/1); m.p. 115.1-117.3 °C; ^{1}H NMR (600 MHz, $CDCl_3$) δ 7.81 (d, J = 7.2 Hz, 1H), 7.77-7.72 (m, 2H), 7.65-7.59 (m, 2H), 7.32 (t, J = 7.2 Hz, 1H), 7.20 (d, J = 7.8 Hz, 1H), 7.15-7.07 (m, 2H), 7.05 (d, J = 2.4 Hz, 1H), 6.32-6.30 (m, 1H), 3.50-2.95 (m, 2H), 1.97 (s, 3H), 1.76-1.67 (m, 2H), 1.43-1.34 (m, 2H), 0.89 (t, J = 7.2 Hz, 3H); ^{13}C{^{1}H} NMR (151 MHz, $CDCl_3$) δ 146.6, 141.3, 137.7, 137.0, 132.2, 131.9, 131.38, 131.35, 130.7, 130.5, 130.3, 129.7, 127.8, 126.5, 124.3, 124.0 (q, J = 288.1 Hz), 110.9, 107.3, 81.4-81.0 (m), 35.8, 32.2, 22.3, 18.8, 13.6; ^{19}F{^{1}H} NMR (565 MHz, $CDCl_3$) δ -75.7 (q, J = 8.5 Hz), -76.2 (q, J = 7.9 Hz); HRMS (FAB) m/z: $[M + H]^+$ calcd for $C_{25}H_{24}F_6IN_2O$ 609.0832; found, 609.0848.

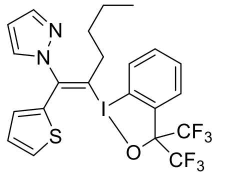

(*E*)-1-(2-(3,3-Bis(trifluoromethyl)-1λ³-benzo[*d*][1,2]iodaoxol-1(3*H*)-yl)-1-(thiophen-2-yl)hex-1-en-1-yl)-1*H*-pyrazole (4ae): Synthesized by the general procedure (34.2 mg, 57%); yellow solid; R_f 0.55 (hexane/EtOAc = 1/1); m.p. 43.2-45.0 °C; ^{1}H NMR (600 MHz, $CDCl_3$) δ 7.86 (d, J = 6.0 Hz, 1H), 7.75-7.73 (m, 1H), 7.70-7.67 (m, 1H), 7.65-7.61 (m, 2H), 7.39 (d, J = 2.4 Hz, 1H), 7.37-7.34 (m, 1H), 6.97-6.93 (m, 2H), 6.43-6.40 (m, 1H), 2.90-2.70 (m, 2H), 1.66-1.60 (m, 2H), 1.36-1.28 (m, 2H), 0.86 (t, J = 7.5 Hz, 3H); ^{13}C{^{1}H} NMR (151 MHz, $CDCl_3$) δ 141.4, 141.2, 139.0, 132.4, 131.5, 131.3, 130.8, 130.6, 130.5, 129.4, 127.5, 127.2, 127.1, 123.9 (q, J_{C-F} = 292.0 Hz), 111.2, 107.3, 81.5-80.7 (m), 36.7, 31.9, 22.4, 13.6; ^{19}F{^{1}H}

NMR (565 MHz, $CDCl_3$) δ -75.9; HRMS (FAB) *m/z*: $[M + H]^+$ calcd for $C_{22}H_{20}F_6IN_2OS$ 601.0240; found, 601.0257.

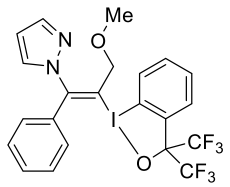

(*E*)-1-(2-(3,3-Bis(trifluoromethyl)-1λ^3-benzo[*d*][1,2]iodaoxol-1(3*H*)-yl)-3-methoxy-1-phenylprop-1-en-1-yl)-1*H*-pyrazole (4af): Synthesized by the general procedure (19.2 mg, 33%); white solid; R_f 0.29 (hexane/EtOAc = 1/1); m.p. 134.0-136.0 °C; ^{1}H NMR (600 MHz, $CDCl_3$) δ 7.78-7.74 (m, 3H), 7.60-7.56 (m, 2H), 7.38 (tt, *J* = 7.8 Hz, 1.2 Hz, 1H), 7.29-7.25 (m, 3H), 7.12 (d, *J* = 7.8 Hz, 2H), 6.39-6.38 (m, 1H), 4.62 (s, 2H), 3.39 (s, 2H); $^{13}C\{^1H\}$ NMR (151 MHz, $CDCl_3$) δ 147.8, 141.7, 136.6, 132.0, 131.9, 131.7, 130.8, 130.1, 130.0, 129.2, 128.8, 128.1, 123.9 (q, $J_{C\text{-}F}$ = 291.7 Hz), 120.1, 112.6, 107.7, 81.4-81.0 (m), 72.7, 58.9; $^{19}F\{^1H\}$ NMR (565 MHz, $CDCl_3$) δ -76.0; HRMS (FAB) *m/z*: $[M + H]^+$ calcd for $C_{22}H_{18}F_6IN_2O_2$ 583.0312; found, 583.0303.

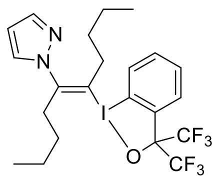

(*E*)-1-(6-(3,3-Bis(trifluoromethyl)-1λ^3-benzo[*d*][1,2]iodaoxol-1(3*H*)-yl)dec-5-en-5-yl)-1*H*-pyrazole (4ag): Synthesized by the general procedure (35.7 mg, 62%); yellow oil; R_f 0.35 (hexane/EtOAc = 1/1); ^{1}H NMR (600 MHz, $CDCl_3$) δ 7.91 (d, *J* = 7.8 Hz, 1H), 7.72-7.71 (m, 1H), 7.65 (t, *J* = 7.8 Hz, 1H), 7.61 (td, *J* = 7.8 Hz, 1.5 Hz, 1H), 7.57 (d, *J* = 7.8 Hz, 1H), 7.50 (d, *J* = 1.8 Hz, 1H), 6.44 (t, *J* = 7.8 Hz, 1H), 2.97-2.86 (m, 2H), 2.56-2.47 (m, 2H), 1.51-1.45 (m, 2H), 1.29-1.18 (m, 6H), 0.81 (t, *J* = 7.5 Hz, 3H), 0.77 (t, *J* = 7.0 Hz, 3H); $^{13}C\{^1H\}$ NMR (151 MHz, $CDCl_3$) δ 150.1, 140.8, 132.5, 131.5, 130.8, 130.7, 130.1, 127.3, 124.7, 124.0 (q, $J_{C\text{-}F}$ = 294.1 Hz), 109.9, 106.8, 81.4-80.7 (m), 38.3, 35.5, 32.0, 29.5, 22.3, 22.0, 13.55, 13.52;

$^{19}F\{^1H\}$ NMR (565 MHz, $CDCl_3$) δ -75.8; HRMS (FAB) *m*/*z*: $[M + H]^+$ calcd for $C_{22}H_{26}F_6IN_2O$ 575.0989; found, 575.1000.

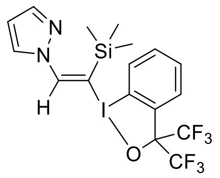

(*E*)-1-(2-(3,3-Bis(trifluoromethyl)-1λ^3-benzo[*d*][1,2]iodaoxol-1(3*H*)-yl)-2-(trimethylsilyl)vinyl)-1*H*-pyrazole (4ah): Synthesized by the general procedure (14.4 mg, 27%); white solid; R_f 0.22 (hexane/EtOAc = 1/1); m.p. 128.7-130.5 °C; ^1H NMR (600 MHz, $CDCl_3$) δ 8.20-8.18 (m, 1H), 7.86 (d, *J* = 7.2 Hz, 1H), 7.76-7.74 (m, 1H), 7.62-7.57 (m, 2H), 7.57-7.50 (m, 2H), 6.48-6.47 (m, 1H), 0.35 (m, 9H); $^{13}C\{^1H\}$ NMR (151 MHz, $CDCl_3$) δ 149.1, 142.7, 131.9, 131.7, 131.2, 130.4, 130.3, 127.4, 124.1 (q, *J* = 291.3 Hz), 114.3, 111.7, 108.7, 81.4-81.0 (m), 2.1; $^{19}F\{^1H\}$ NMR (565 MHz, $CDCl_3$) δ -75.4, -76.5; HRMS (FAB) *m*/*z*: $[M + H]^+$ calcd for $C_{17}H_{18}F_6IN_2OSi$ 535.0132; found, 535.0138.

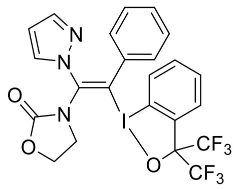

(*E*)-3-(2-(3,3-Bis(trifluoromethyl)-1λ^3-benzo[*d*][1,2]iodaoxol-1(3*H*)-yl)-2-phenyl-1-(1*H*-pyrazol-1-yl)vinyl)oxazolidin-2-one (4ai): Synthesized by the general procedure (30.5 mg, 49%); white solid; R_f 0.34 (Et_2O); m.p. 150.4-151.5 °C; ^1H NMR (600 MHz, $CDCl_3$) δ 7.78 (d, *J* = 8.4 Hz, 1H), 7.76 (d, *J* = 7.8 Hz, 1H), 7.66 (d, *J* = 1.8 Hz, 1H), 7.55 (t, *J* = 7.2 Hz, 1H), 7.48-7.45 (m, 1H), 7.26-7.22 (m, 3H), 7.22-7.19 (m, 2H), 7.13 (d, *J* = 2.4 Hz, 1H), 6.20-6.18 (m, 1H), 4.47 (t, *J* = 7.8 Hz, 2H), 3.73 (t, *J* = 7.8 Hz, 2H); $^{13}C\{^1H\}$ NMR (151 MHz, $CDCl_3$) δ 156.2, 142.3, 136.6, 135.7, 132.1, 131.84, 131.79, 130.6, 130.1, 129.4, 129.1, 128.8, 124.0 (q, J_{C-F} = 292.0 Hz), 119.3, 111.5, 108.2, 81.6-81.2 (m), 62.9, 45.1; $^{19}F\{^1H\}$ NMR (565 MHz, $CDCl_3$) δ -75.7; HRMS (FAB) *m*/*z*: $[M + H]^+$ calcd for $C_{23}H_{17}F_6IN_3O_3$ 624.0213; found,

624.0215.

3. Product Transformations

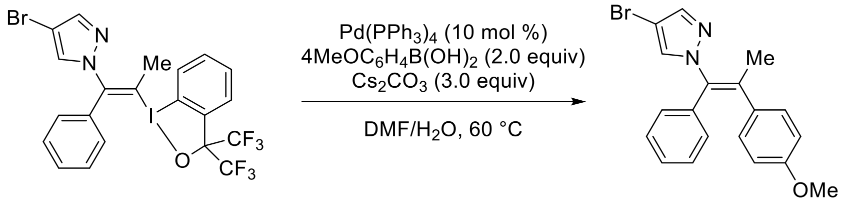

4ba **5**, 47%

(*E*)-4-Bromo-1-(2-(4-methoxyphenyl)-1-phenylprop-1-en-1-yl)-1*H*-pyrazole (5): Under an argon atmosphere, a 4 mL vial equipped with a stir bar was charged with **4ba** (63.1 mg, 0.10 mmol), $Pd(PPh_3)_4$ (11.6 mg, 0.010 mmol, 10 mol %) and Cs_2CO_3 (97.7 mg, 0.30 mmol, 3.0 equiv), followed by the addition of DMF (0.80 mL) and H_2O (0.20 mL). Then (4-methoxyphenyl)boronic acid (30.4 mg, 0.20 mmol, 2.0 equiv) was added. The resulting mixture was placed on an aluminum block preheated at 60 °C and stirred for 18 h. The mixture was cooled to room temperature, diluted with Et_2O (10 mL), and washed with H_2O (5 mL) and brine (5 mL). The organic layer was dried over Na_2SO_4 and concentrated under reduced pressure. The residue was purified by flash column chromatography on silica gel to afford the title compound **5** as a yellow oil (17.4 mg, 47%).

R_f 0.25 (hexane/EtOAc = 10/1); ^{1}H NMR (600 MHz, $CDCl_3$) δ 7.64 (s, 1H), 7.48 (s, 1H), 7.13-7.07 (m, 5H), 6.90-6.86 (m, 2H), 6.76-7.72 (m, 2H), 3.77 (s, 3H), 2.05 (s, 3H); $^{13}C\{^1H\}$ NMR (151 MHz, $CDCl_3$) δ 158.9, 140.4, 137.1, 136.4, 134.8, 132.9, 131.1, 130.1, 129.5, 128.0, 127.6, 113.7, 93.7, 55.2, 21.6; HRMS (EI) *m*/*z*: $[M]^+$ calcd for $C_{19}H_{17}BrN_2O$ 368.0519; found, 368.0507.

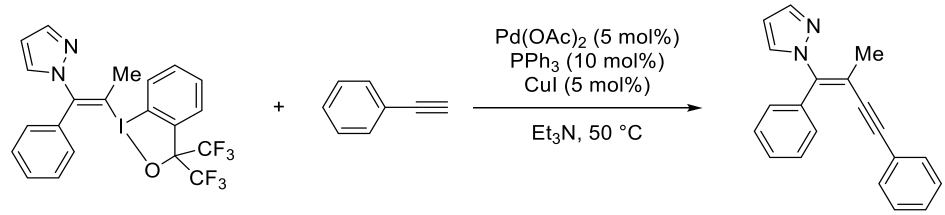

4aa (1.5 equiv) **6**, 74%

(*E*)-1-(2-Methyl-1,4-diphenylbut-1-en-3-yn-1-yl)-1*H*-pyrazole (6): Under an argon

atmosphere, a 4 mL vial equipped with a stir bar was charged with **4aa** (55.2 mg, 0.10 mmol), $Pd(OAc)_2$ (1.1 mg, 5.0 μmol, 5 mol%), PPh_3 (2.6 mg, 0.01 mmol, 10 mol %), CuI (1.0 mg, 5.3 μmol, 5 mol%), and Et_3N (0.6 mL). To the mixture was added ethynylbenzene (15.3 mg, 0.15 mmol, 1.5 equiv), and the resulting mixture was placed on an aluminum block preheated at 50 °C and stirred for 5 h. The mixture was diluted with Et_2O (10 mL) and washed with H_2O (5 mL) and brine (5 mL). The organic layer was dried over Na_2SO_4 and concentrated under reduced pressure. The residue was purified by flash column chromatography on silica gel to afford the title compound **6** as a white solid (21.0 mg, 74%).

R_f 0.38 (hexane/EtOAc = 10/1); m.p. 75.4-77.1 °C; ^{1}H NMR (600 MHz, $CDCl_3$) δ 7.71 (d, J = 1.2 Hz, 1H), 7.48 (dd, J = 8.4 Hz, 2.4 Hz, 2H), 7.45 (d, J = 3.0 Hz, 1H), 7.36-7.32 (m, 5H), 7.31-7.29 (m, 3H), 6.40 (t, J = 1.8 Hz, 1H), 2.04 (s, 3H); ^{13}C{^{1}H} NMR (151 MHz, $CDCl_3$) δ 142.7, 140.5, 136.8, 131.7, 131.4, 128.85, 128.80, 128.4, 128.3, 127.9, 123.1, 115.5, 106.2, 94.4, 90.1, 20.4; HRMS (EI) m/z: $[M]^+$ calcd for $C_{20}H_{16}N_2$ 284.1308; found, 284.1319.

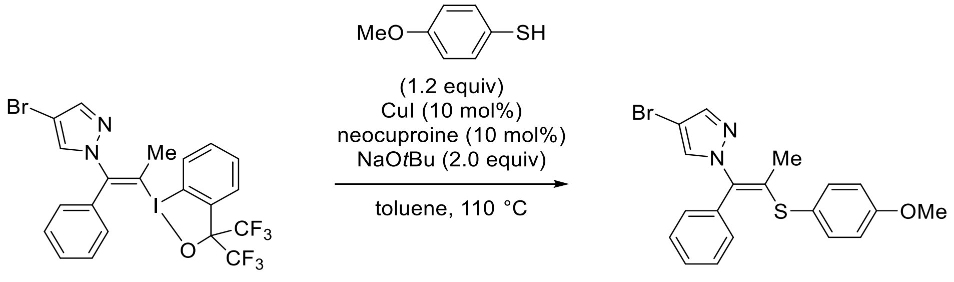

4ba **7**, 59%

(*E*)-4-Bromo-1-(2-((4-methoxyphenyl)thio)-1-phenylprop-1-en-1-yl)-1*H*-pyrazole (7): Under an argon atmosphere, a 4 mL vial equipped with a stir bar was charged with **4ba** (63.1 mg, 0.10 mmol), CuI (1.9 mg, 0.010 mmol), neocuproine (2.1 mg, 0.010 mmol), and NaO*t*-Bu (19.2 mg, 0.20 mmol), followed by the addition of toluene (1.4 mL) and 4-methoxybenzenethiol (16.8 mg, 0.12 mmol). The resulting mixture was stirred at 110 °C for 13 h. The mixture was diluted with EtOAc (10 mL) and washed with water (5 mL × 3). The organic layer was dried over Na_2SO_4 and concentrated under reduced pressure. The residue

was purified by flash column chromatography on silica gel to afford the title compound **7** as a white solid (23.7 mg, 59%).

Rf 0.2 (hexane/EtOAc = 20/1); m.p. 108.9-1102.4 °C; ^{1}H NMR (600 MHz, $CDCl_3$) δ 7.58 (s, 1H), 7.40-7.35 (m, 5H), 7.35-7.31 (m, 3H), 6.87-6.84 (m, 2H), 3.81 (s, 3H), 1.78 (s, 3H); $^{13}C\{^1H\}$ NMR (151 MHz, $CDCl_3$) δ 160.1, 140.5, 136.3, 135.7, 134.3, 134.1, 131.1, 129.2, 128.8, 128.3, 122.5, 114.7, 93.9, 55.3, 19.8; HRMS (EI) *m/z*: $[M]^+$ calcd for $C_{19}H_{17}BrN_2OS$ 400.0239; found, 400.0251.

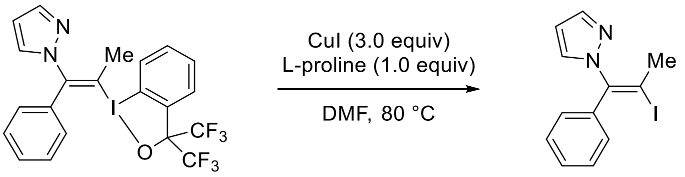

4aa **8**, 76%

(*E*)-1-(2-Iodo-1-phenylprop-1-en-1-yl)-1*H*-pyrazole (8): Under an argon atmosphere, a 4 mL vial equipped with a stir bar was charged with **4aa** (82.8 mg, 0.15 mmol), CuI (85.7 mg, 0.45 mmol), L-proline (17.3 mg, 0.15 mmol), and DMF (1.5 mL). The resulting mixture was then placed on an aluminum block preheated at 80 °C and stirred for 14 h. The mixture was cooled to room temperature, diluted with EtOAc (10 mL), and washed with H_2O (5 mL) and brine (5 mL). The organic layer was dried over Na_2SO_4 and concentrated under reduced pressure. The residue was purified by flash column chromatography on silica gel to afford the title compound **8** as a colorless oil (35.3 mg, 76%).

Rf 0.3 (hexane/EtOAc = 20/1); ^{1}H NMR (600 MHz, $CDCl_3$) δ 7.64-7.61 (m, 1H), 7.38-7.32 (m, 6H), 6.33-6.30 (m, 1H), 2.62 (s, 3H); $^{13}C\{^1H\}$ NMR (151 MHz, $CDCl_3$) δ 141.1, 140.2, 139.8, 130.7, 129.3, 128.9, 128.2, 106.2, 97.7, 30.5; HRMS (EI) *m/z*: $[M]^+$ calcd for $C_{12}H_{11}IN_2$ 309.9961; found, 309.9975.

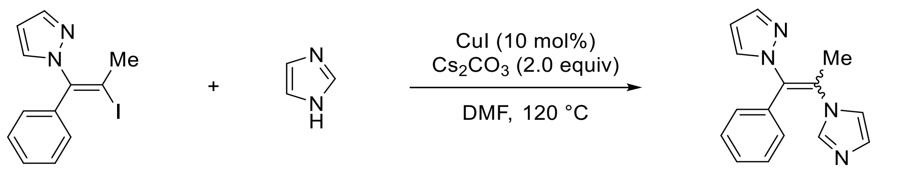

8 (2.0 equiv) **9**, 65% (ratio = 1.6:1)

1-(2-(1*H*-Imidazol-1-yl)-1-phenylprop-1-en-1-yl)-1*H*-pyrazole (9): Under an argon atmosphere, a 4 mL vial equipped with a stir bar was charged with **8** (31.1 mg, 0.10 mmol), CuI (1.9 mg, 0.01 mmol, 10 mol %), Cs_2CO_3 (65.2 mg, 0.2 mmol), imidazole (13.6 mg, 0.2 mmol), and DMF (0.5 mL). The resulting mixture was stirred at room temperature for 15 h. The mixture was diluted with Et_2O (10 mL) and washed with H_2O (5 mL) and brine (5 mL). The organic layer was dried over Na_2SO_4 and concentrated under reduced pressure. The residue was purified by flash column chromatography on silica gel to afford the title compound **9** as a yellow solid (16.3 mg, 65%, stereoisomer ratio = 1.6:1).

R_f 0.3 (hexane/EtOAc = 4/1); m.p. 113.2-115.4 °C; ^{1}H NMR (600 MHz, $CDCl_3$, major) δ 7.74-7.72 (m, 1H), 7.44-7.37 (m, 2H), 7.24-7.17 (m, 3H), 7.06-7.03 (m, 1H), 6.95-6.91 (m, 1H), 6.84-6.80 (m, 2H), 6.44-6.42 (m, 1H), 2.21 (s, 3H); ^{13}C{^{1}H} NMR (151 MHz, $CDCl_3$, major) δ 140.7, 137.0, 134.6, 134.4, 131.2, 129.8, 129.5, 129.0, 128.7, 127.9, 118.8, 107.0, 20.2; ^{1}H NMR (600 MHz, $CDCl_3$, minor) δ 7.57-7.55 (m, 1H), 7.44-7.37 (m, 3H), 7.31-7.28 (m, 3H), 7.10 (d, J = 2.4 Hz, 1H), 7.02-6.99 (m, 1H), 6.78-6.75 (m, 1H), 6.23-6.21 (m, 1H), 2.39 (s, 3H); ^{13}C{^{1}H} NMR (151 MHz, $CDCl_3$, minor) δ 141.2, 136.4, 135.1, 132.2, 130.8, 130.1, 129.2, 129.1, 128.7, 127.4, 118.2, 107.2, 20.0; HRMS (EI) *m/z*: $[M]^+$ calcd for $C_{15}H_{14}N_4$ 250.1213; found, 250.1217.

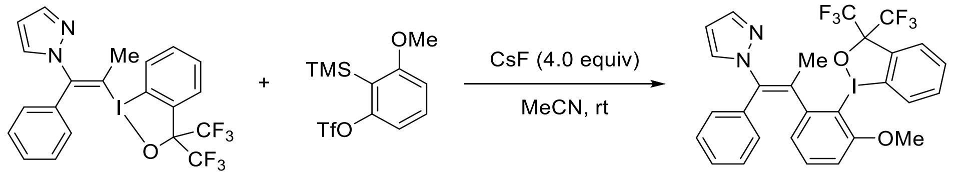

4aa (2.0 equiv) **10**, 44%

(*E*)-1-(2-(2-(3,3-Bis(trifluoromethyl)-1λ^3-benzo[*d*][1,2]iodaoxol-1(3*H*)-yl)-3-

methoxyphenyl)-1-phenylprop-1-en-1-yl)-1*H*-pyrazole (10): Under an argon atmosphere, a 4 mL vial equipped with a stir bar was charged sequentially with **4aa** (55.2 mg, 0.10 mmol) and MeCN (0.5 mL), followed by the addition of 3-methoxy-2-(trimethylsilyl)phenyl trifluoromethanesulfonate (65.7 mg, 0.20 mmol) and cesium fluoride (60.8 mg, 0.40 mmol). The resulting mixture was stirred at room temperature for 18 h. The reaction mixture was filtered through a silica plug with EtOAc as eluent and concentrated under reduced pressure. The residue was purified by flash chromatography on silica gel to afford the title compound **10** as a yellow solid (29.0 mg, 44%).

R_f 0.32 (hexane/EtOAc = 1/1); m.p. 145.8-148.0 °C; ^{1}H NMR (600 MHz, $CDCl_3$) δ 8.11 (dd, *J* = 7.8 Hz, 1.2 Hz, 1H), 7.85 (d, *J* = 7.2 Hz, 1H), 7.72 (d, *J* = 2.4 Hz, 1H), 7.70-7.63 (m, 2H), 7.43 (t, *J* = 8.4 Hz, 1H), 7.18 (d, *J* = 3.0 Hz, 1H), 7.13-7.09 (m, 2H), 7.01 (t, *J* = 7.8 Hz, 2H), 6.94 (d, *J* = 7.2 Hz, 1H), 6.65-6.62 (m, 2H), 6.28 (t, *J* = 1.8 Hz, 1H), 4.04 (s, 3H), 2.52 (s, 3H); ^{13}C{^{1}H} NMR (151 MHz, $CDCl_3$) δ 156.1, 148.0, 141.4, 140.7, 139.5, 132.1, 132.0, 130.4, 130.3, 130.2, 129.8, 129.4, 128.3, 128.0, 127.5, 124.8, 124.6, 124.1 (q, *J* = 292.0 Hz), 124.0 (q, *J* = 290.2 Hz), 118.7, 111.0, 110.9, 106.8, 81.2-80.7 (m), 56.2, 25.7; ^{19}F NMR (565 MHz, $CDCl_3$) δ -75.8 (q, *J* = 8.5 Hz), -76.0 (q, *J* = 8.6 Hz); HRMS (FAB) *m*/*z*: $[M+K]^+$ calcd for $C_{28}H_{21}F_6IKN_2O_2$ 697.0183; found, 697.0204.

4. References

(1) Ding, W.; Chai, J.; Wang, C.; Wu, J.; Yoshikai, N. *J. Am. Chem. Soc.* **2020**, *142*, 8619-8624.

(2) (a) Roesch, K. R.; Larock, R. C. *J. Org. Chem.* **2001**, *66*, 412-420. (b) Lu, B.; Li, C.; Zhang, L. *J. Am. Chem. Soc.* **2010**, *132*, 14070-14072.

(3) Peng, B.; Huang, X. L.; Xie, L. G.; Maulide, N. *Angew. Chem. Int. Ed.* **2014**, *53*, 8718-8721.

5. NMR Spectra

^{1}H NMR (600 MHz, $CDCl_3$) and $^{13}C\{^{1}H\}$ NMR (151 MHz, $CDCl_3$) spectra of **4aa**

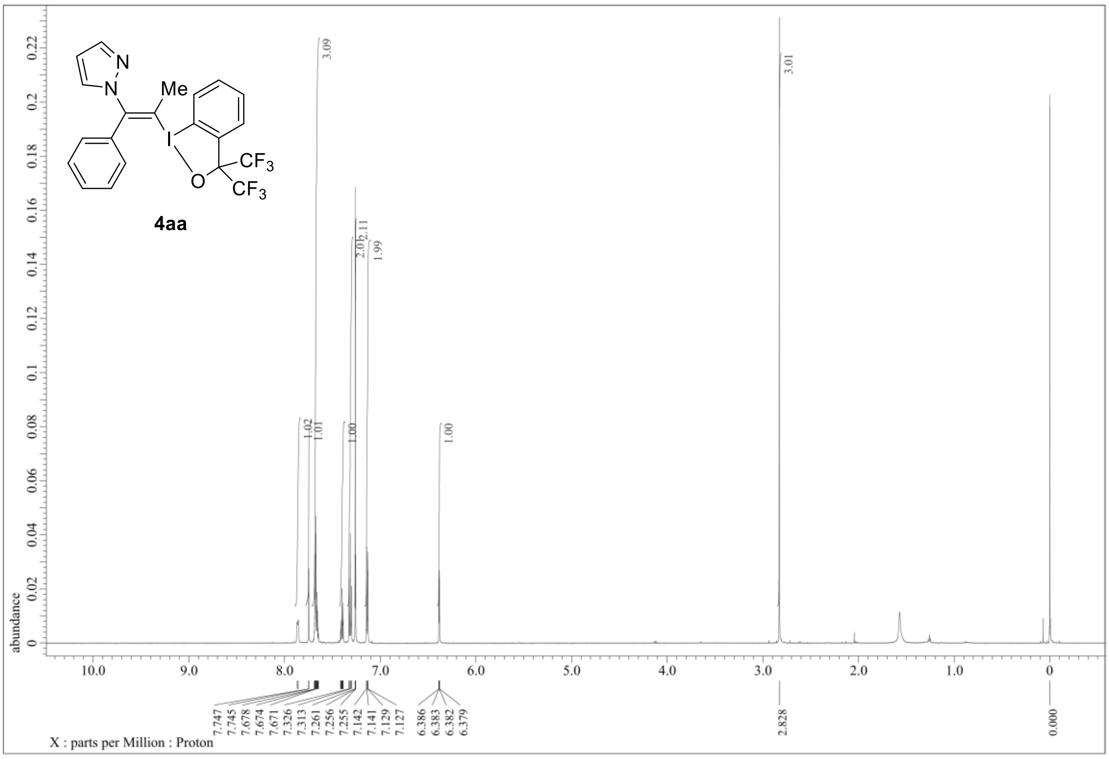

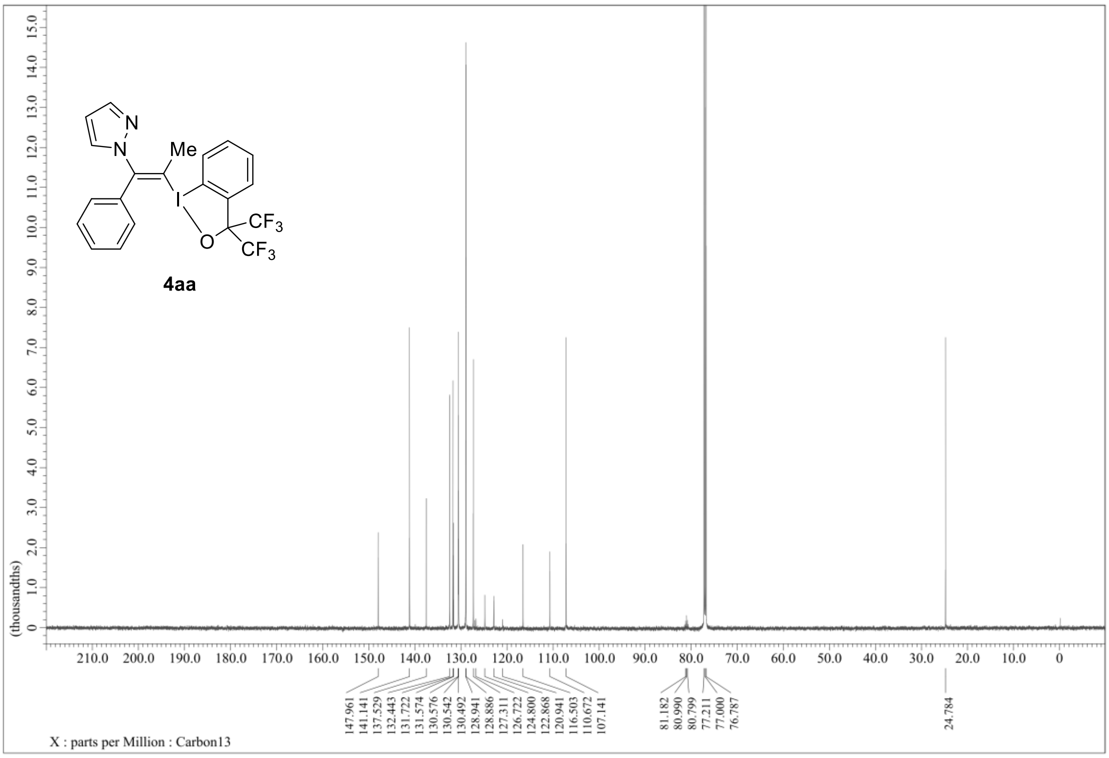

$^{19}F\{^1H\}$ NMR (565 MHz, $CDCl_3$) spectrum of **4aa**

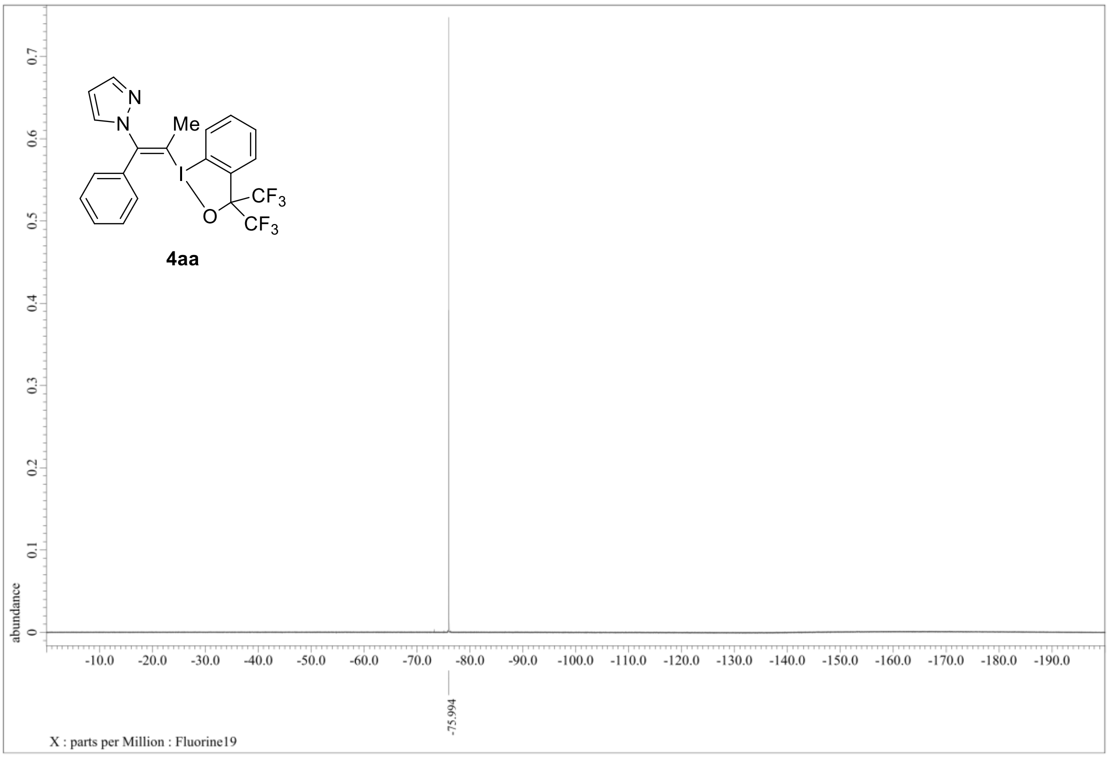

^{1}H NMR (600 MHz, $CDCl_3$) and $^{13}C\{^{1}H\}$ NMR (151 MHz, $CDCl_3$) spectra of **4ba**

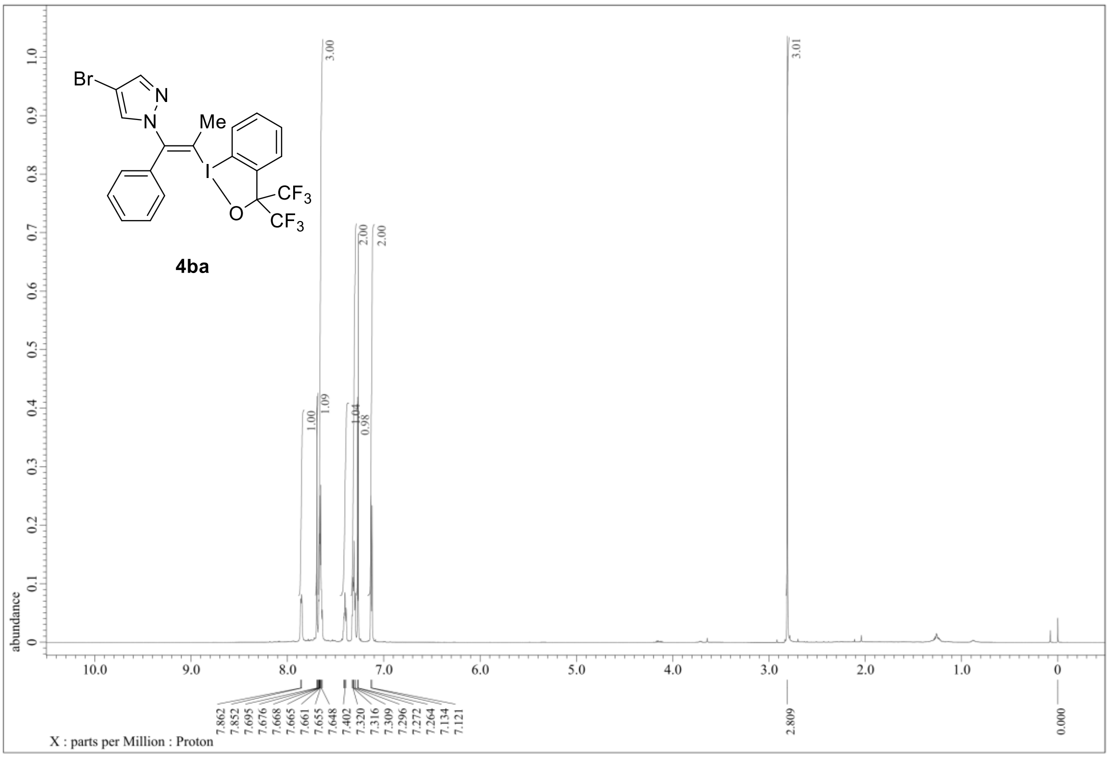

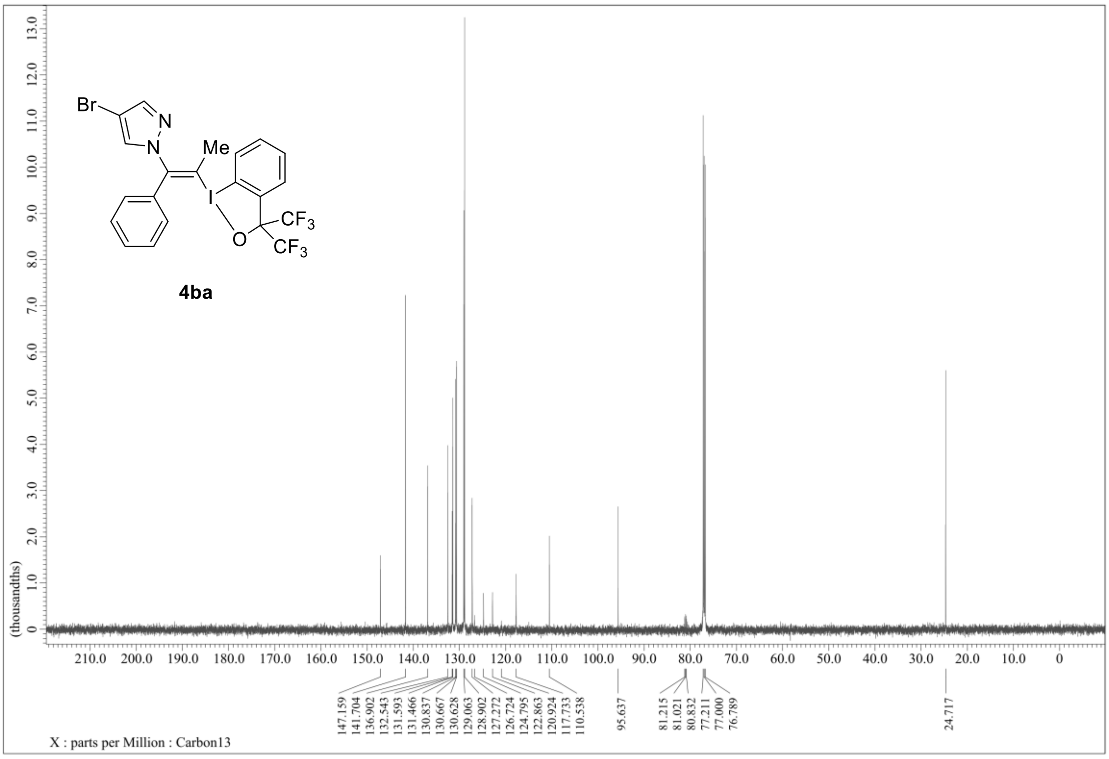

$^{19}F\{^1H\}$ NMR (565 MHz, $CDCl_3$) spectrum of **4ba**

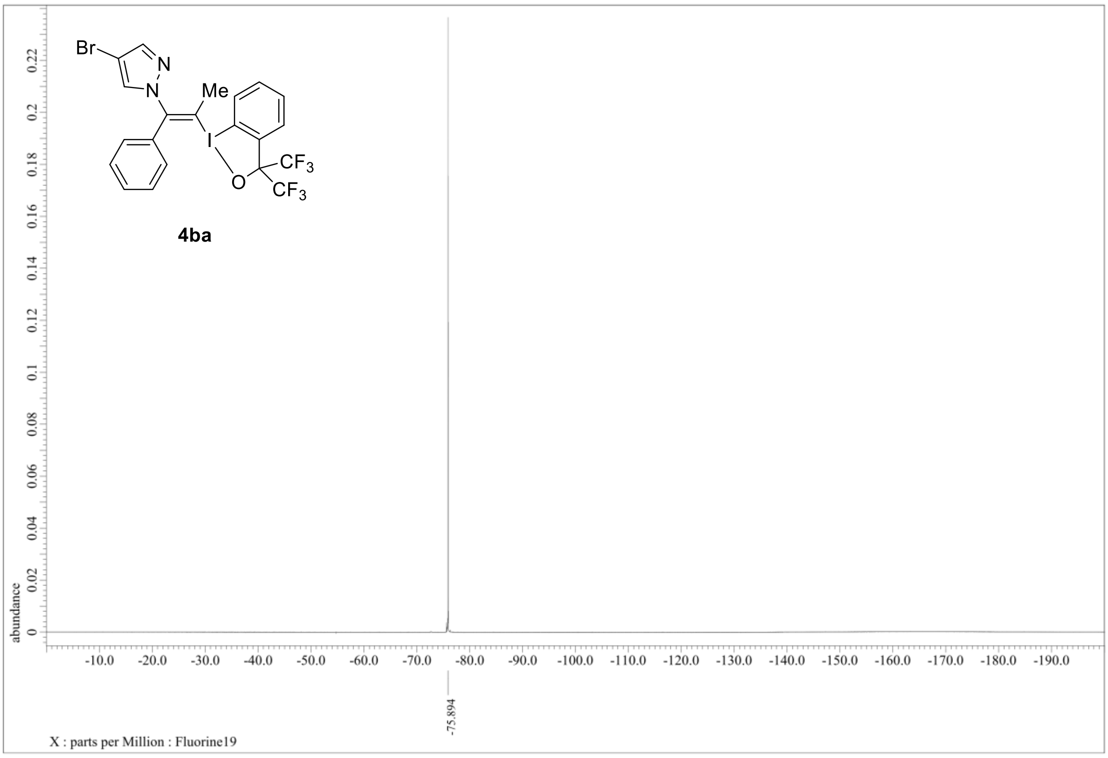

^{1}H NMR (600 MHz, $CDCl_3$) and ^{13}C{^{1}H} NMR (151 MHz, $CDCl_3$) spectra of **4ca**

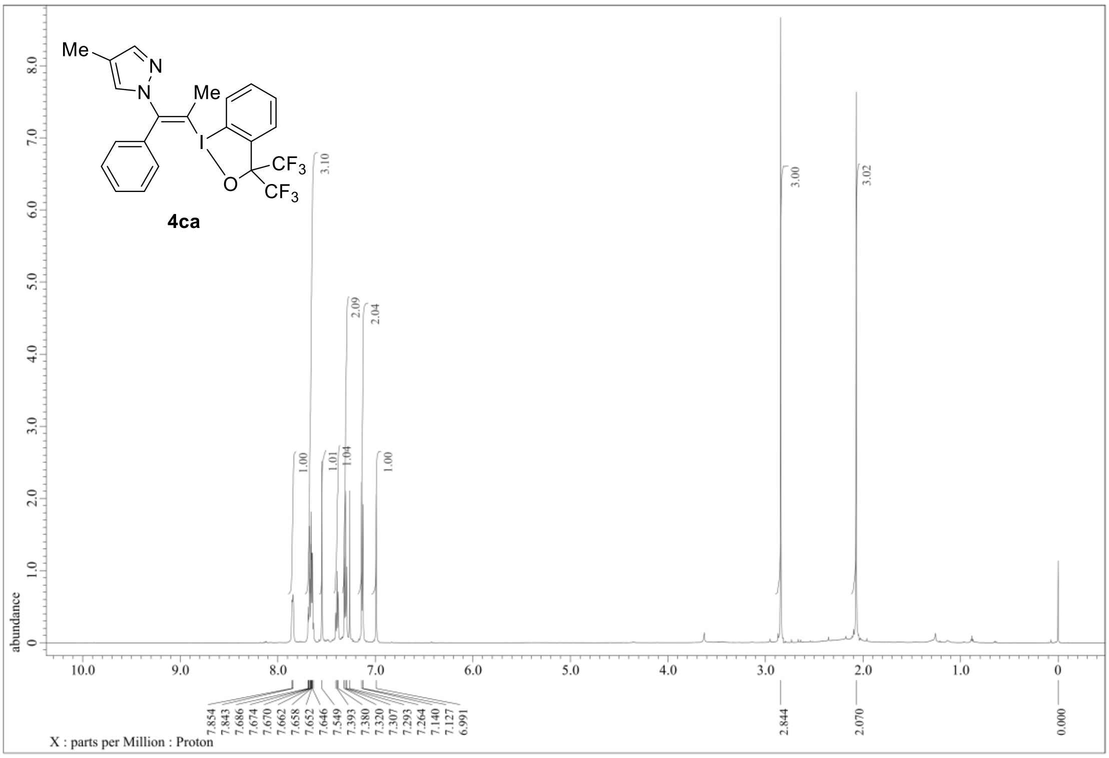

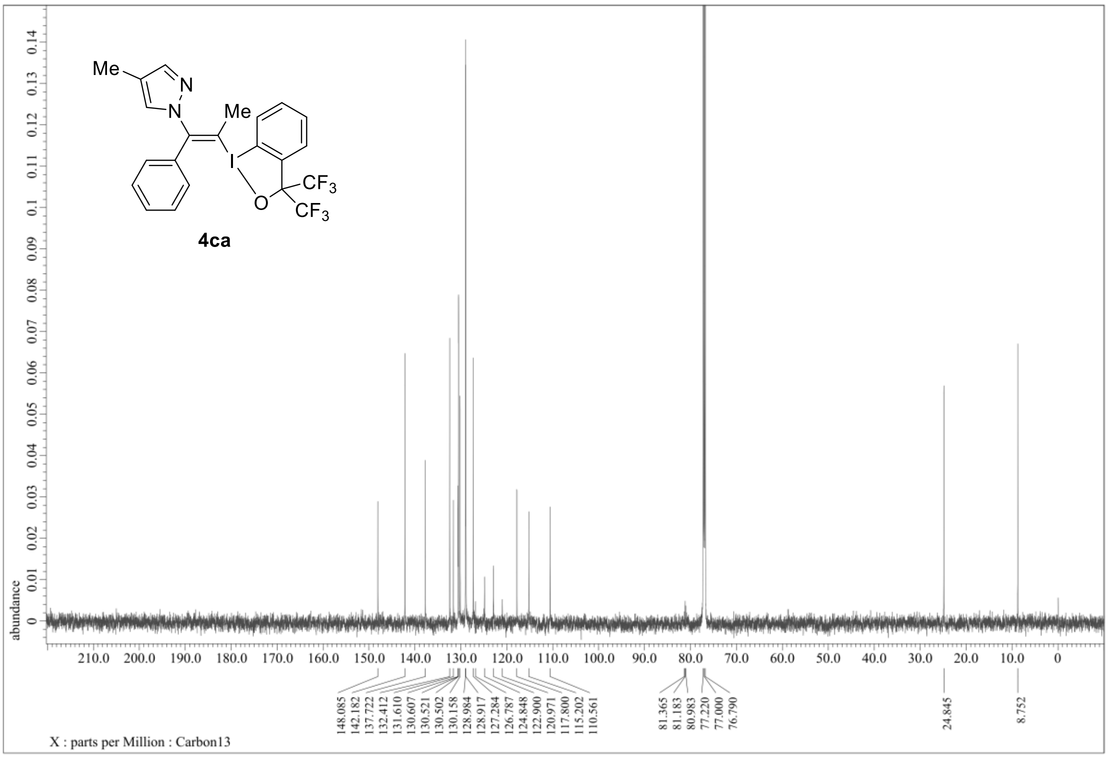

$^{19}F\{^{1}H\}$ NMR (565 MHz, $CDCl_3$) spectrum of **4ca**

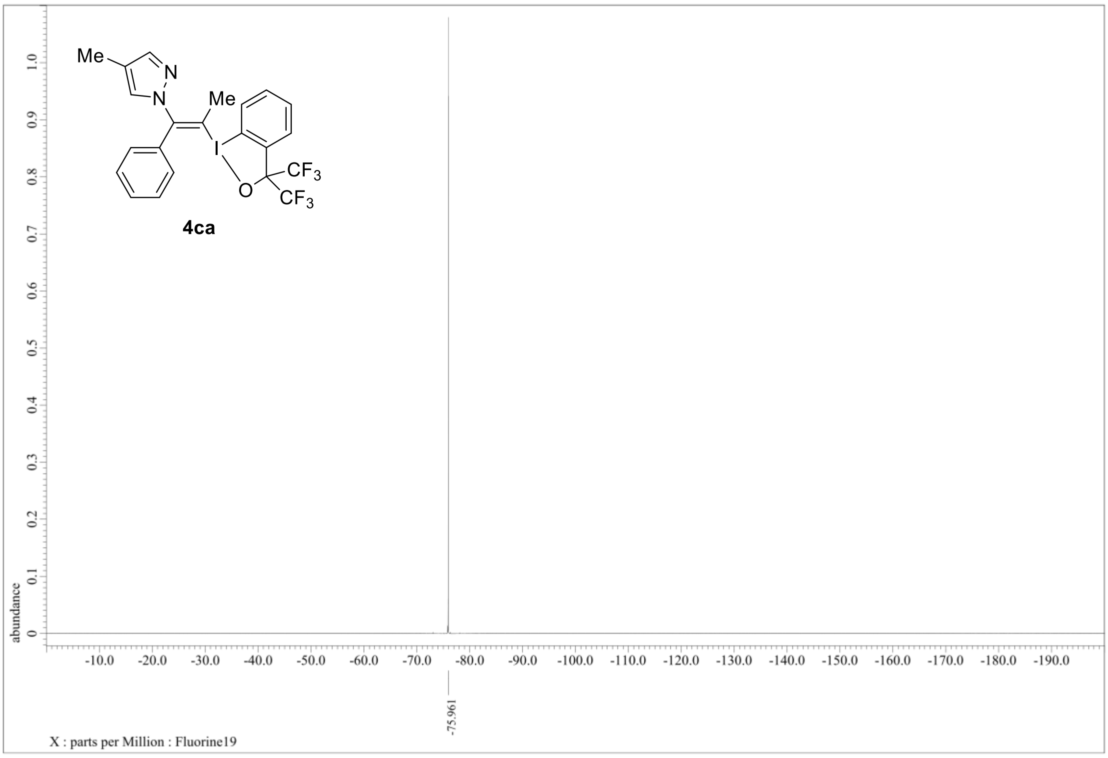

^{1}H NMR (600 MHz, $CDCl_3$) and ^{13}C{^{1}H} NMR (151 MHz, $CDCl_3$) spectra of **4da**

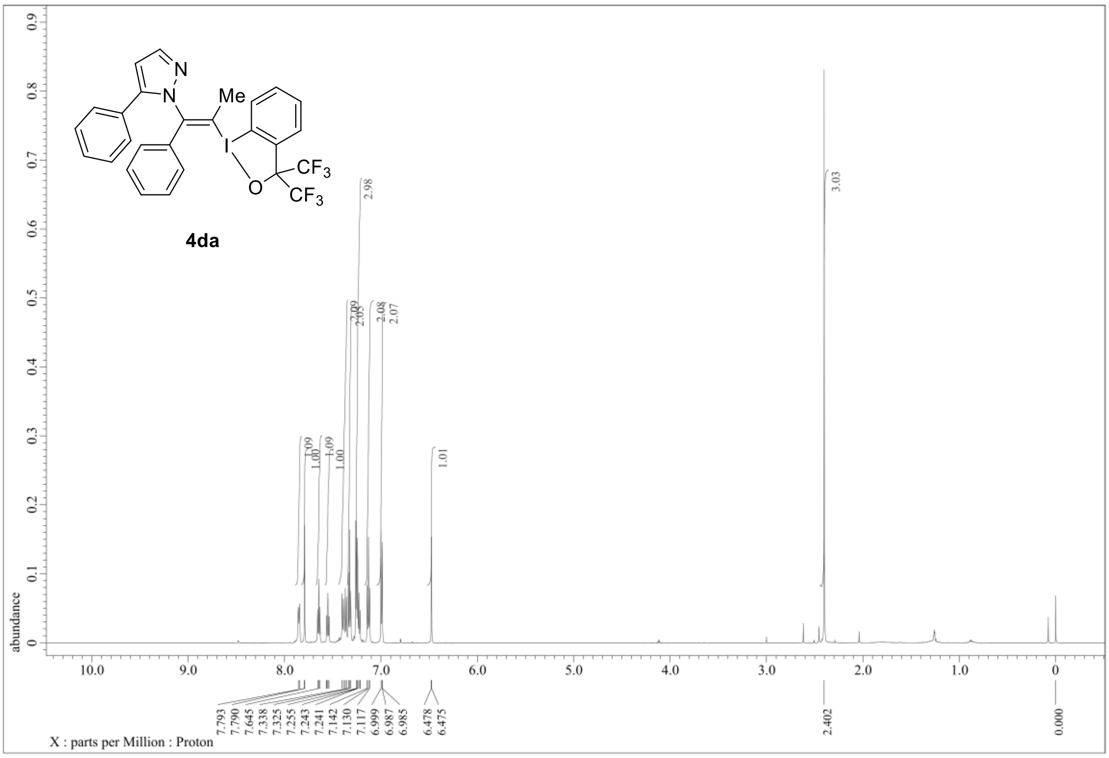

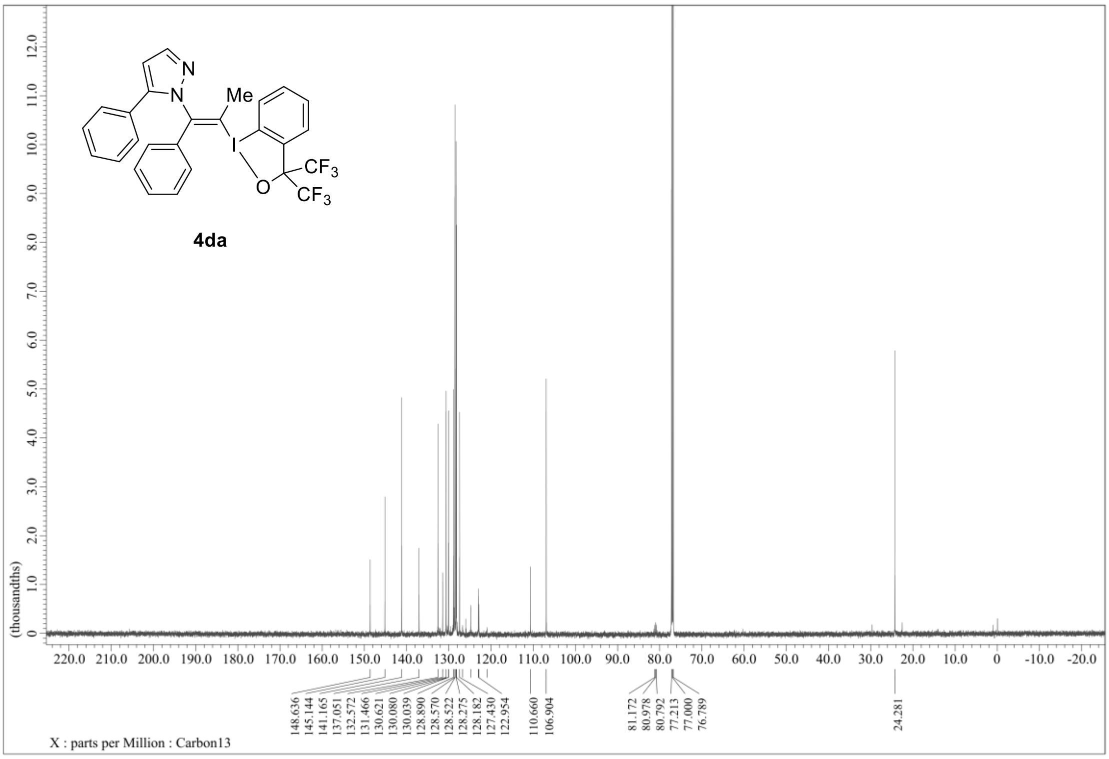

$^{19}F\{^{1}H\}$ NMR (565 MHz, $CDCl_3$) spectrum of **4da**

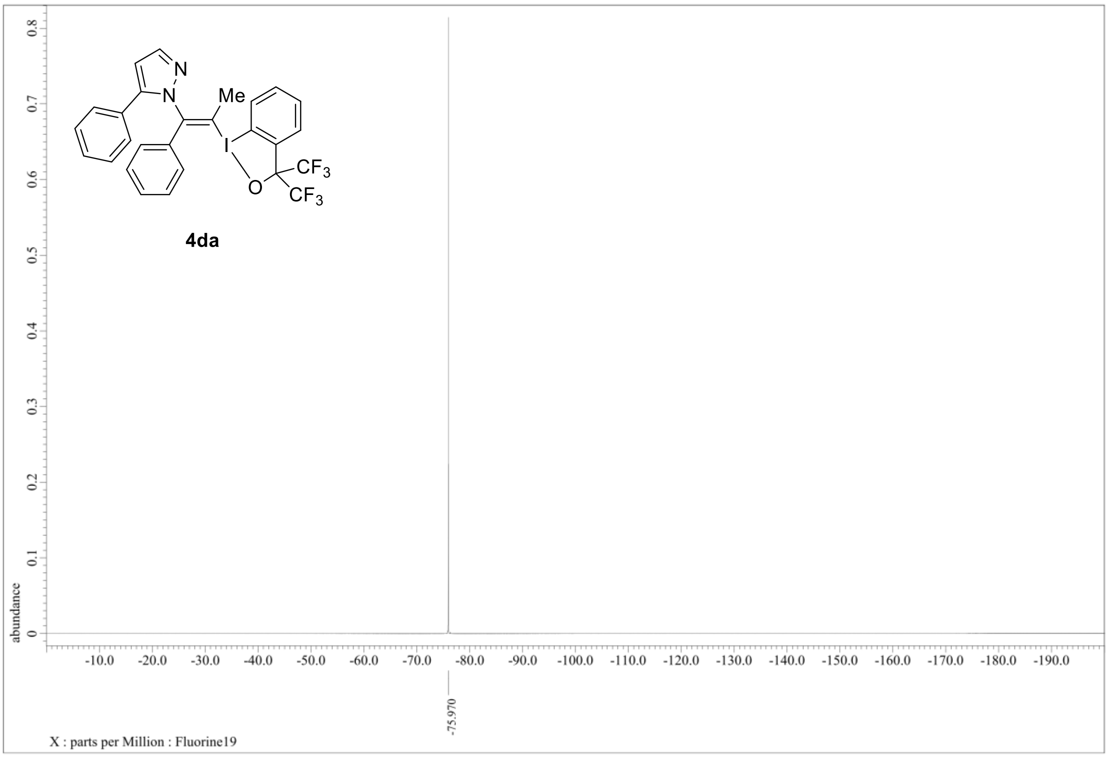

^{1}H NMR (600 MHz, $CDCl_3$) and ^{13}C{^{1}H} NMR (151 MHz, $CDCl_3$) spectra of **4da’**

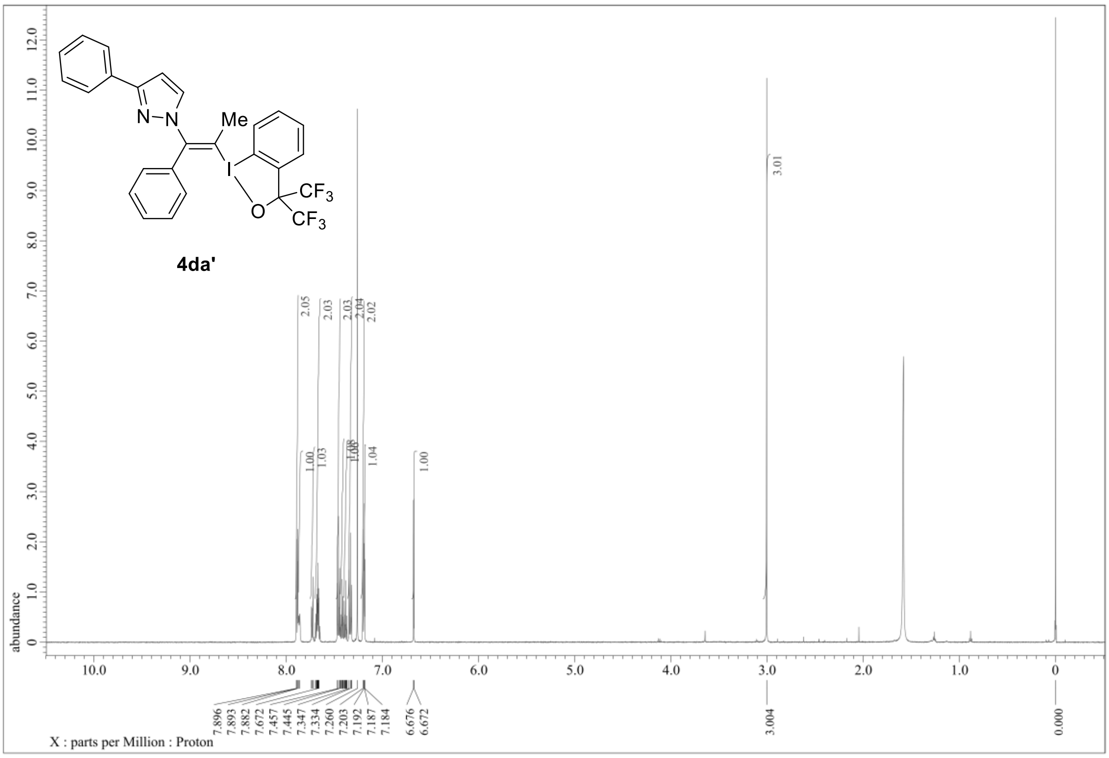

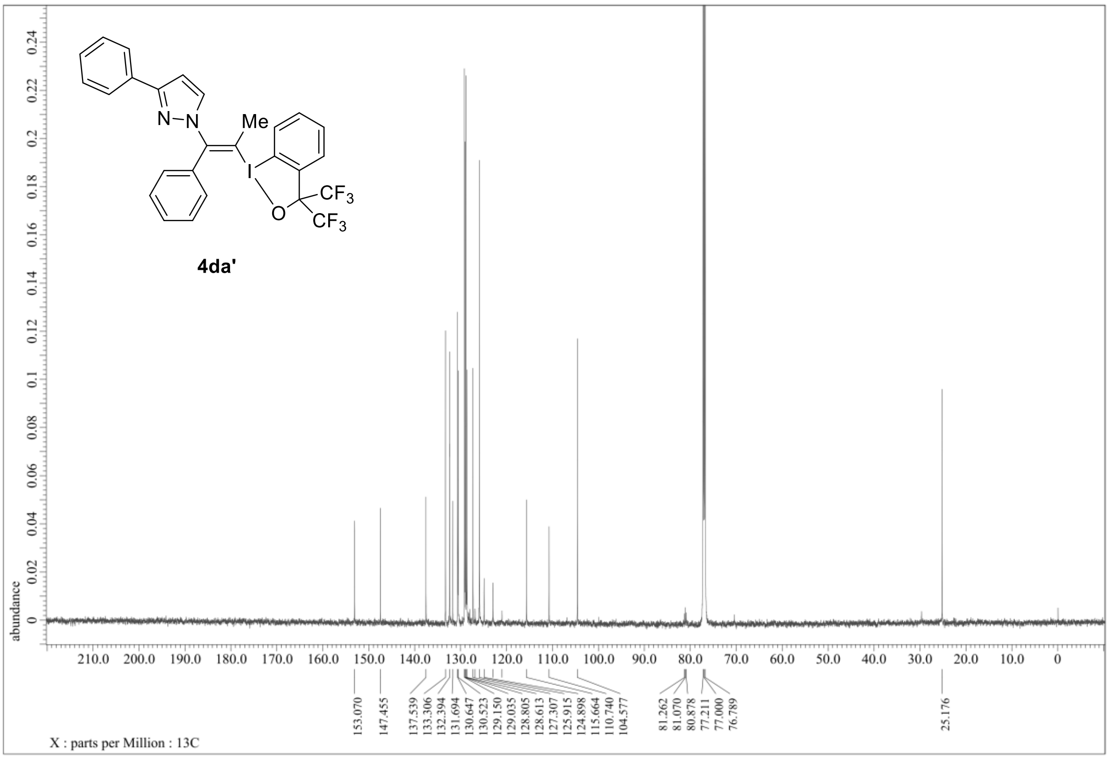

$^{19}F\{^{1}H\}$ NMR (565 MHz, $CDCl_3$) spectrum of **4da’**

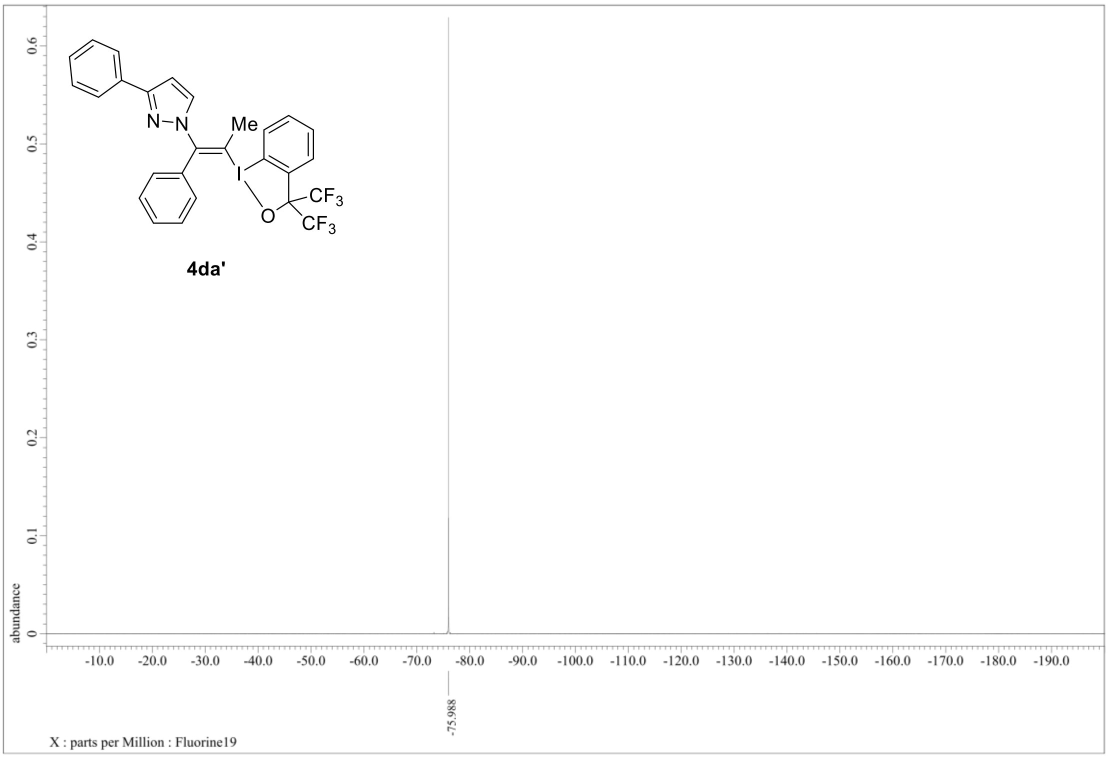

^{1}H NMR (600 MHz, $CDCl_3$) and $^{13}C\{^{1}H\}$ NMR (151 MHz, $CDCl_3$) spectra of **4ea**

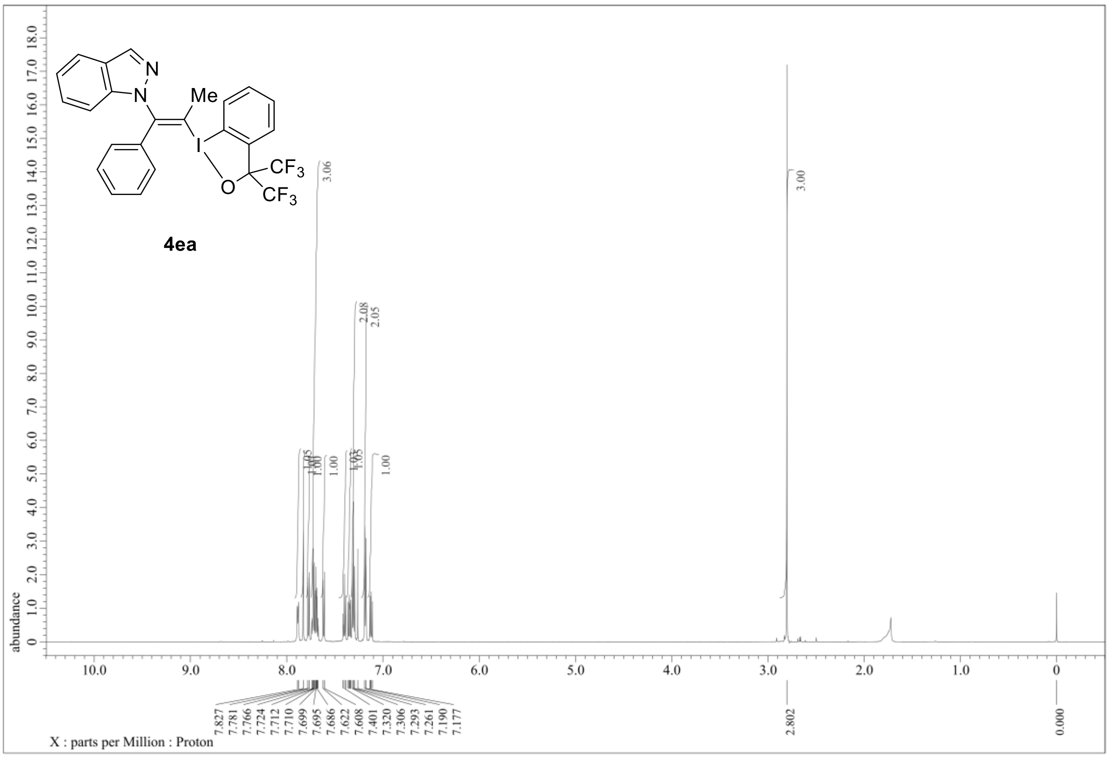

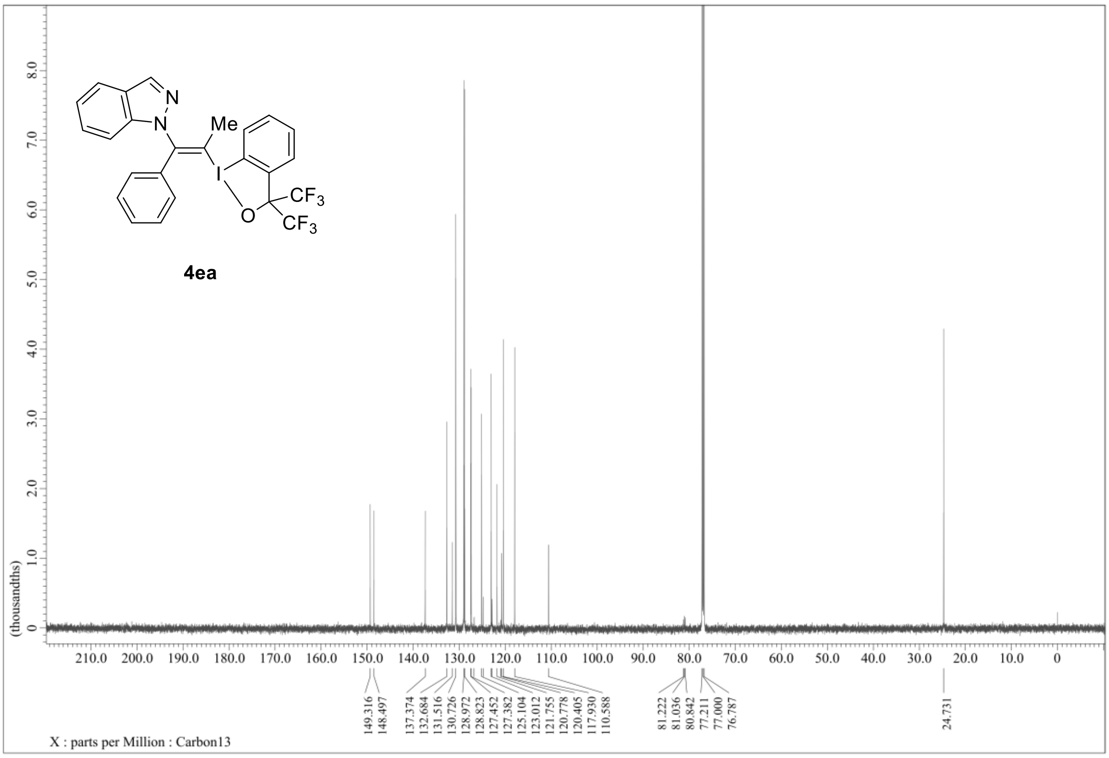

$^{19}F\{^{1}H\}$ NMR (565 MHz, $CDCl_3$) spectrum of **4ea**

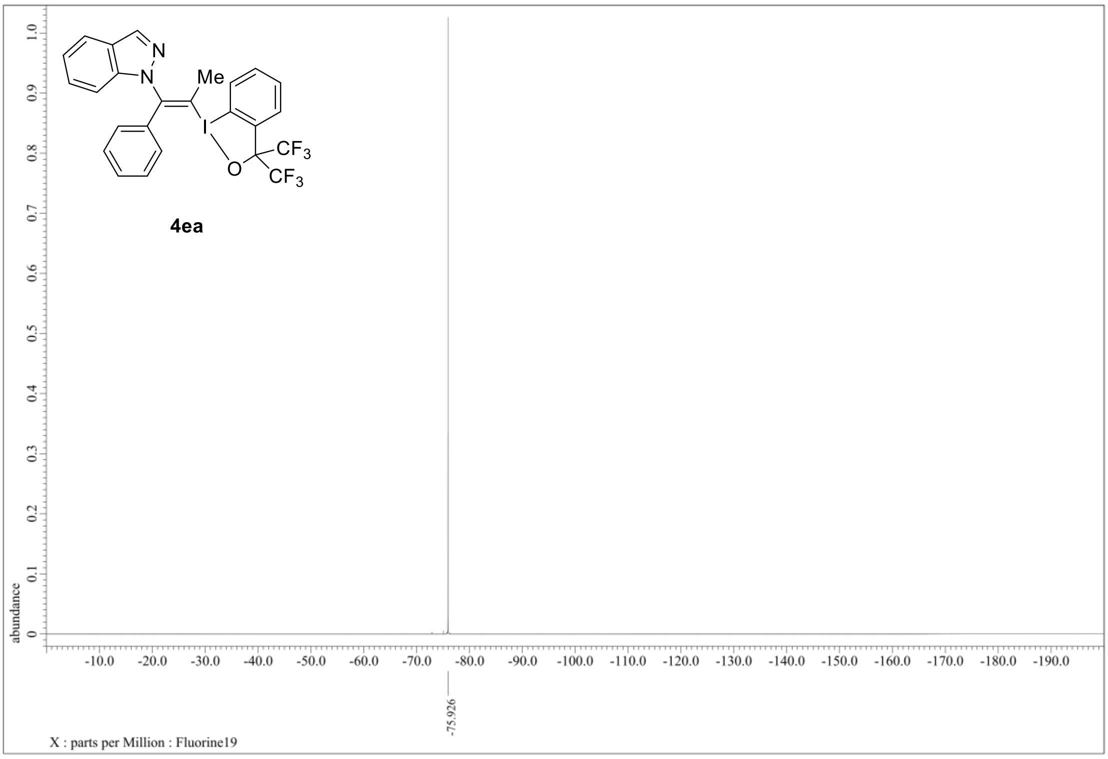

^{1}H NMR (600 MHz, $CDCl_3$) and $^{13}C\{^{1}H\}$ NMR (151 MHz, $CDCl_3$) spectra of **4fa**

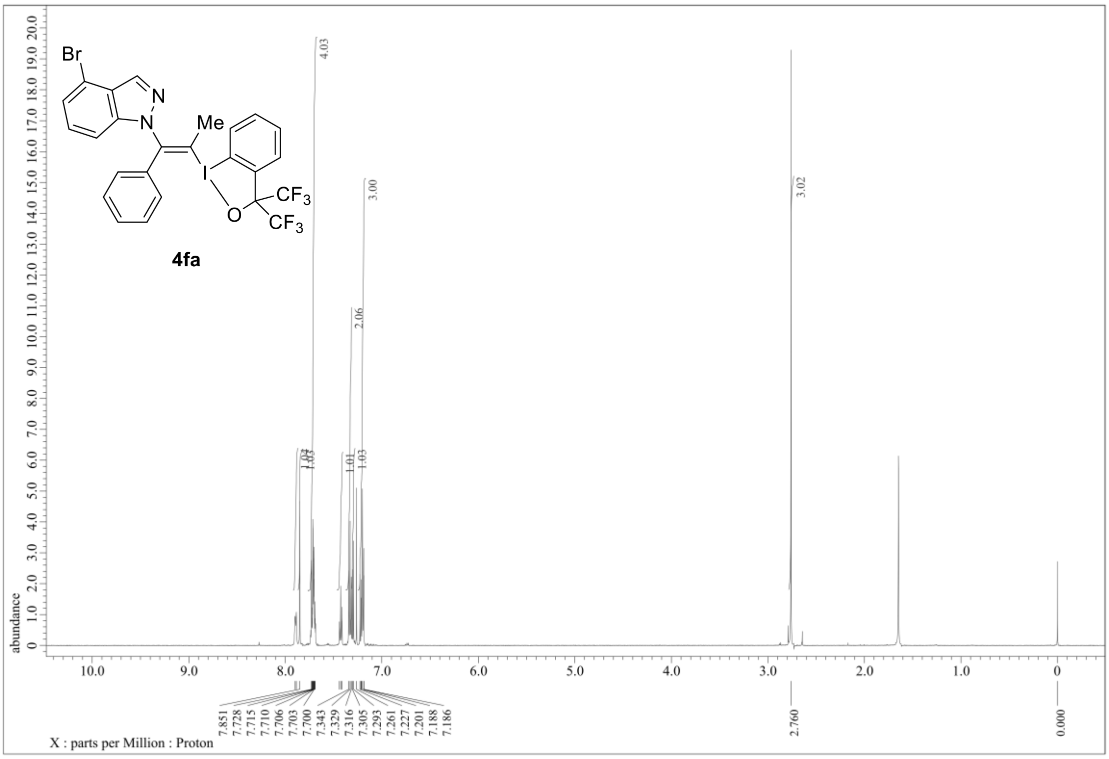

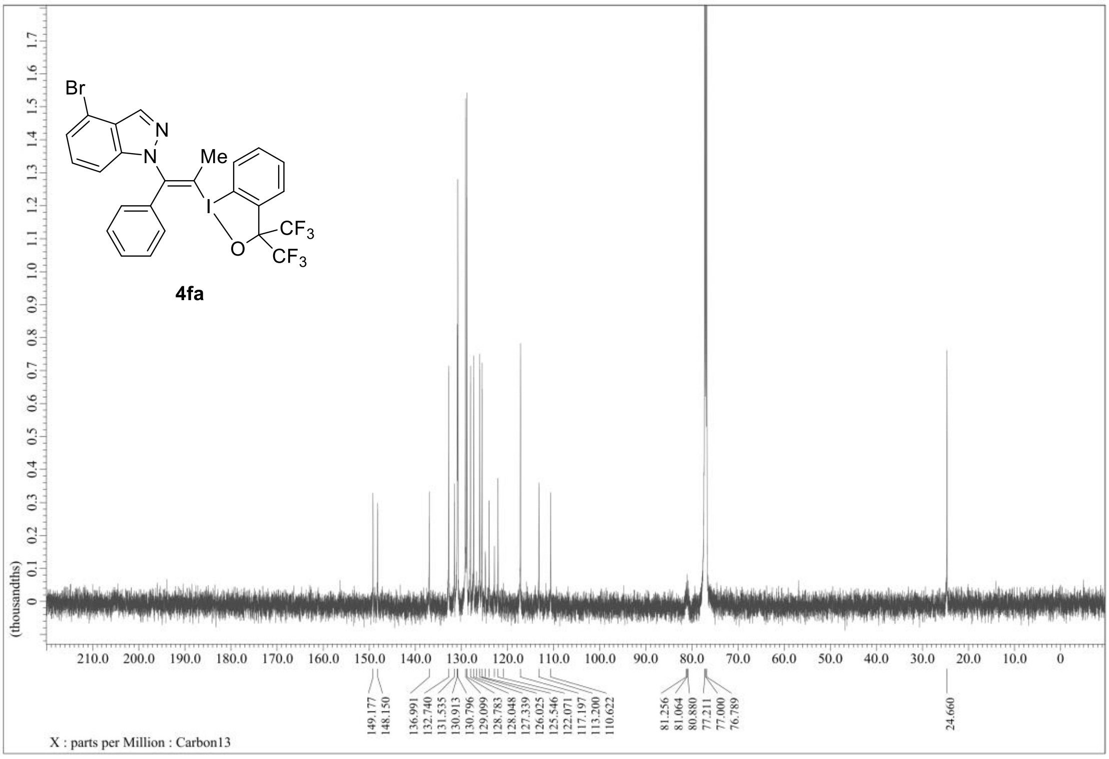

$^{19}F\{^1H\}$ NMR (565 MHz, $CDCl_3$) spectrum of **4fa**

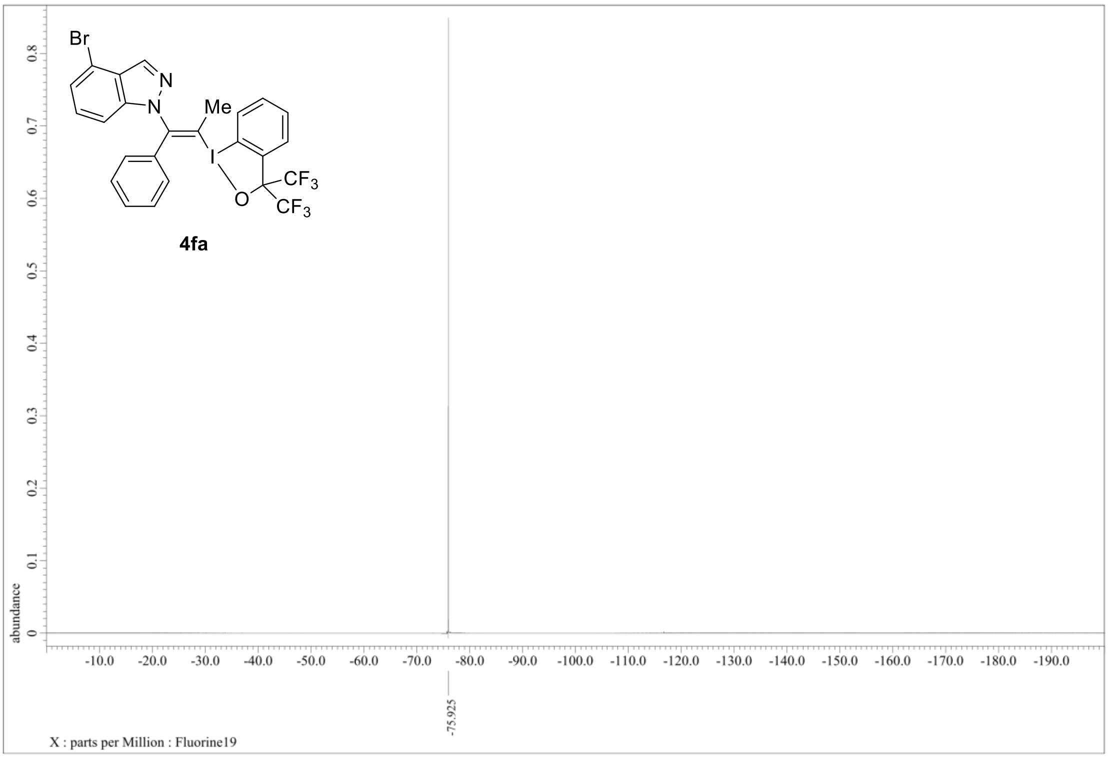

^{1}H NMR (600 MHz, $CDCl_3$) and $^{13}C\{^1H\}$ NMR (151 MHz, $CDCl_3$) spectra of **4ga**

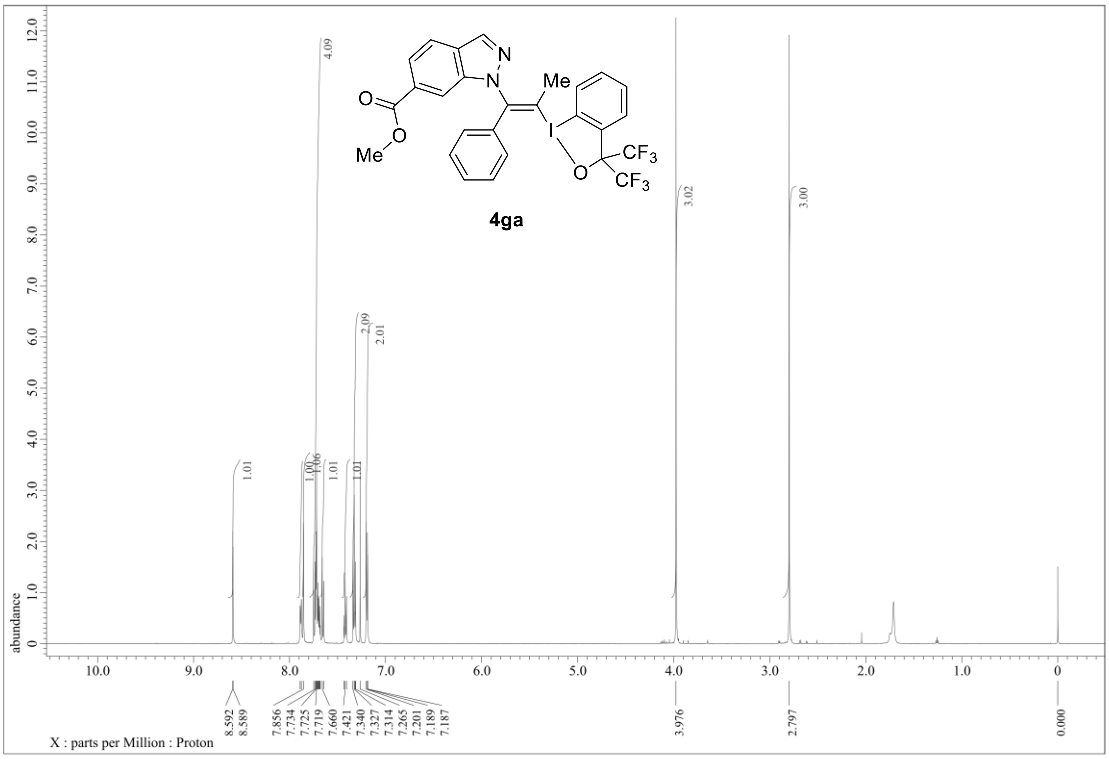

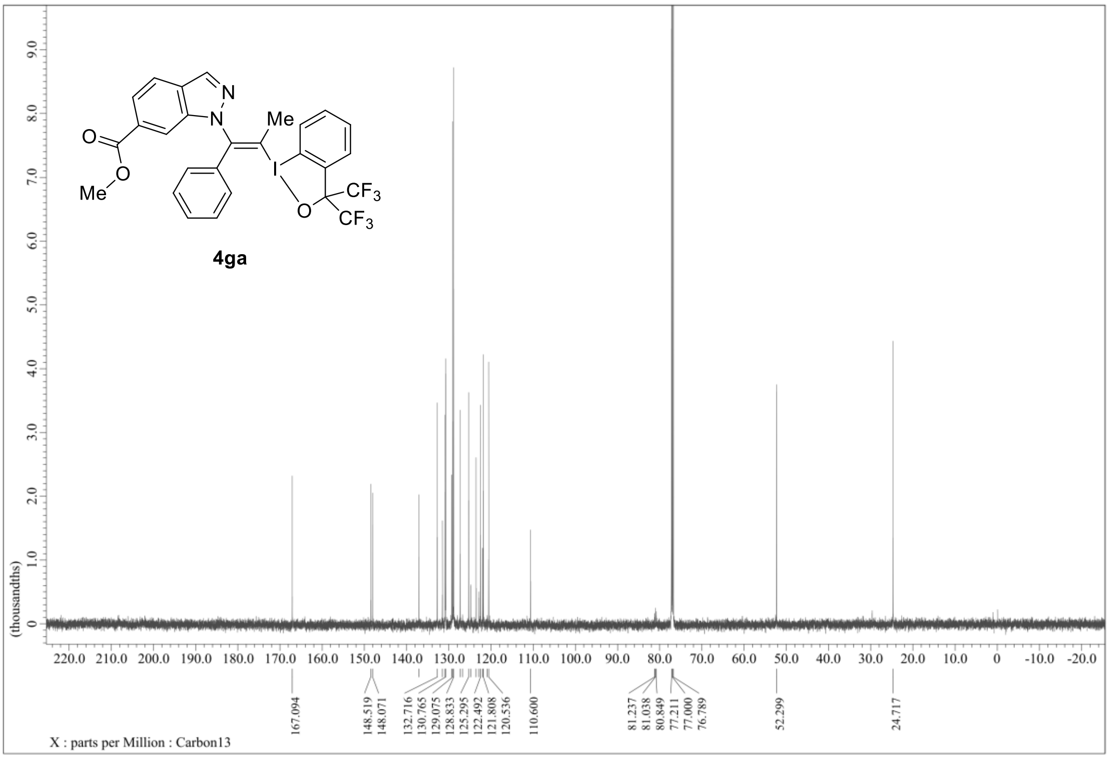

$^{19}F\{^{1}H\}$ NMR (565 MHz, $CDCl_3$) spectrum of **4ga**

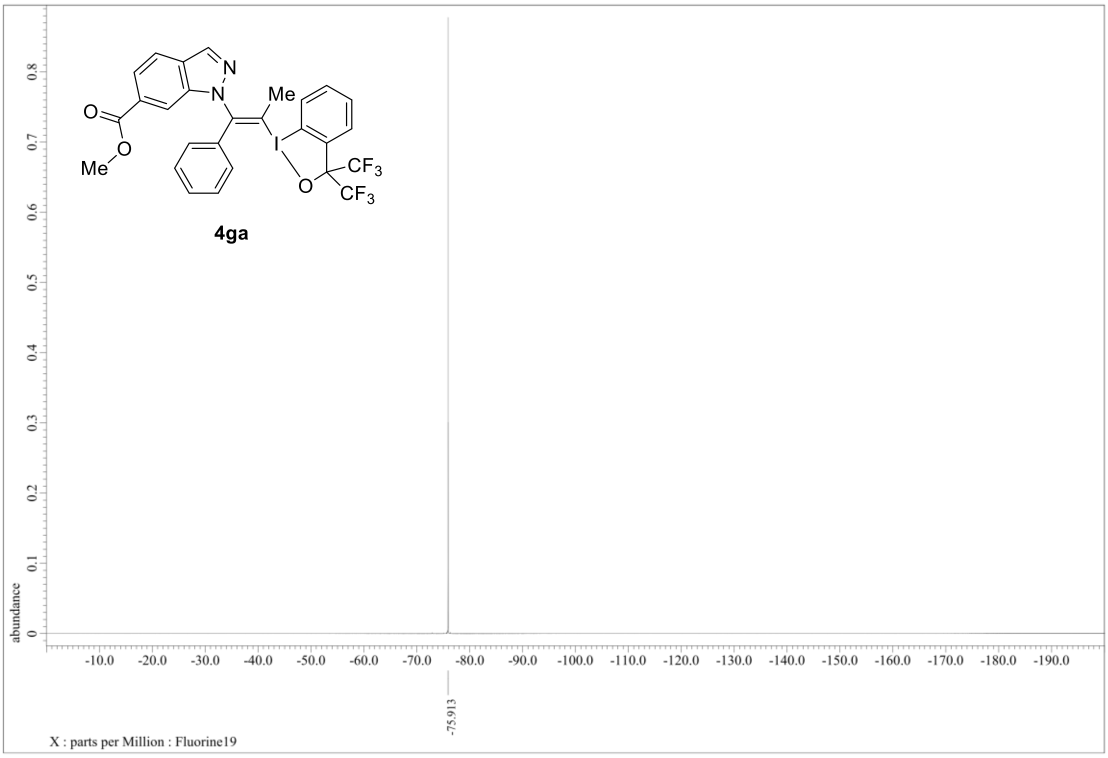

^{1}H NMR (600 MHz, $CDCl_3$) and $^{13}C\{^{1}H\}$ NMR (151 MHz, $CDCl_3$) spectra of **4ha**

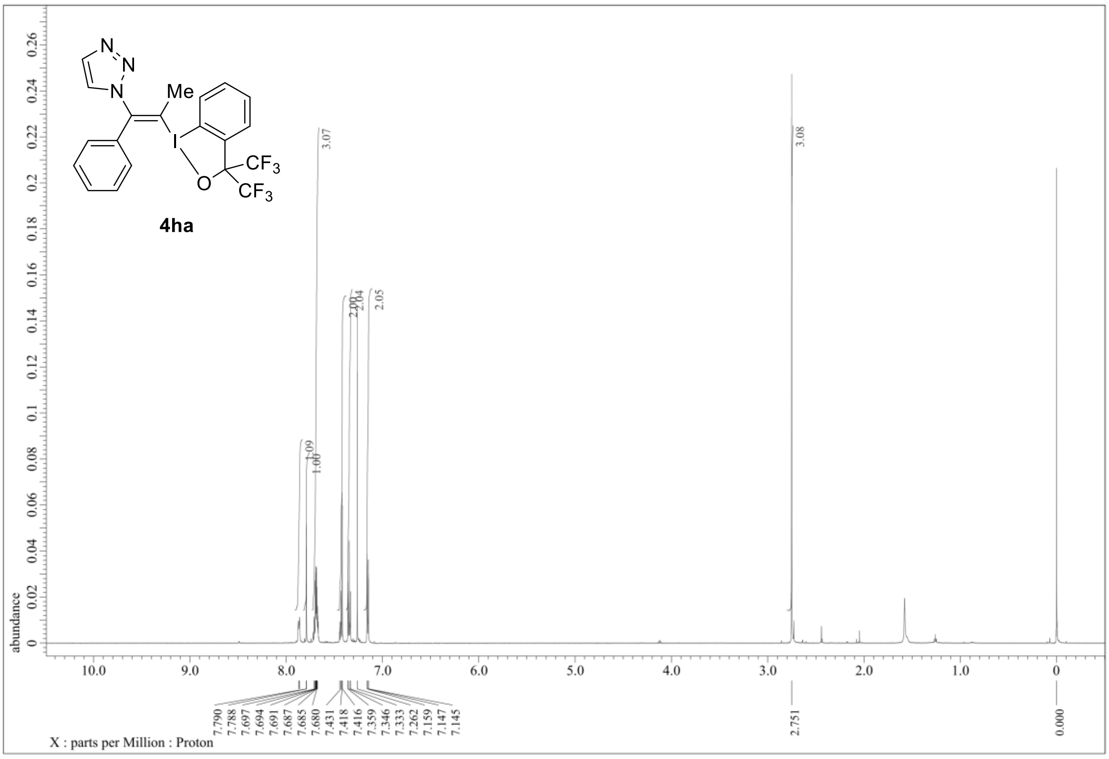

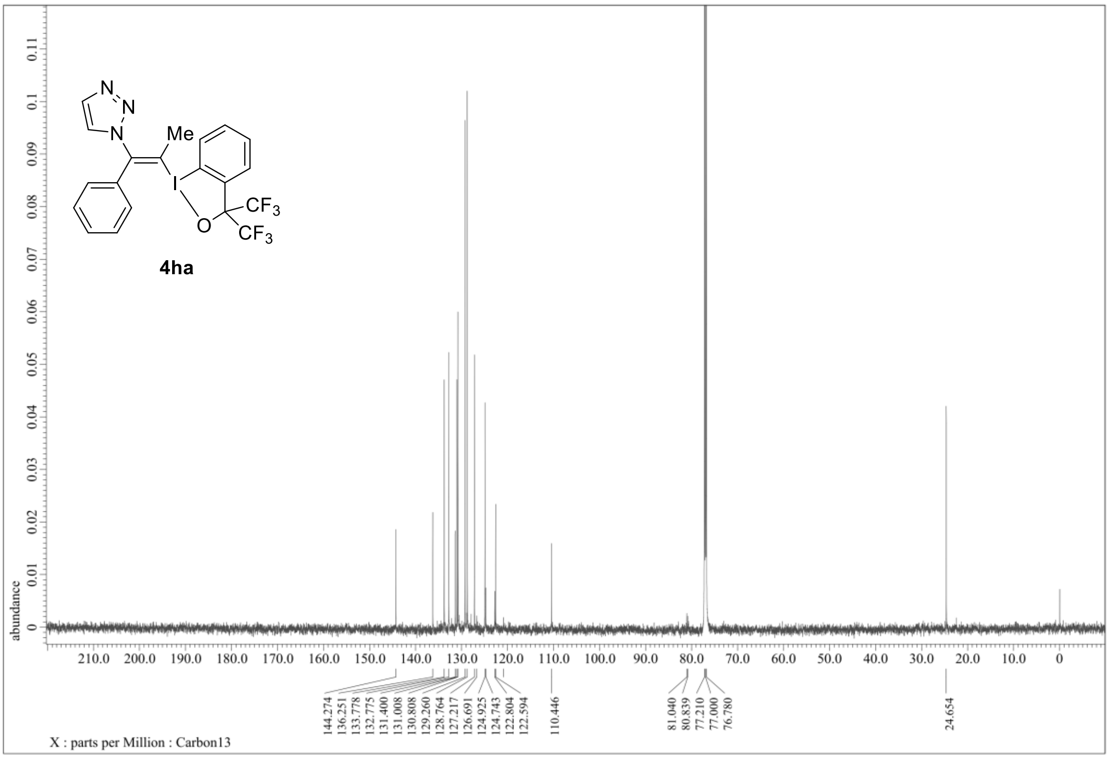

$^{19}F\{^1H\}$ NMR (565 MHz, $CDCl_3$) spectrum of **4ha**

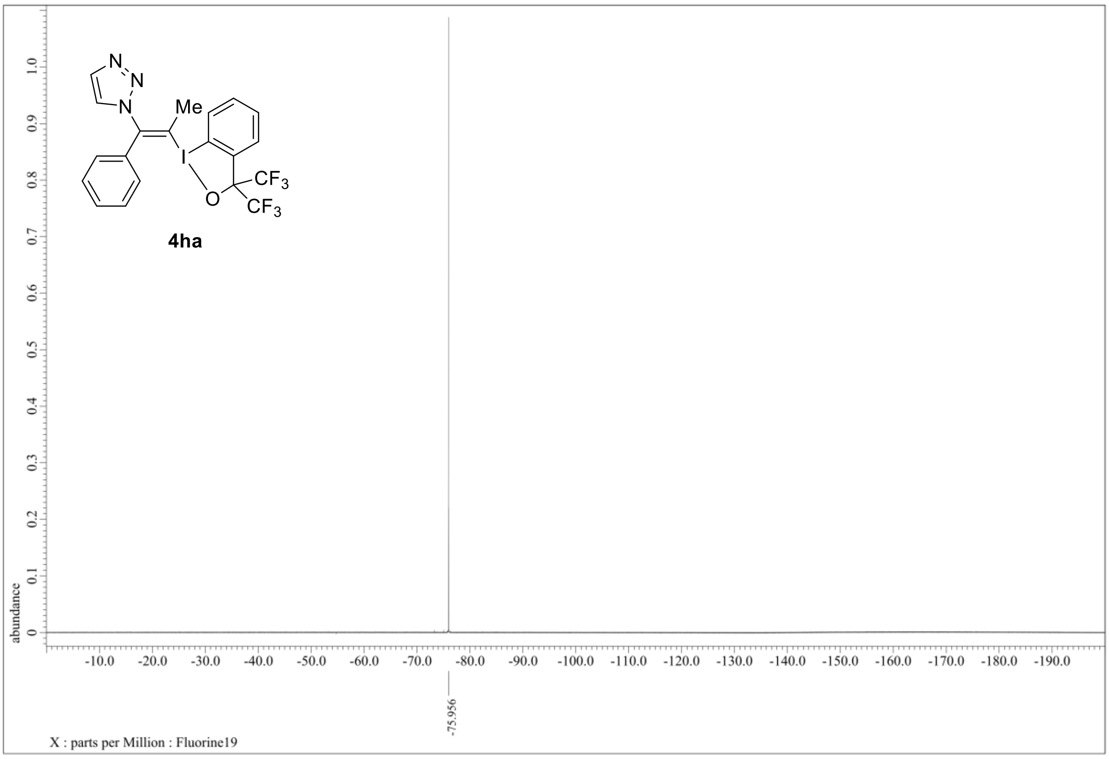

^{1}H NMR (600 MHz, $CDCl_3$) and $^{13}C\{^1H\}$ NMR (151 MHz, $CDCl_3$) spectra of **4ia**

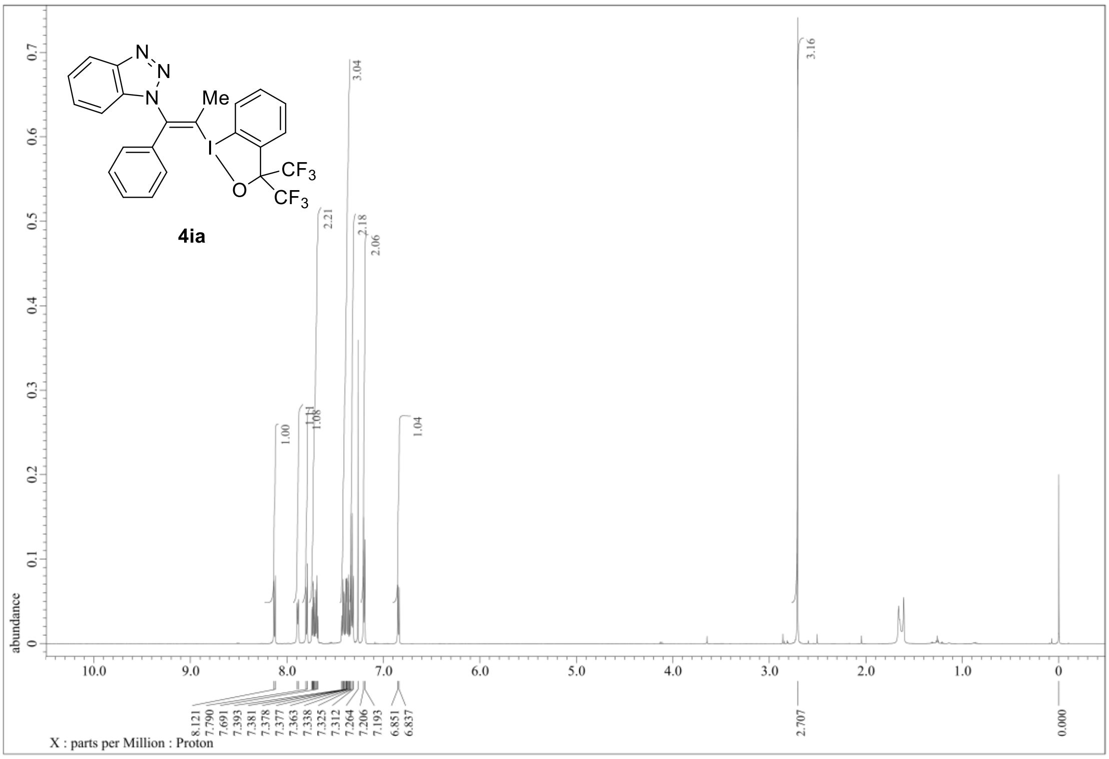

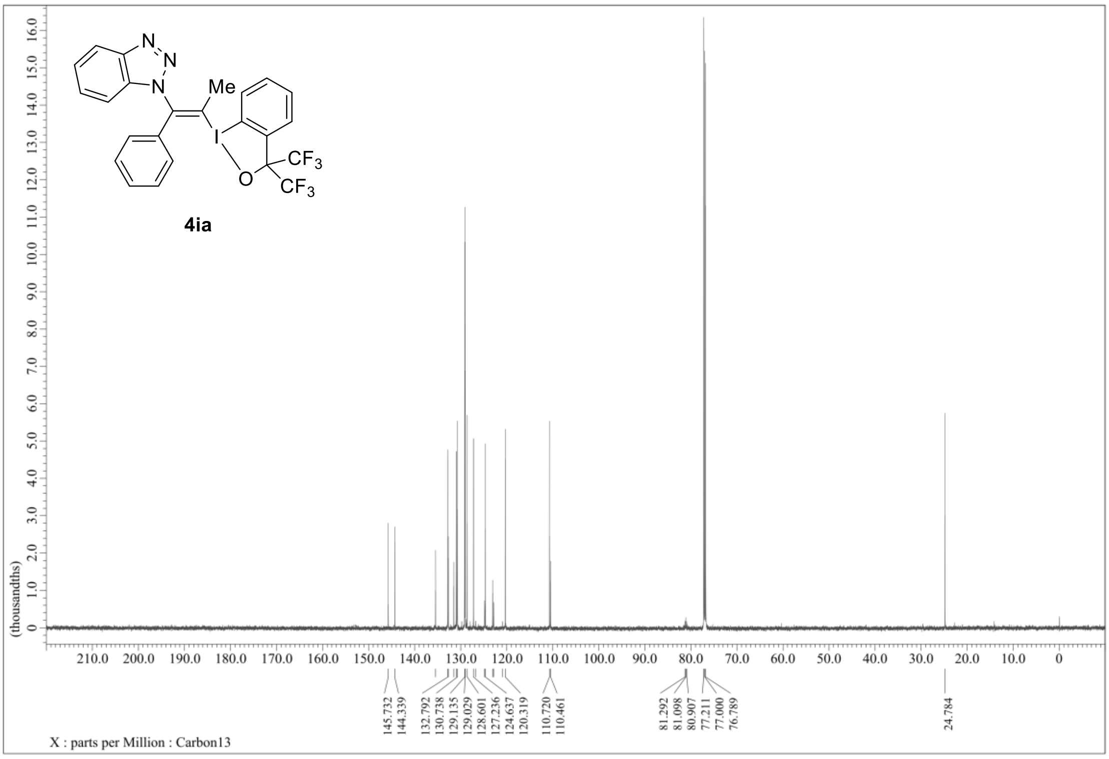

$^{19}F\{^{1}H\}$ NMR (565 MHz, $CDCl_3$) spectrum of **4ia**

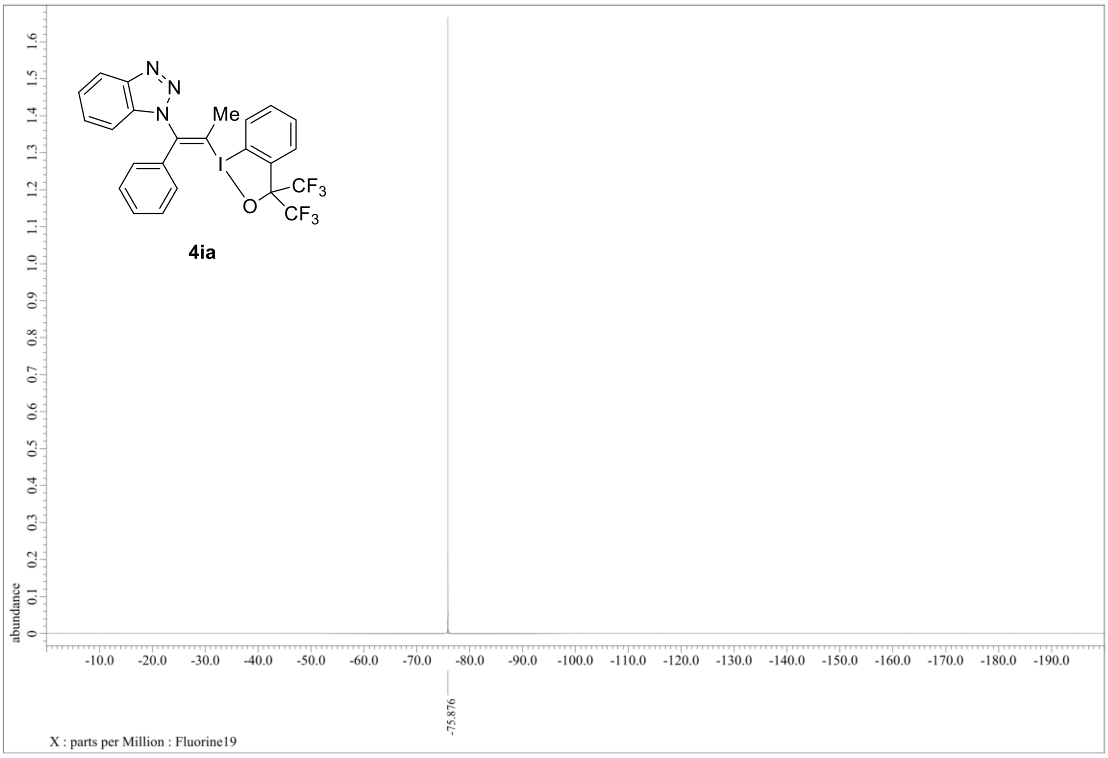

^1H NMR (600 MHz, $CDCl_3$) and $^{13}C\{^1H\}$ NMR (151 MHz, $CDCl_3$) spectra of **4ja**

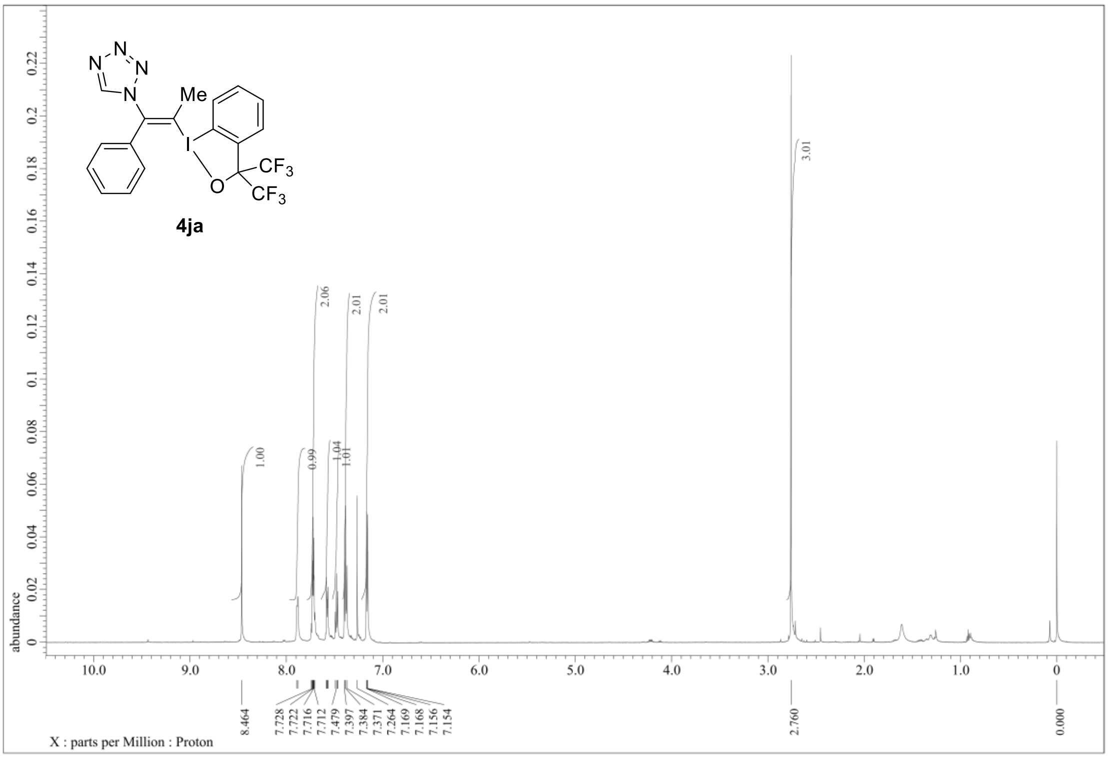

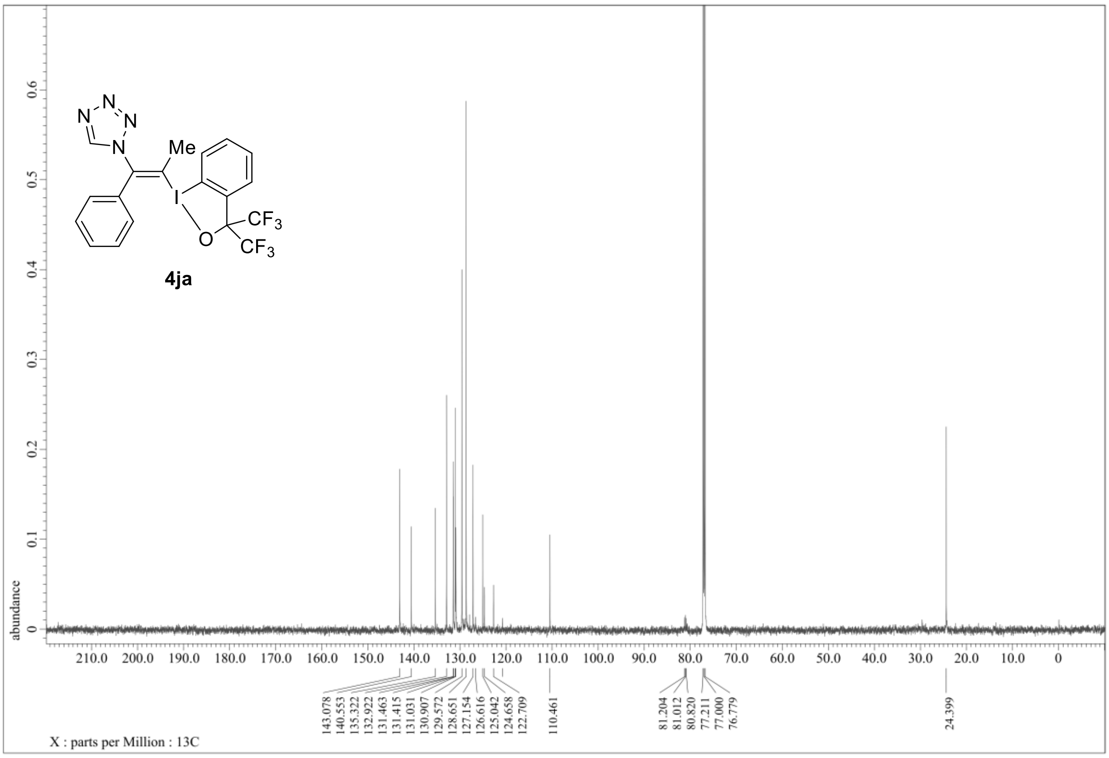

$^{19}F\{^1H\}$ NMR (565 MHz, $CDCl_3$) spectrum of **4ja**

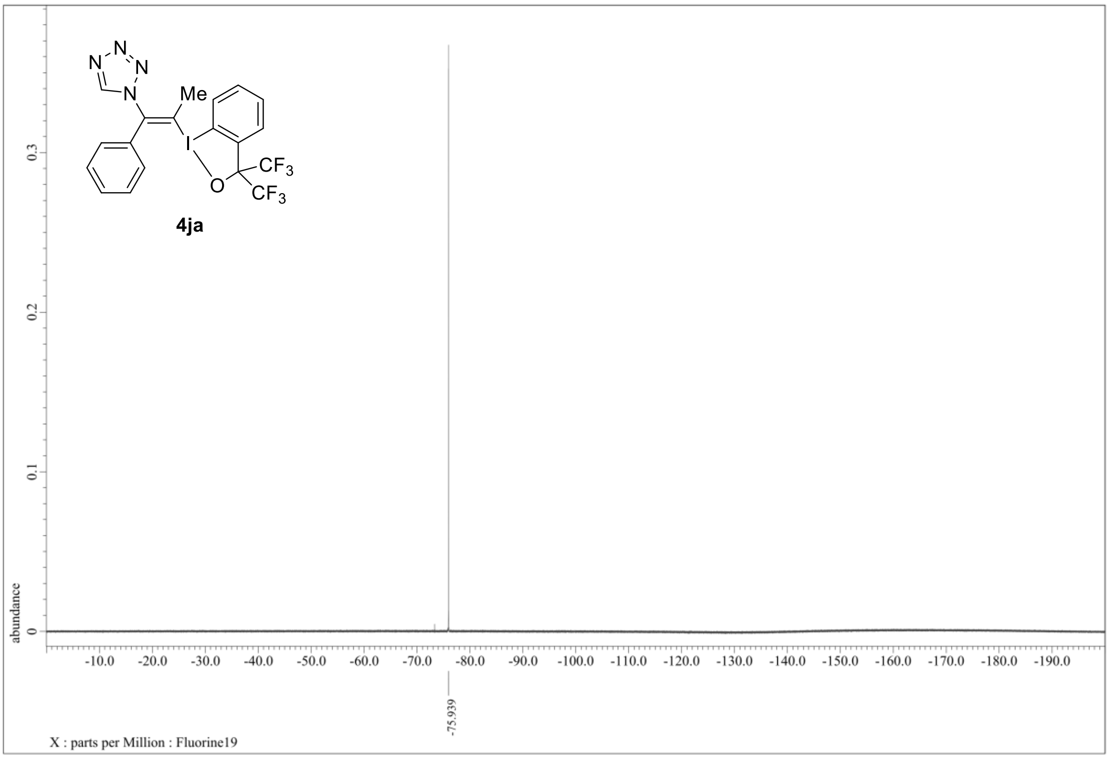

^{1}H NMR (600 MHz, $CDCl_3$) and $^{13}C\{^{1}H\}$ NMR (151 MHz, $CDCl_3$) spectra of **4ja'**

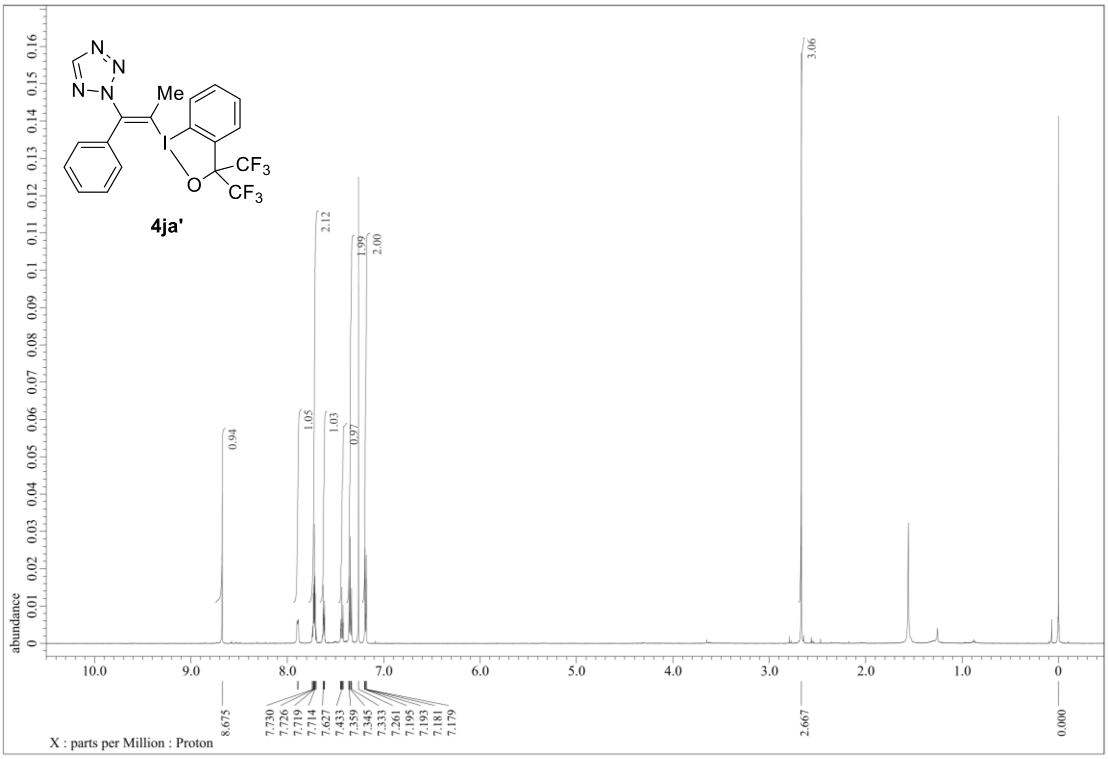

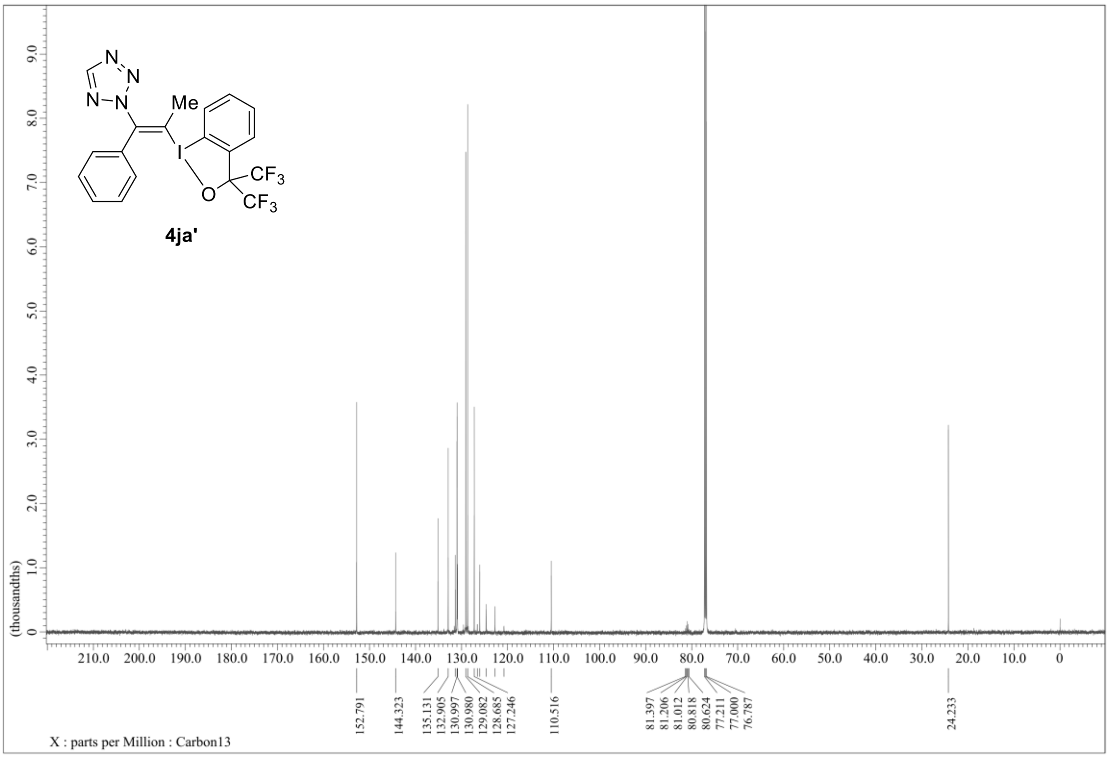

$^{19}F\{^{1}H\}$ NMR (565 MHz, $CDCl_3$) spectrum of **4ja’**

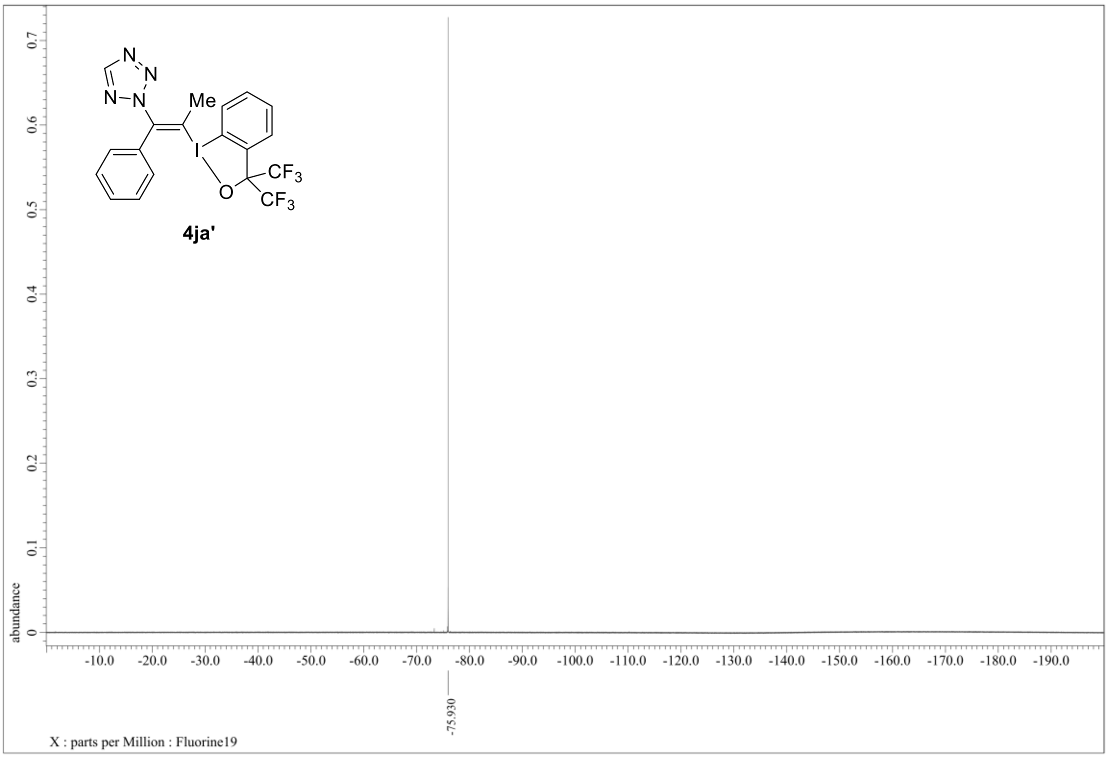

^{1}H NMR (600 MHz, $CDCl_3$) and $^{13}C\{^{1}H\}$ NMR (151 MHz, $CDCl_3$) spectra of **4ka**

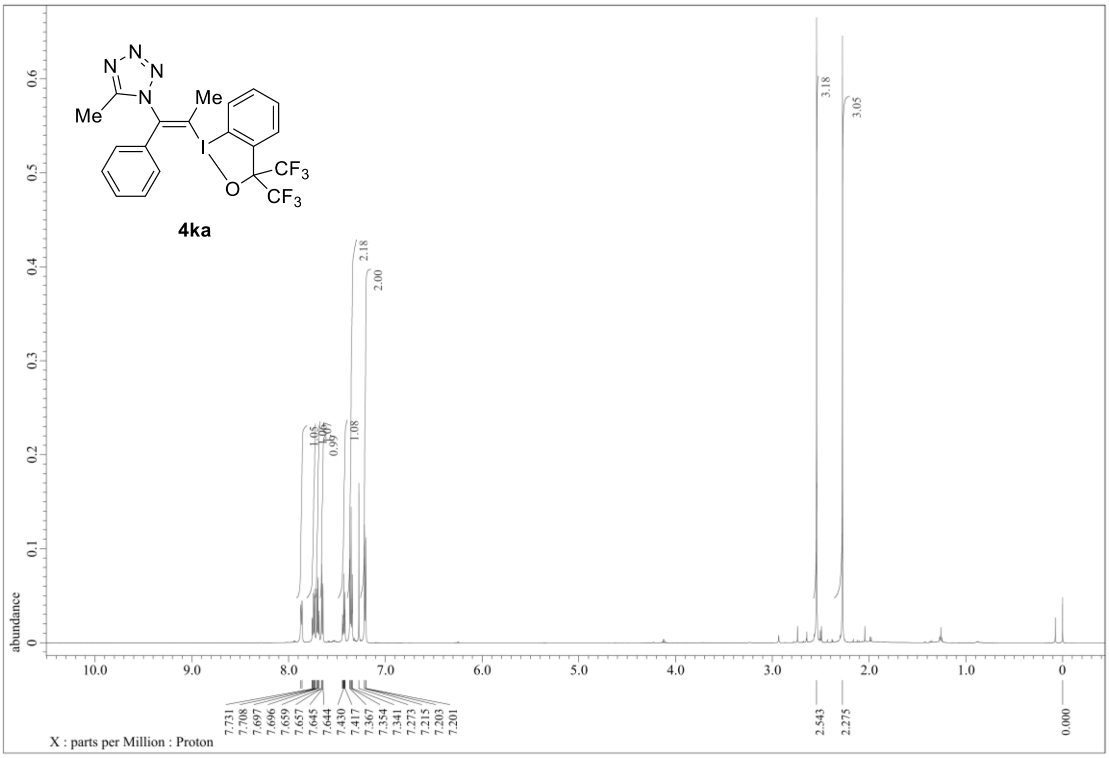

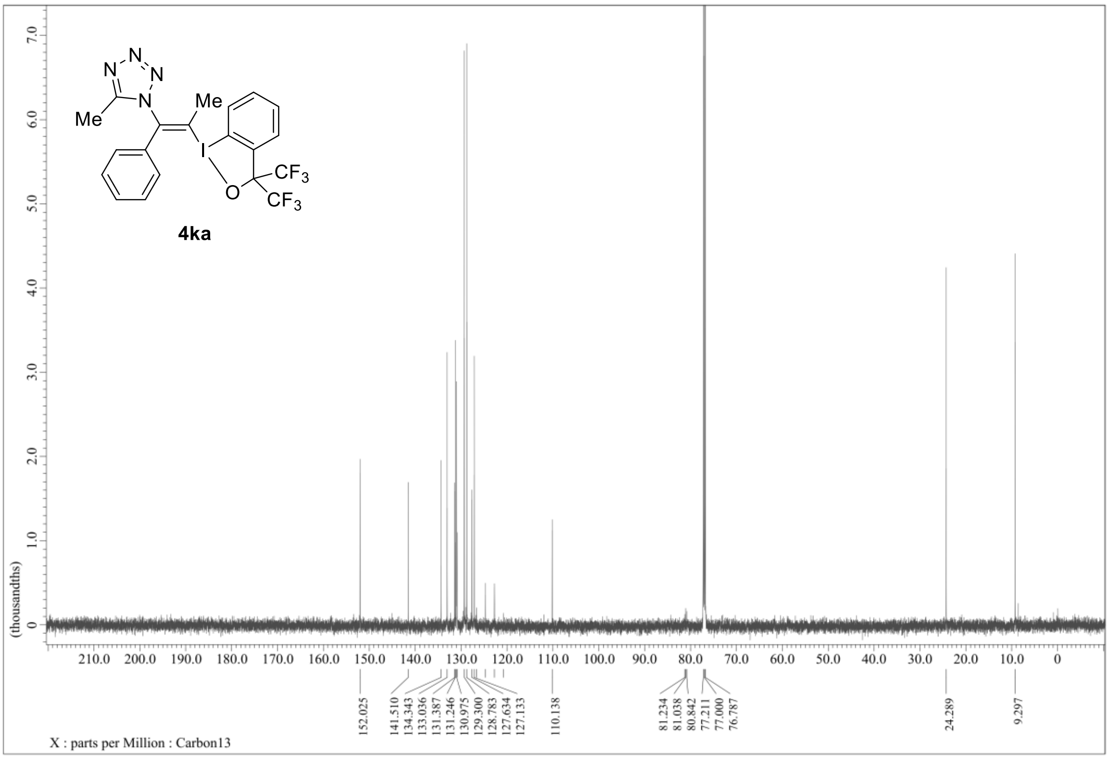

$^{19}F\{^{1}H\}$ NMR (565 MHz, $CDCl_3$) spectrum of **4ka**

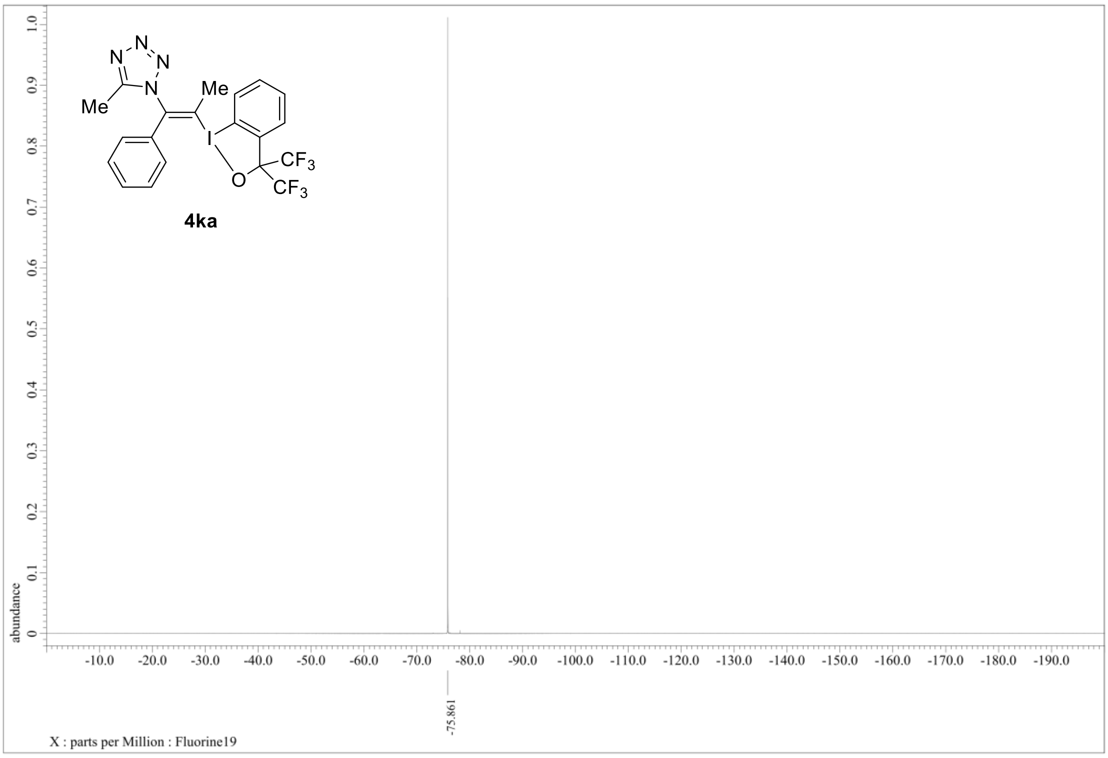

^{1}H NMR (600 MHz, $CDCl_3$) and ^{13}C{^{1}H} NMR (151 MHz, $CDCl_3$) spectra of **4ka'**

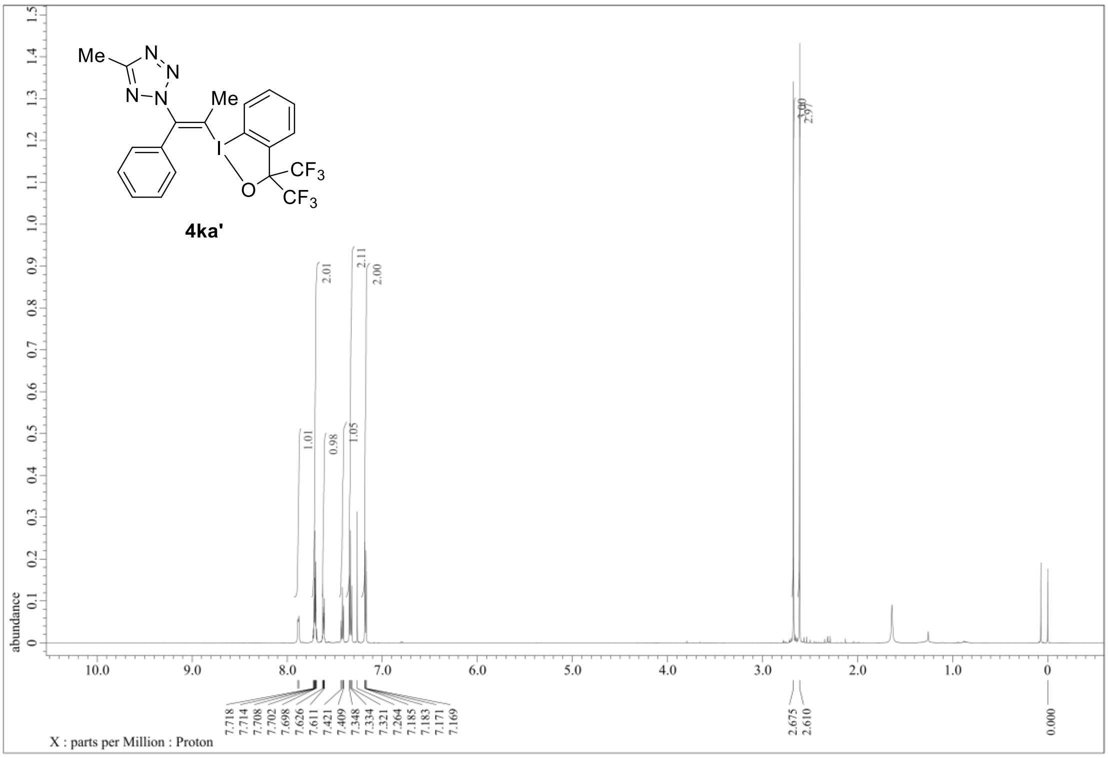

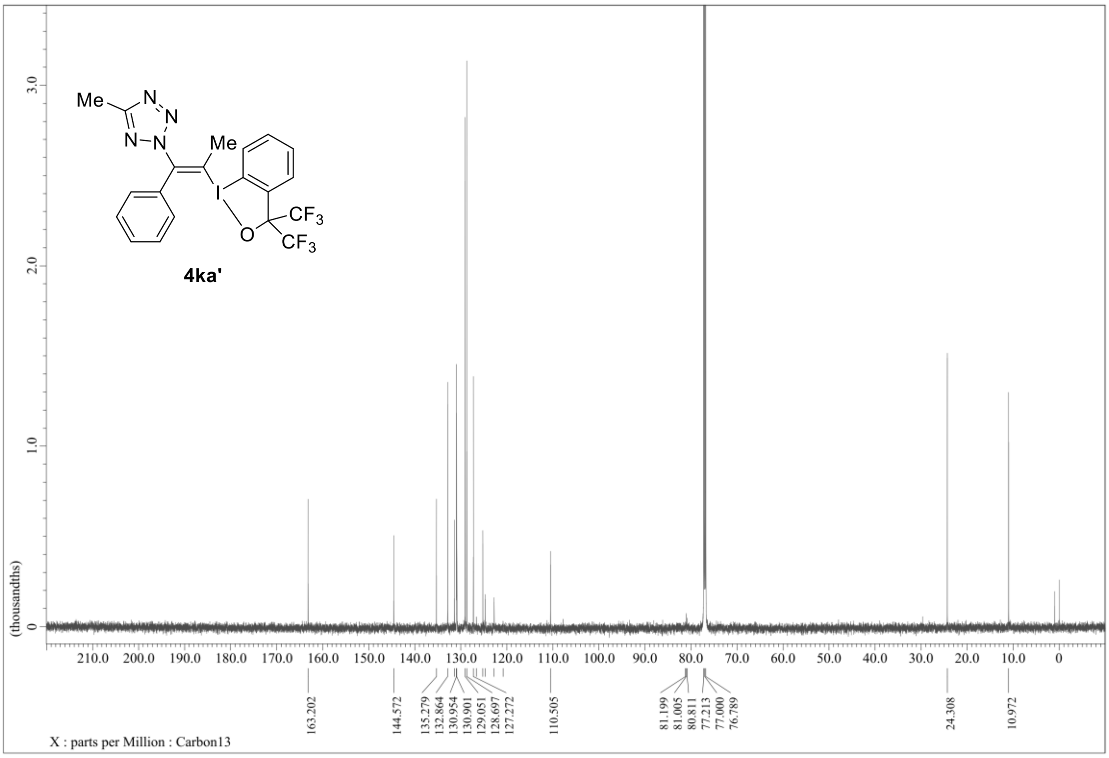

$^{19}F\{^{1}H\}$ NMR (565 MHz, $CDCl_3$) spectrum of **4ka’**

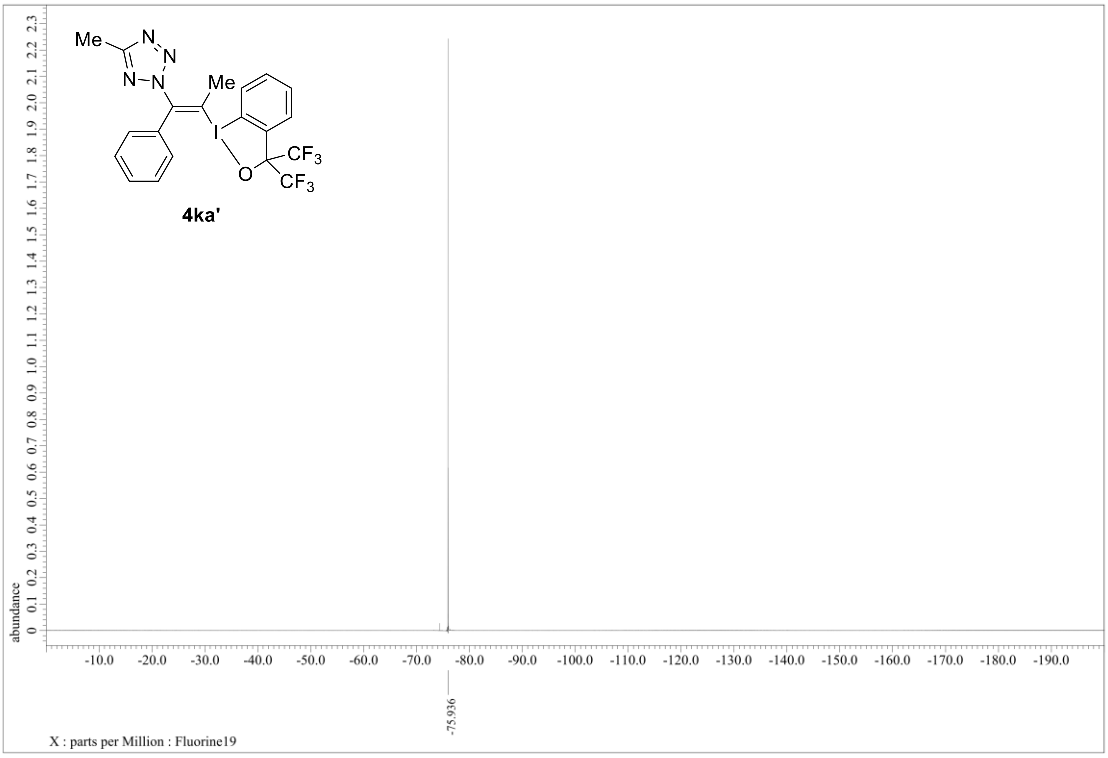

^{1}H NMR (600 MHz, $CDCl_3$) and ^{13}C{^{1}H} NMR (151 MHz, $CDCl_3$) spectra of **4ab**

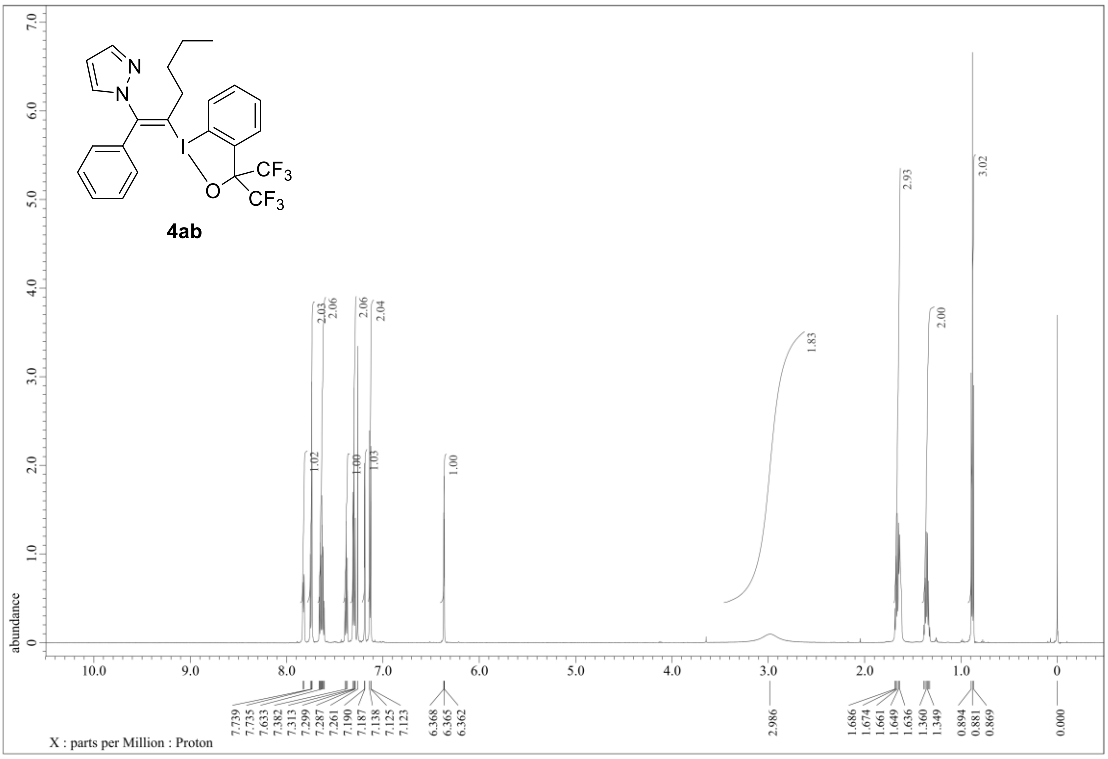

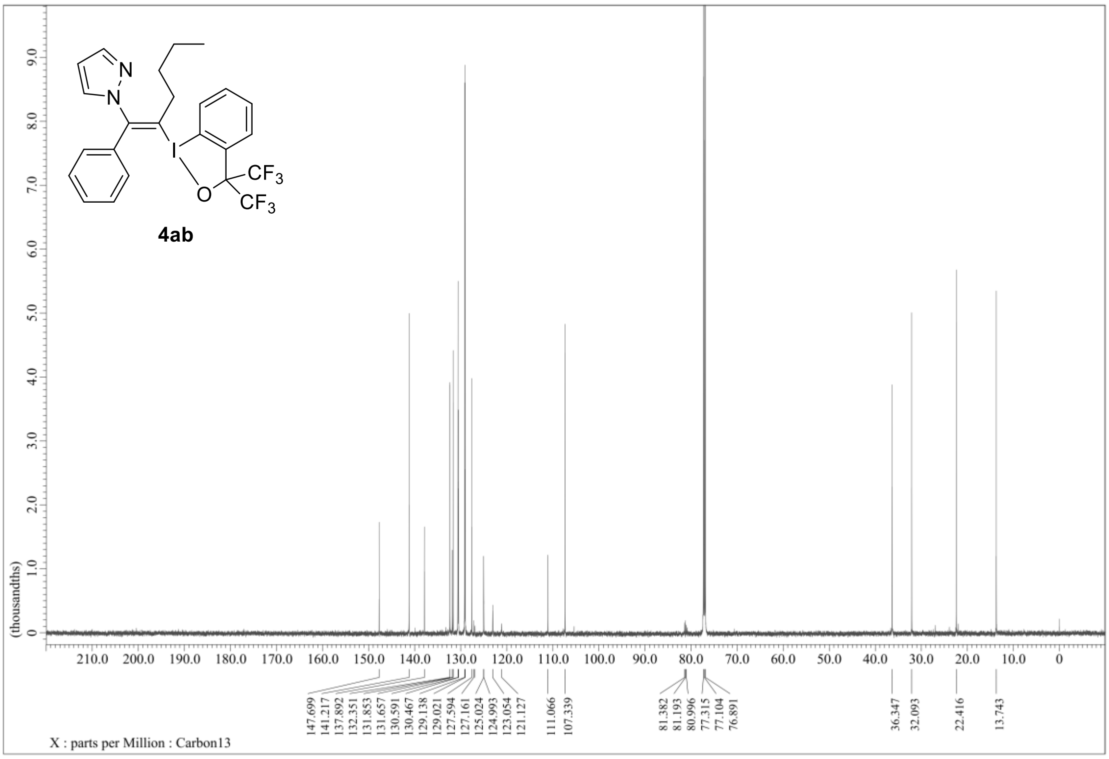

$^{19}F\{^{1}H\}$ NMR (565 MHz, $CDCl_3$) spectrum of **4ab**

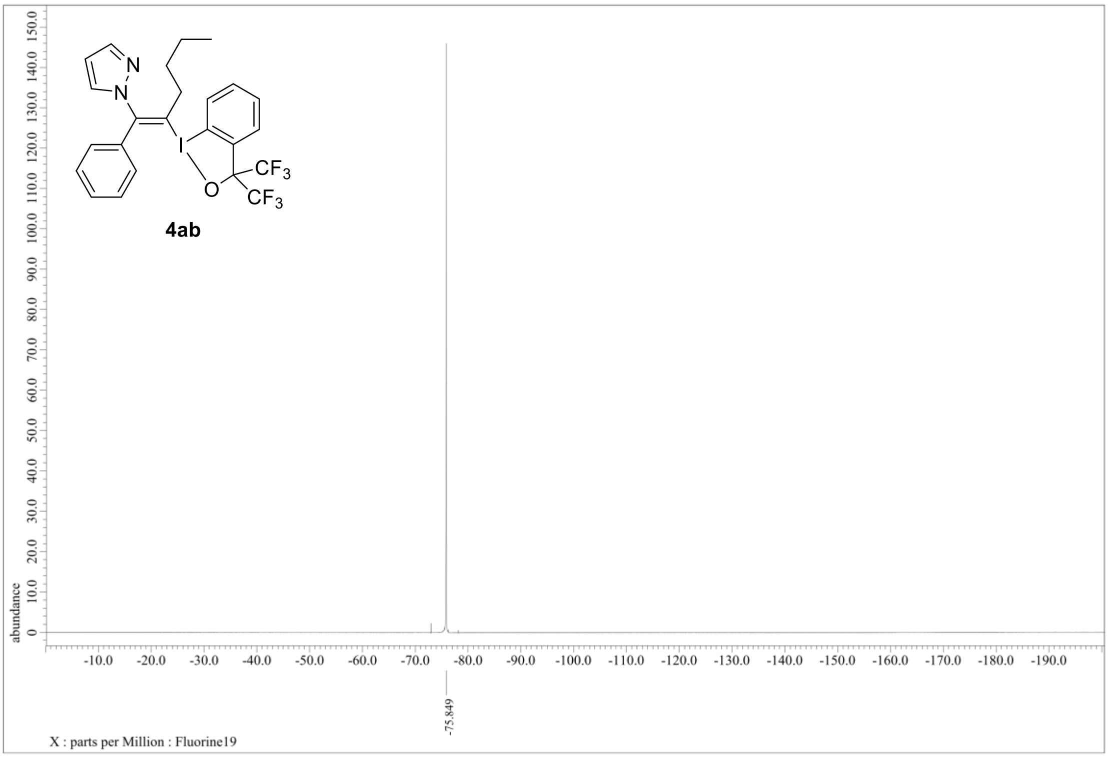

^{1}H NMR (600 MHz, $CDCl_3$) and $^{13}C\{^{1}H\}$ NMR (151 MHz, $CDCl_3$) spectra of **4ac**

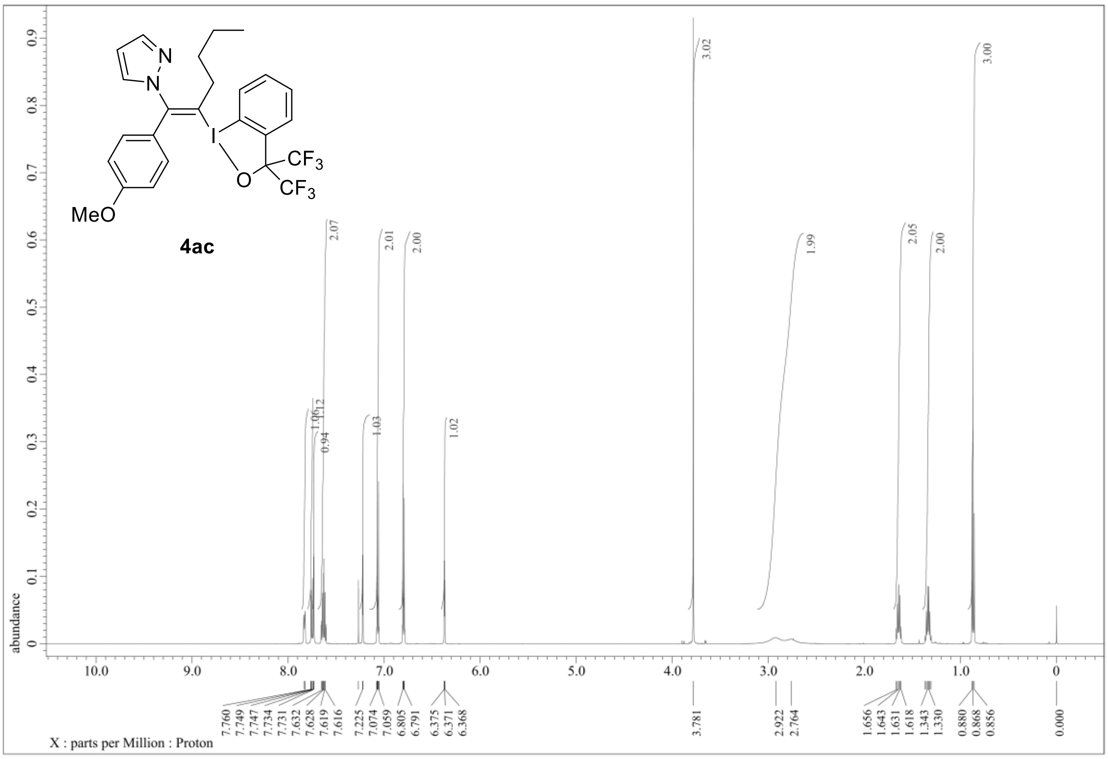

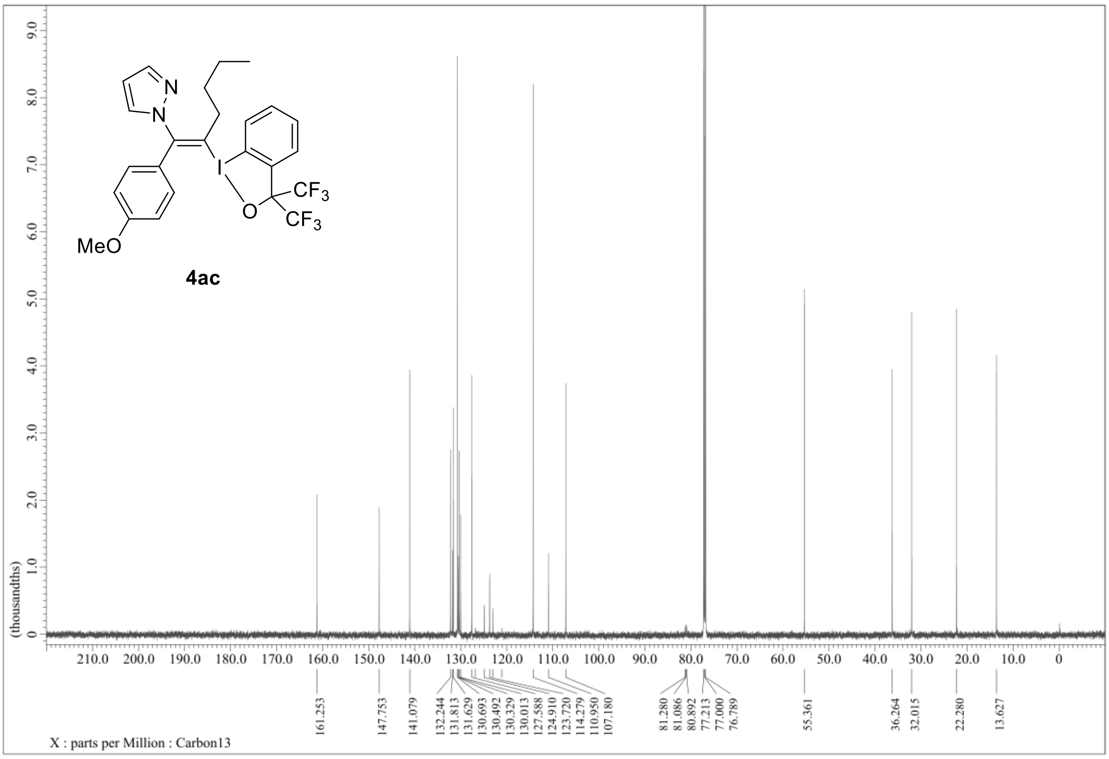

$^{19}F\{^1H\}$ NMR (565 MHz, $CDCl_3$) spectrum of **4ac**

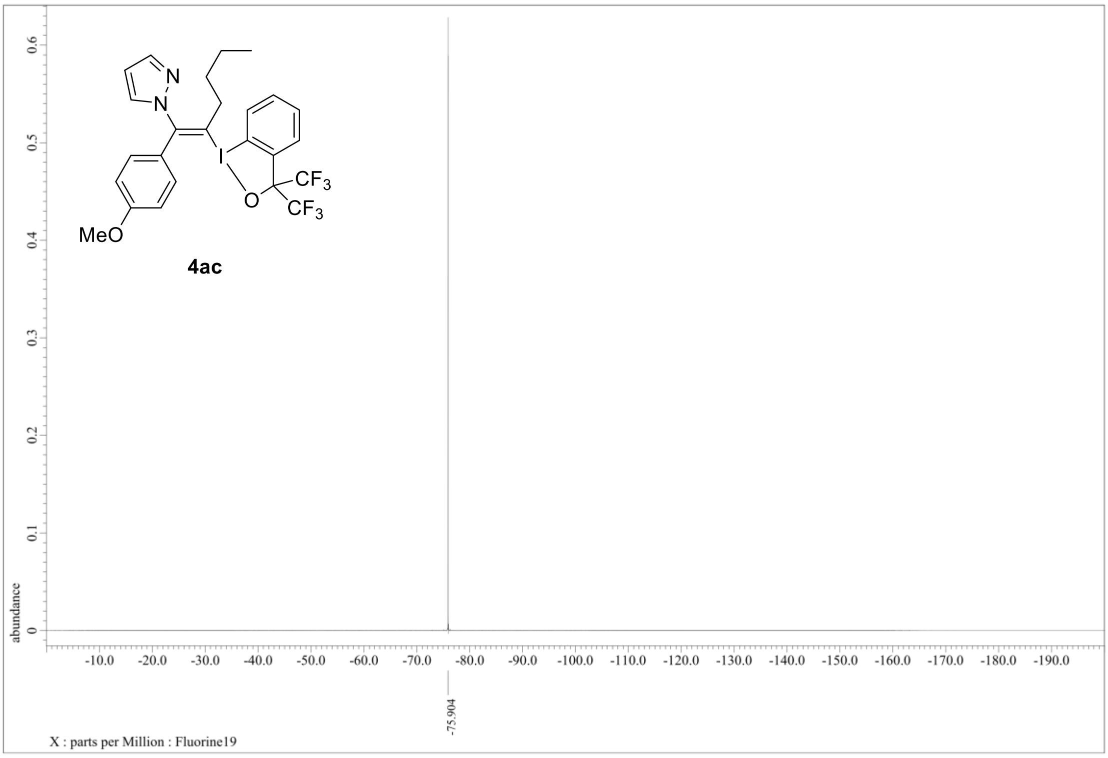

^{1}H NMR (600 MHz, $CDCl_3$) and $^{13}C\{^1H\}$ NMR (151 MHz, $CDCl_3$) spectra of **4ad**

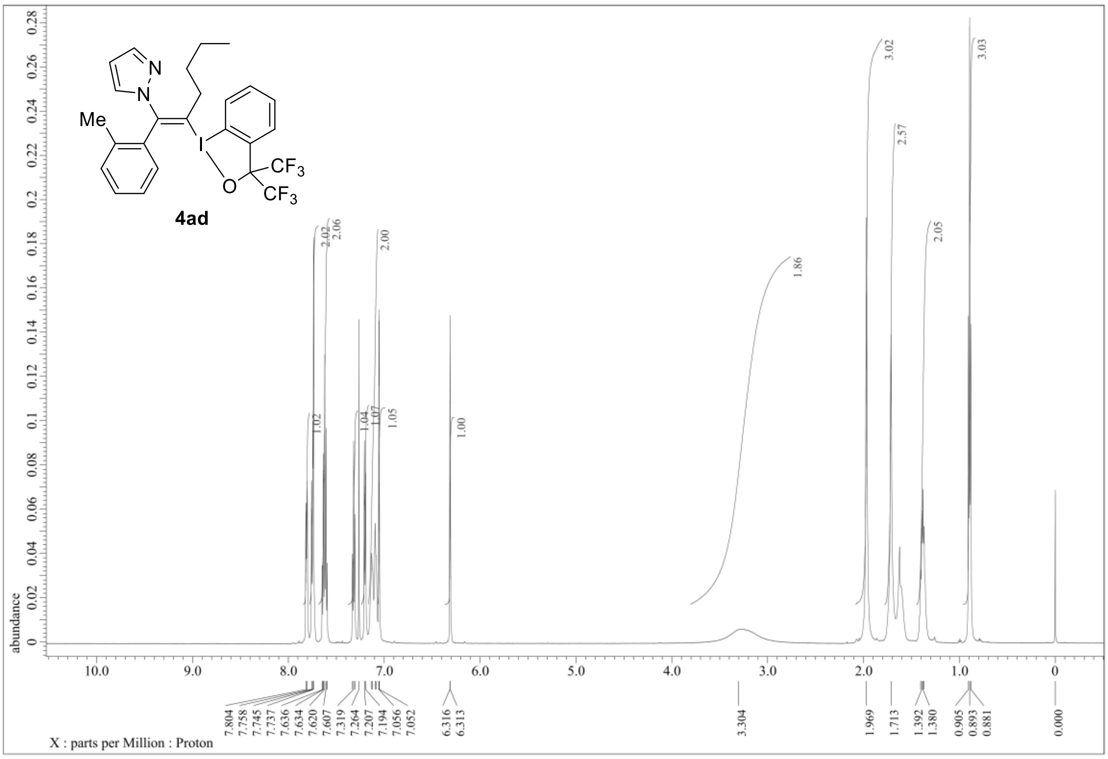

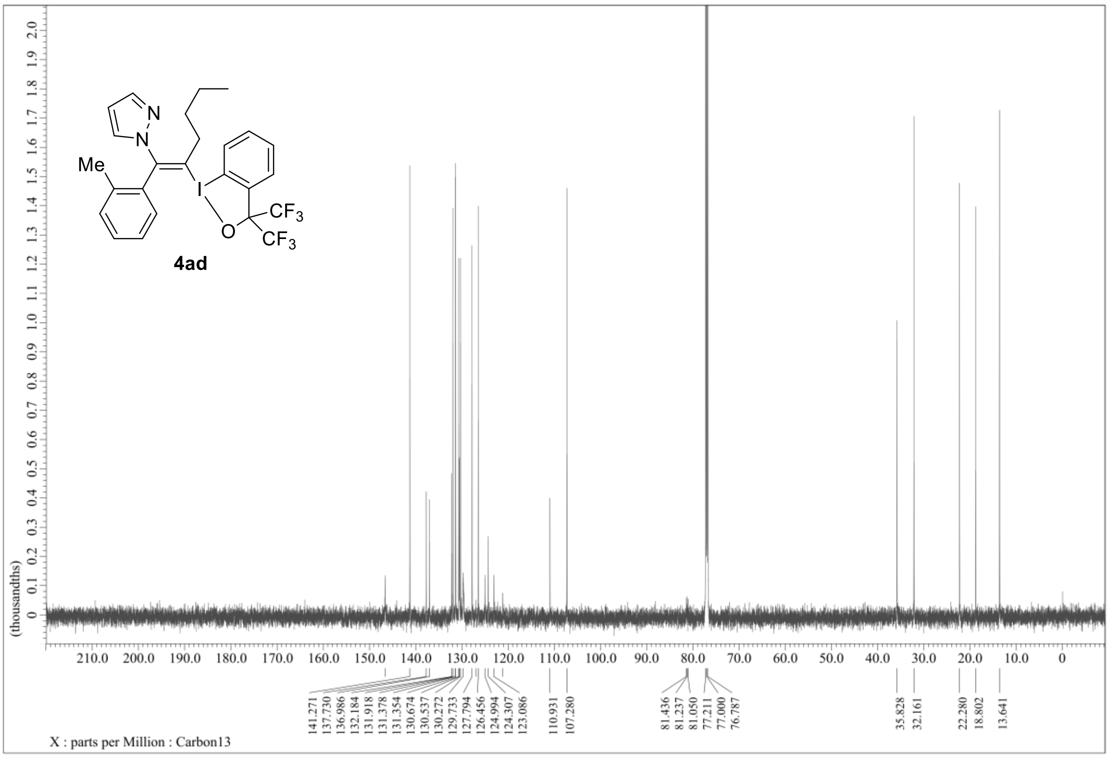

$^{19}F\{^1H\}$ NMR (565 MHz, $CDCl_3$) spectrum of **4ad**

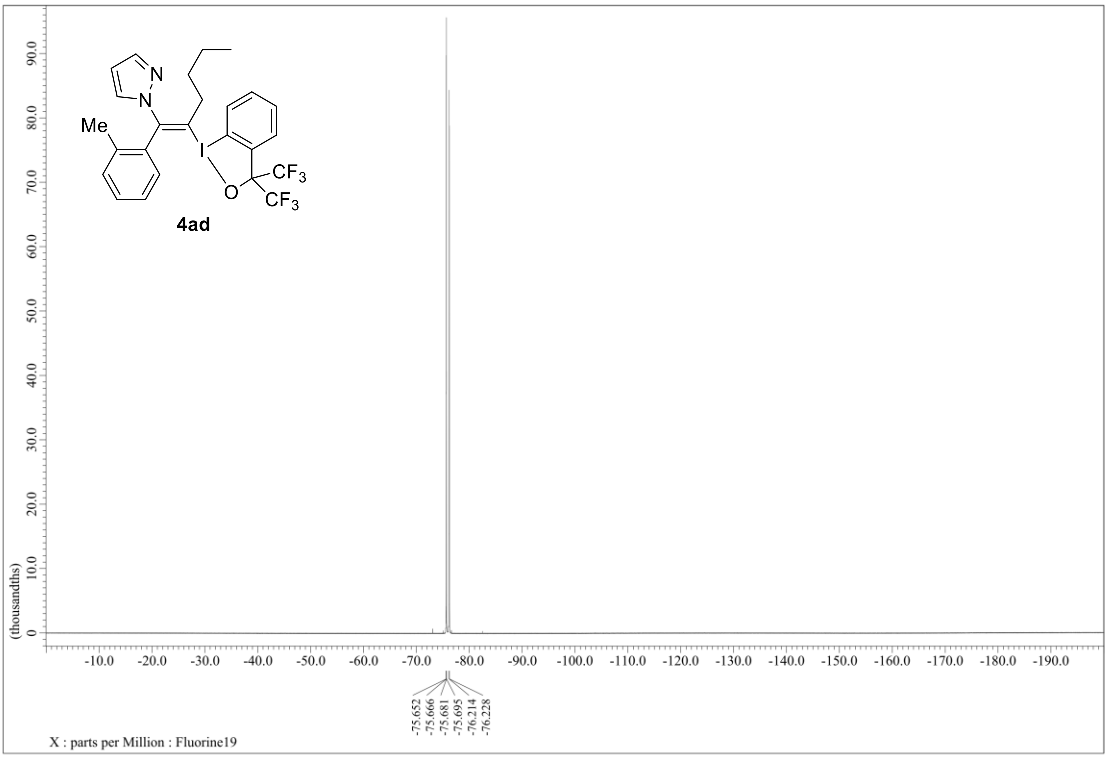

^1H NMR (600 MHz, $CDCl_3$) and $^{13}C\{^1H\}$ NMR (151 MHz, $CDCl_3$) spectra of **4ae**

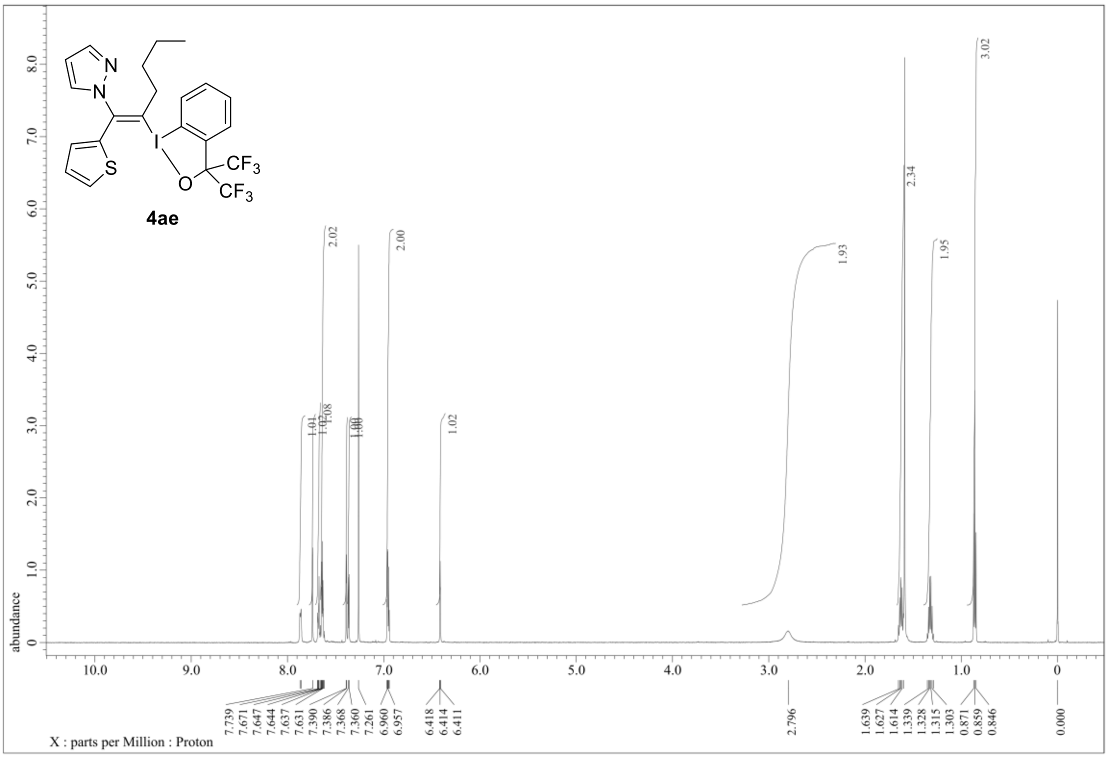

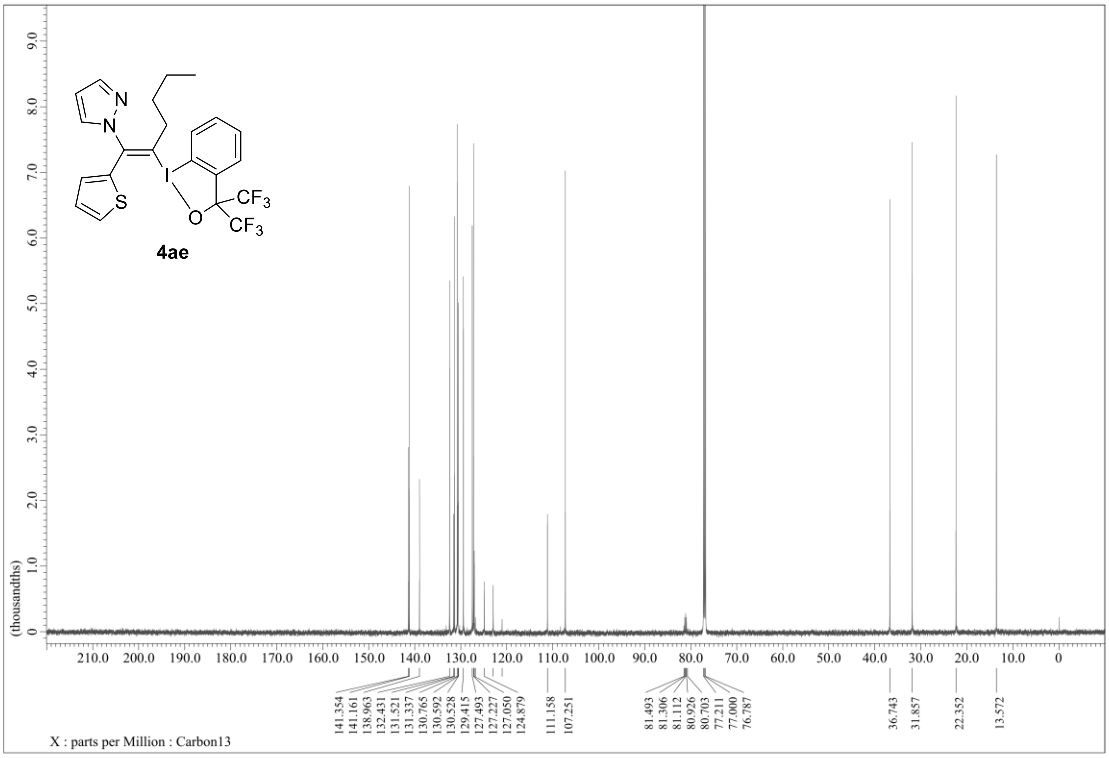

$^{19}F\{^1H\}$ NMR (565 MHz, $CDCl_3$) spectrum of **4ae**

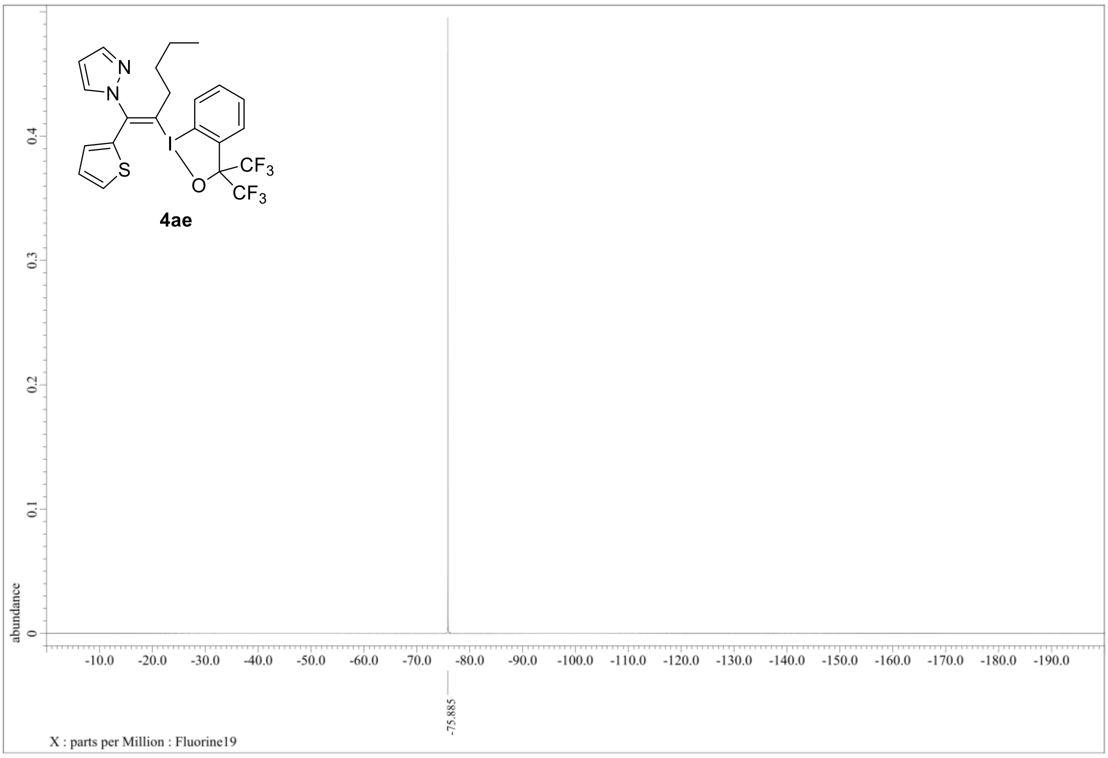

^{1}H NMR (600 MHz, $CDCl_3$) and $^{13}C\{^{1}H\}$ NMR (151 MHz, $CDCl_3$) spectra of **4af**

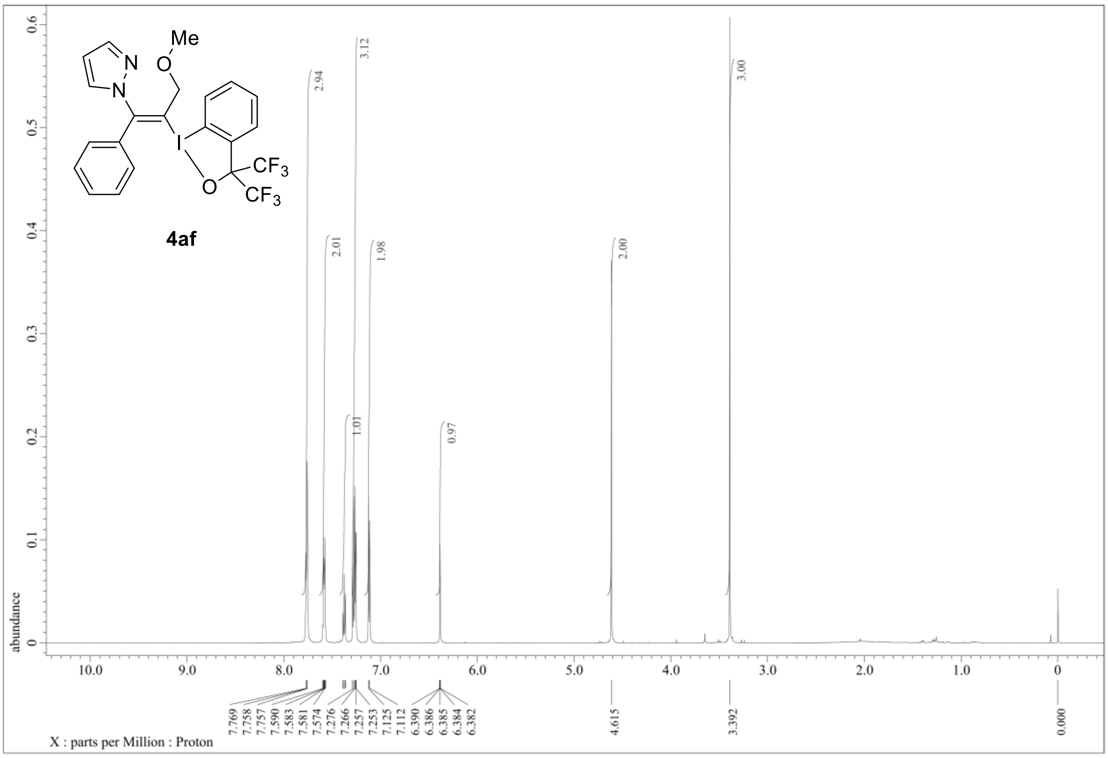

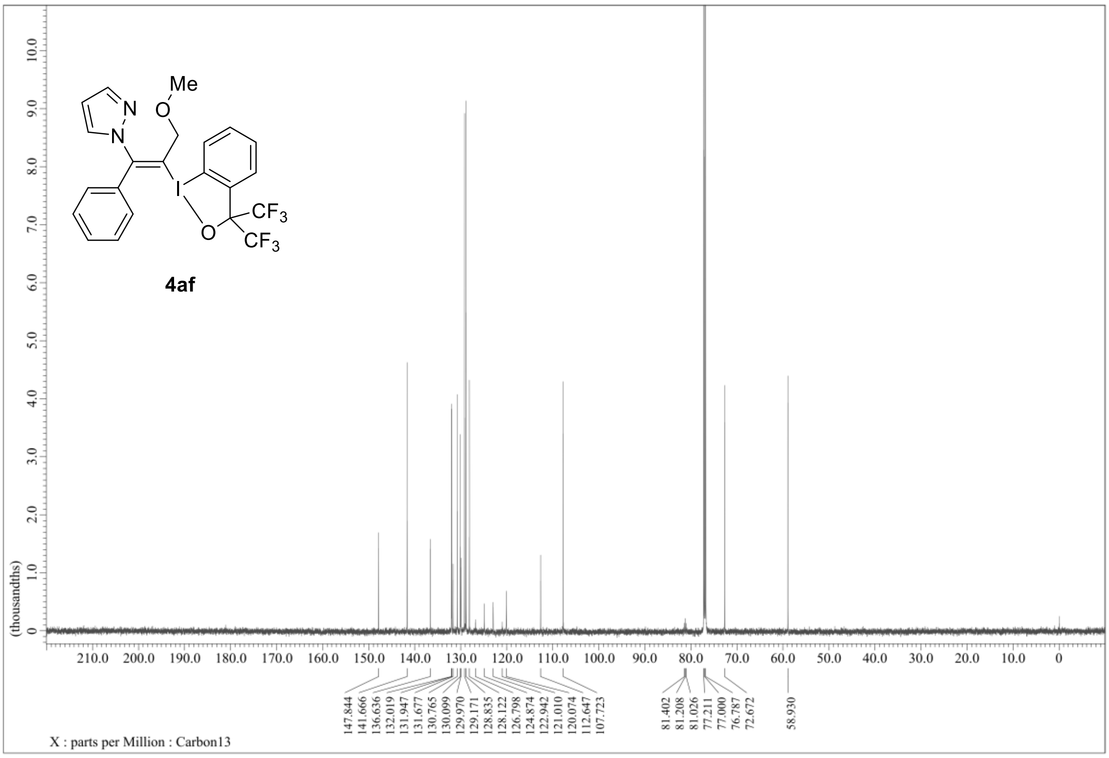

$^{19}F\{^{1}H\}$ NMR (565 MHz, $CDCl_3$) spectrum of **4af**

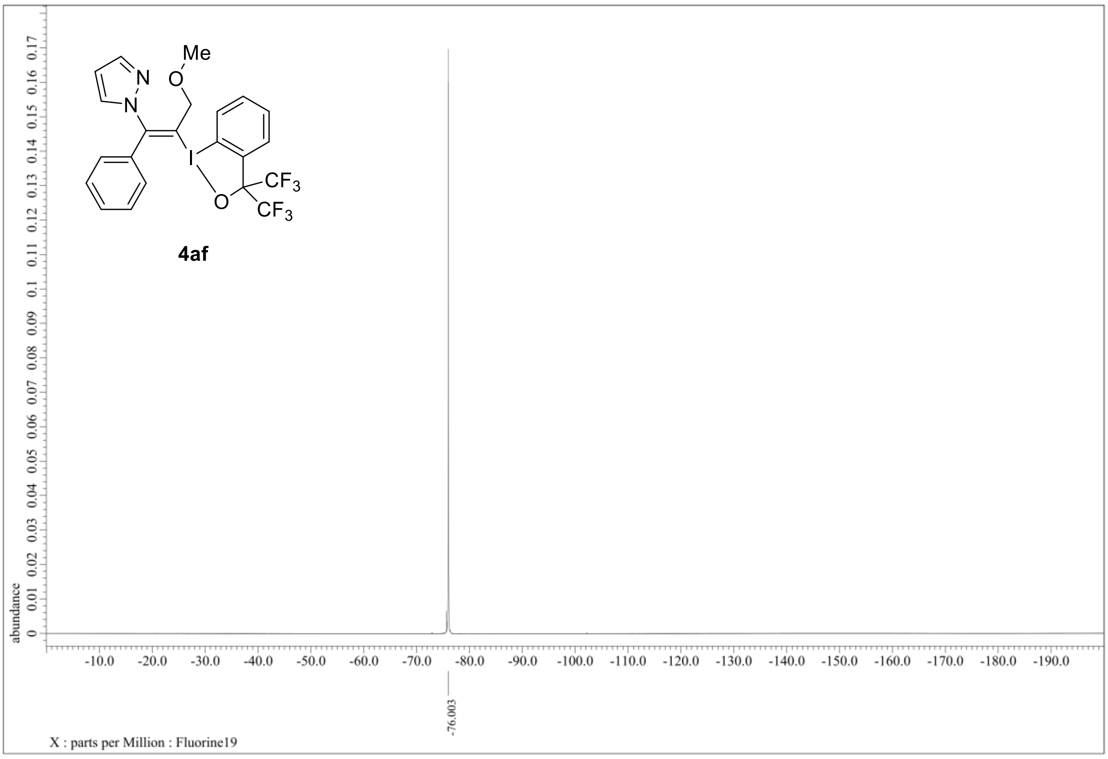

^{1}H NMR (600 MHz, $CDCl_3$) and $^{13}C\{^{1}H\}$ NMR (151 MHz, $CDCl_3$) spectra of **4ag**

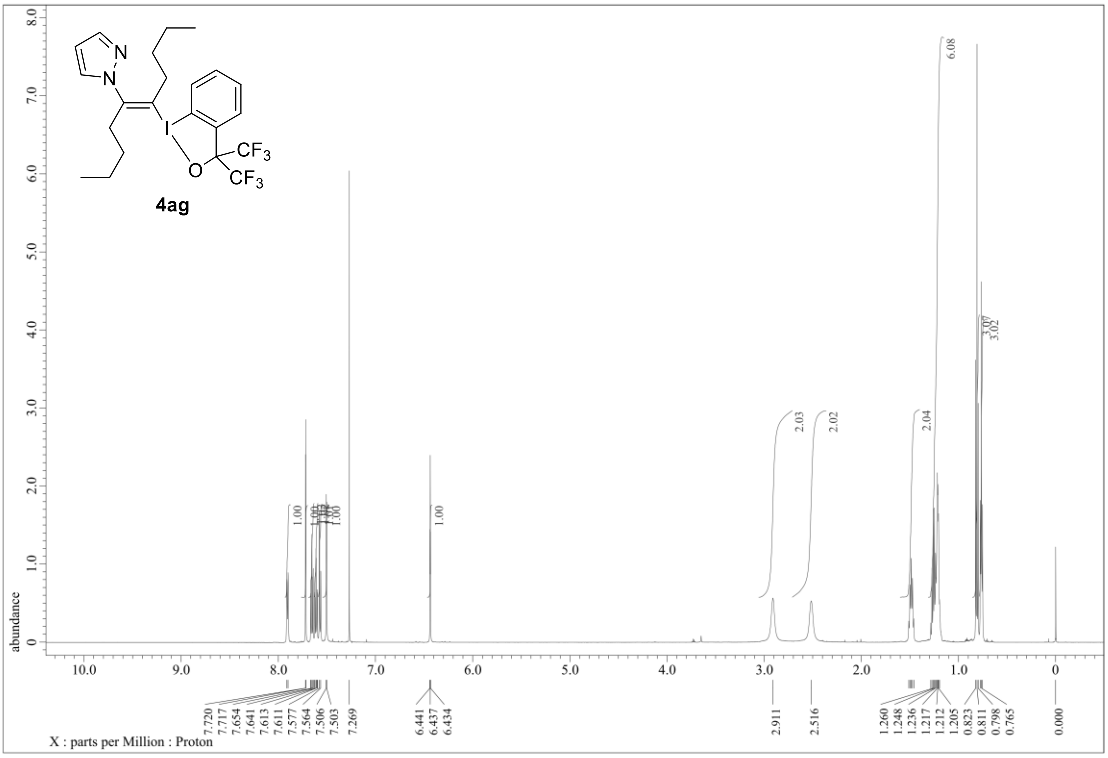

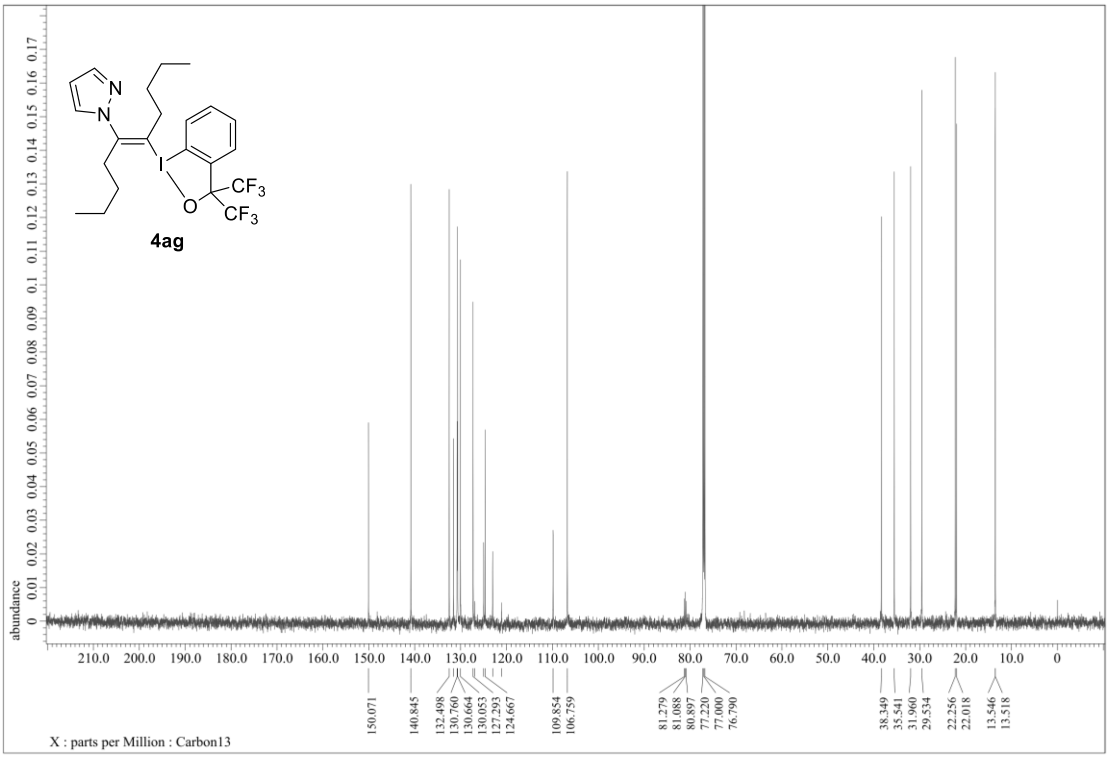

$^{19}F\{^{1}H\}$ NMR (565 MHz, $CDCl_3$) spectrum of **4ag**

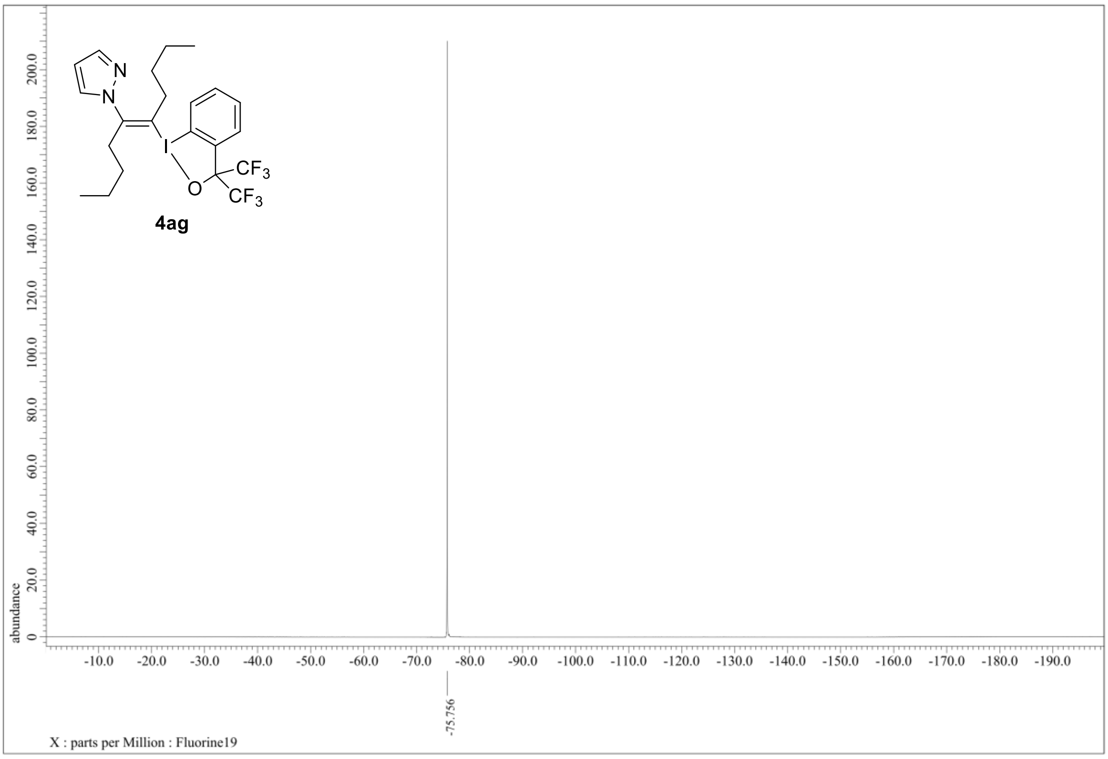

^{1}H NMR (600 MHz, $CDCl_3$) and ^{13}C{^{1}H} NMR (151 MHz, $CDCl_3$) spectra of **4ah**

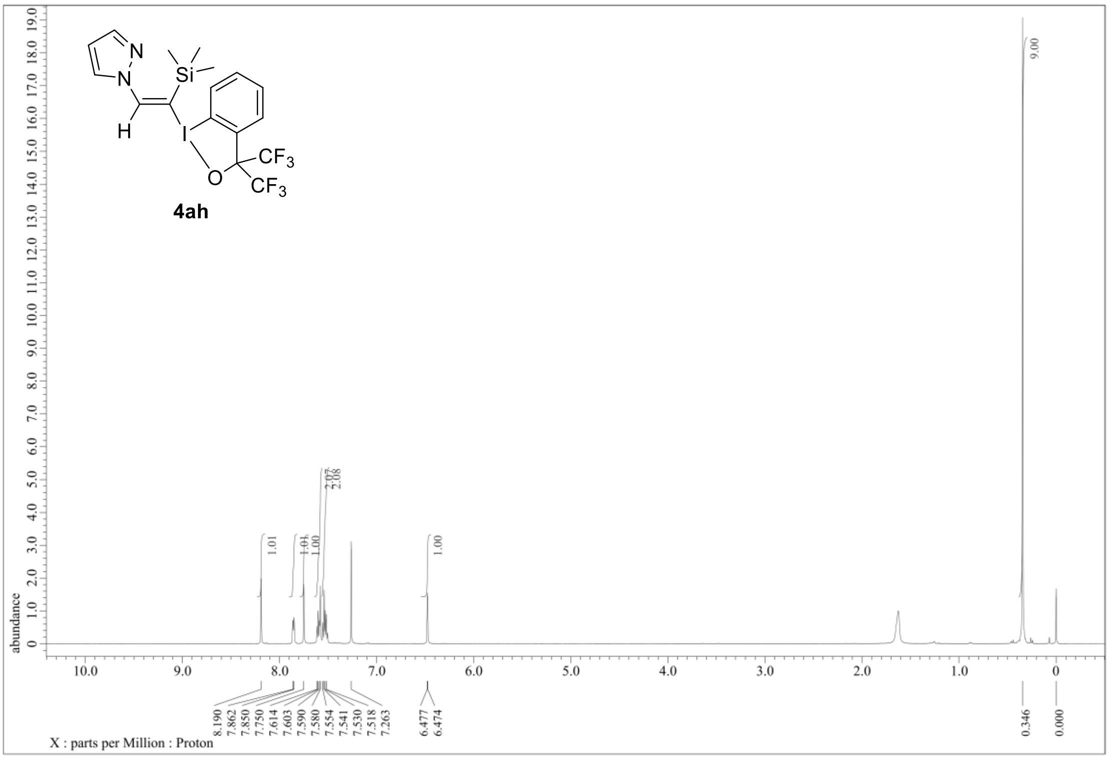

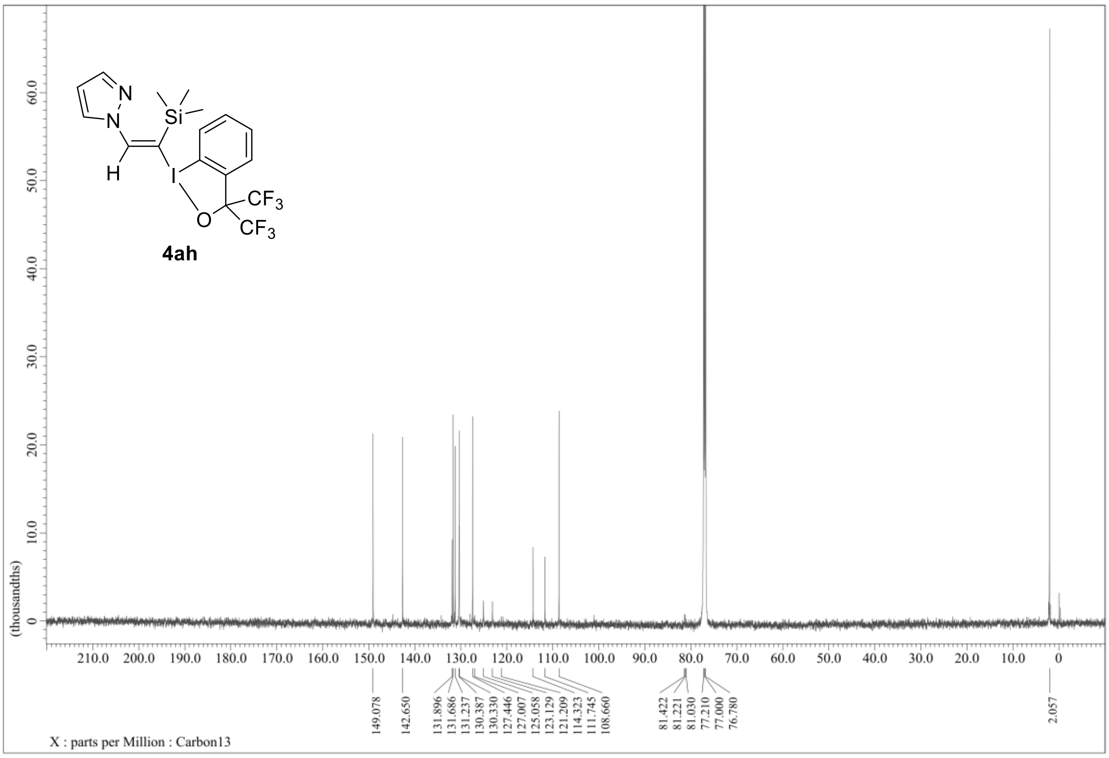

$^{19}F\{^1H\}$ NMR (565 MHz, $CDCl_3$) spectrum of **4ah**

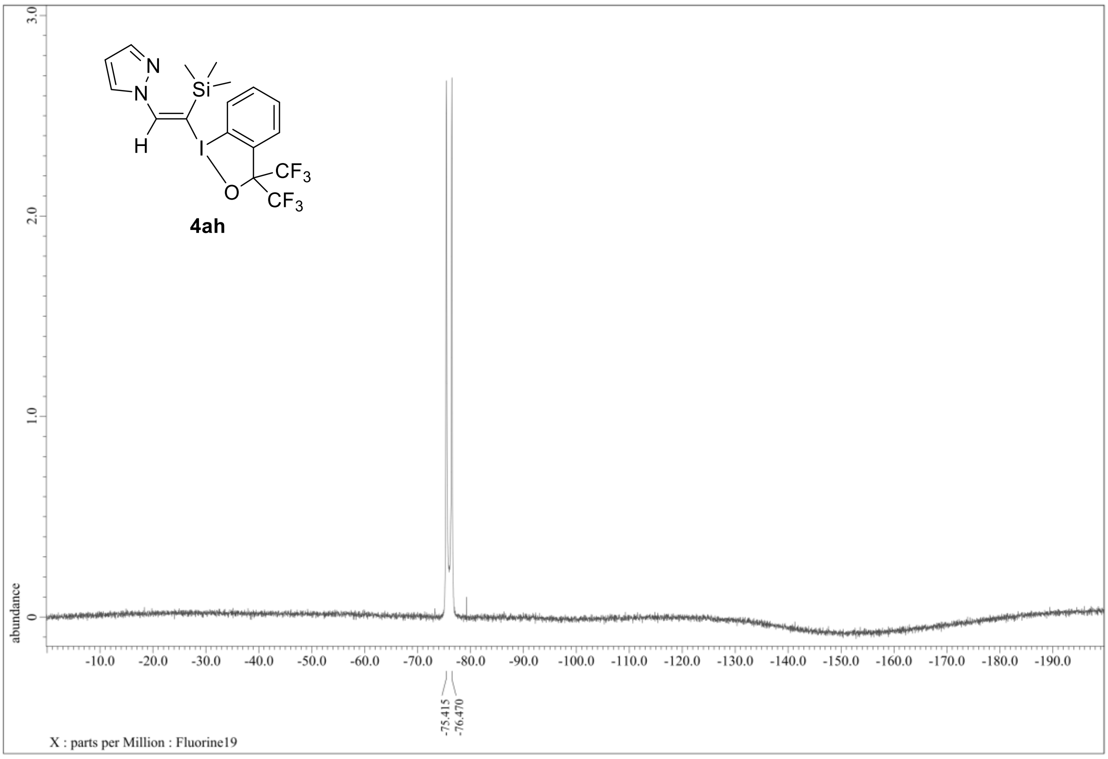

^{1}H NMR (600 MHz, $CDCl_3$) and $^{13}C\{^{1}H\}$ NMR (151 MHz, $CDCl_3$) spectra of **4ai**

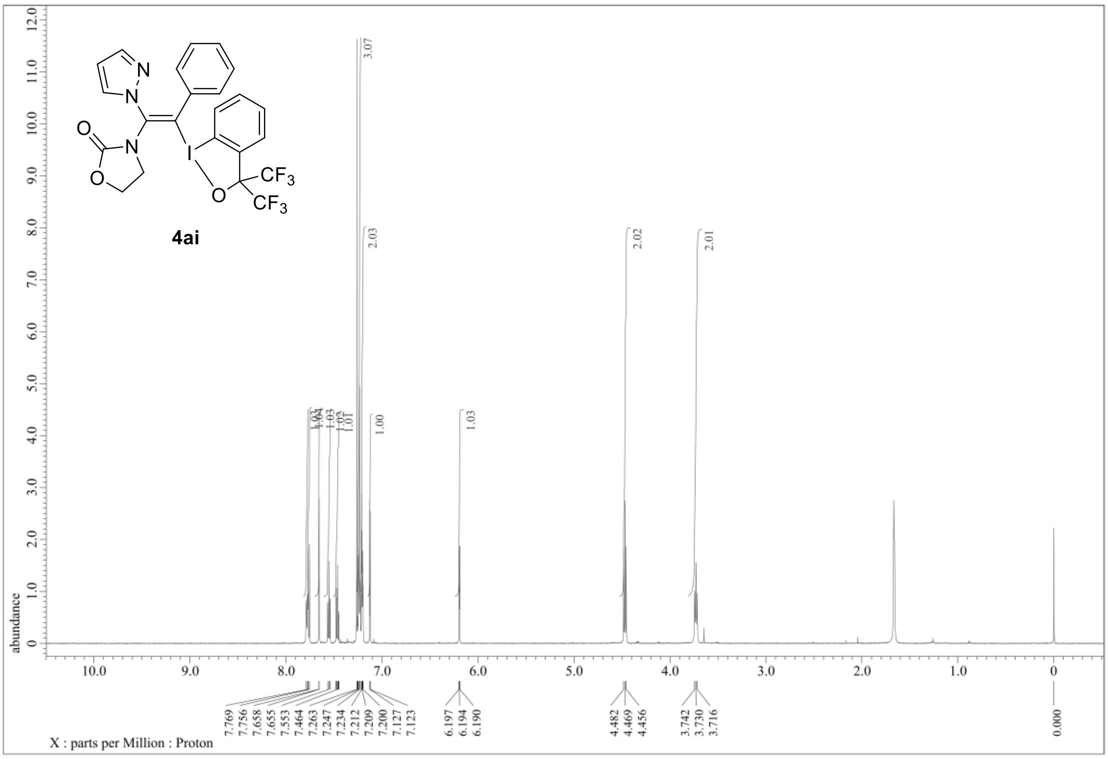

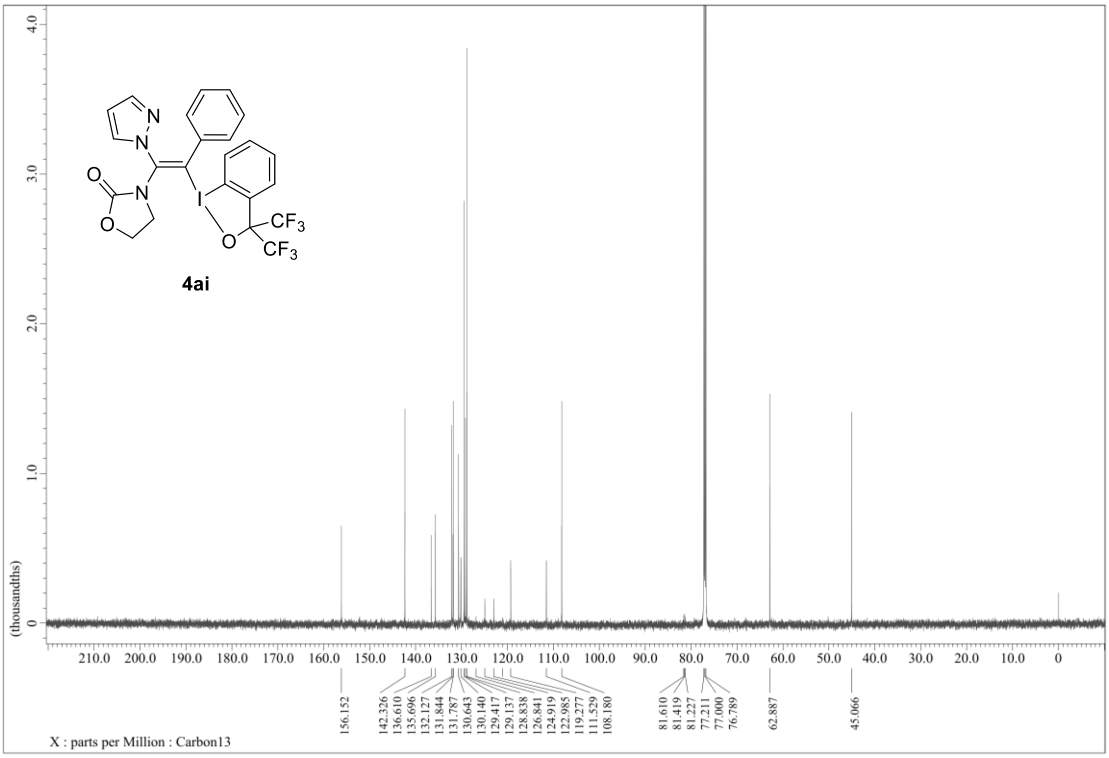

$^{19}F\{^1H\}$ NMR (565 MHz, $CDCl_3$) spectrum of **4ai**

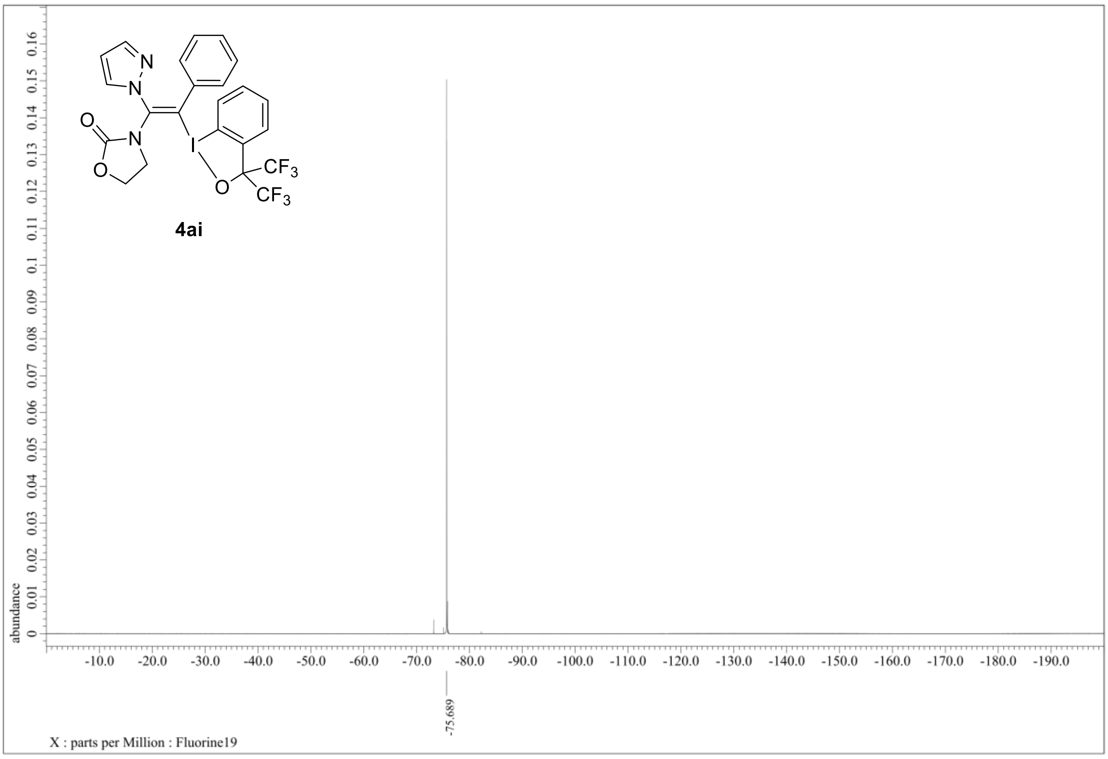

^{1}H NMR (600 MHz, $CDCl_3$) and $^{13}C\{^{1}H\}$ NMR (151 MHz, $CDCl_3$) spectra of **5**

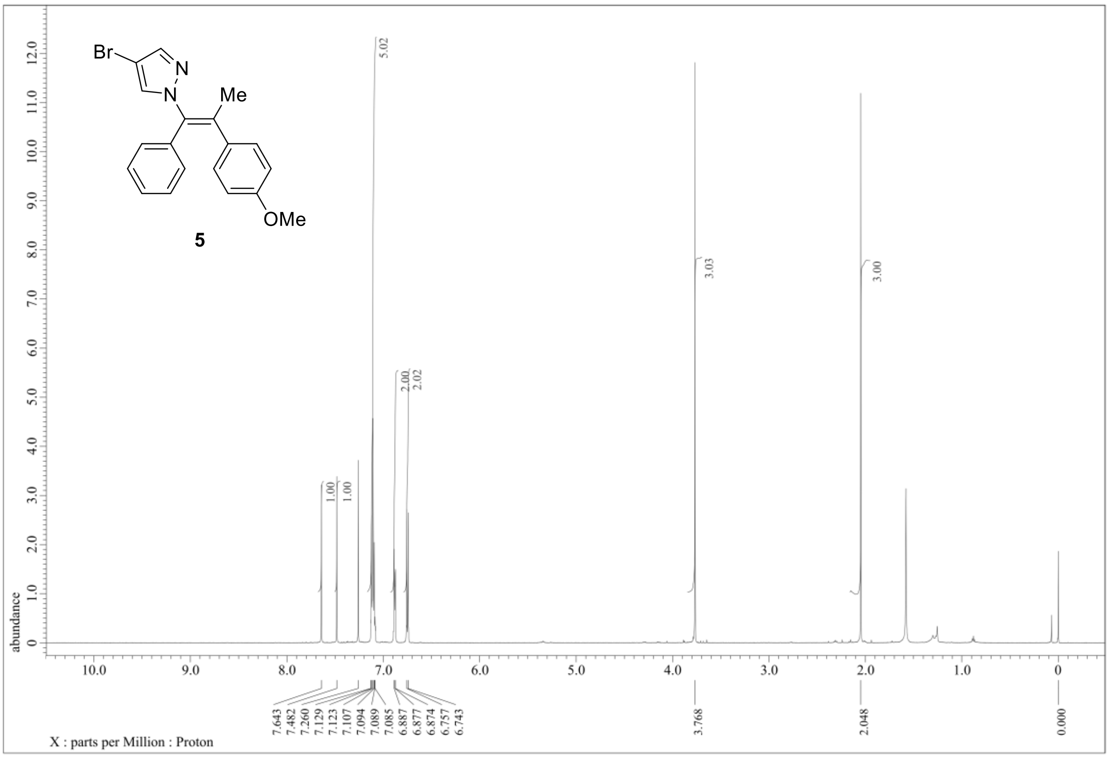

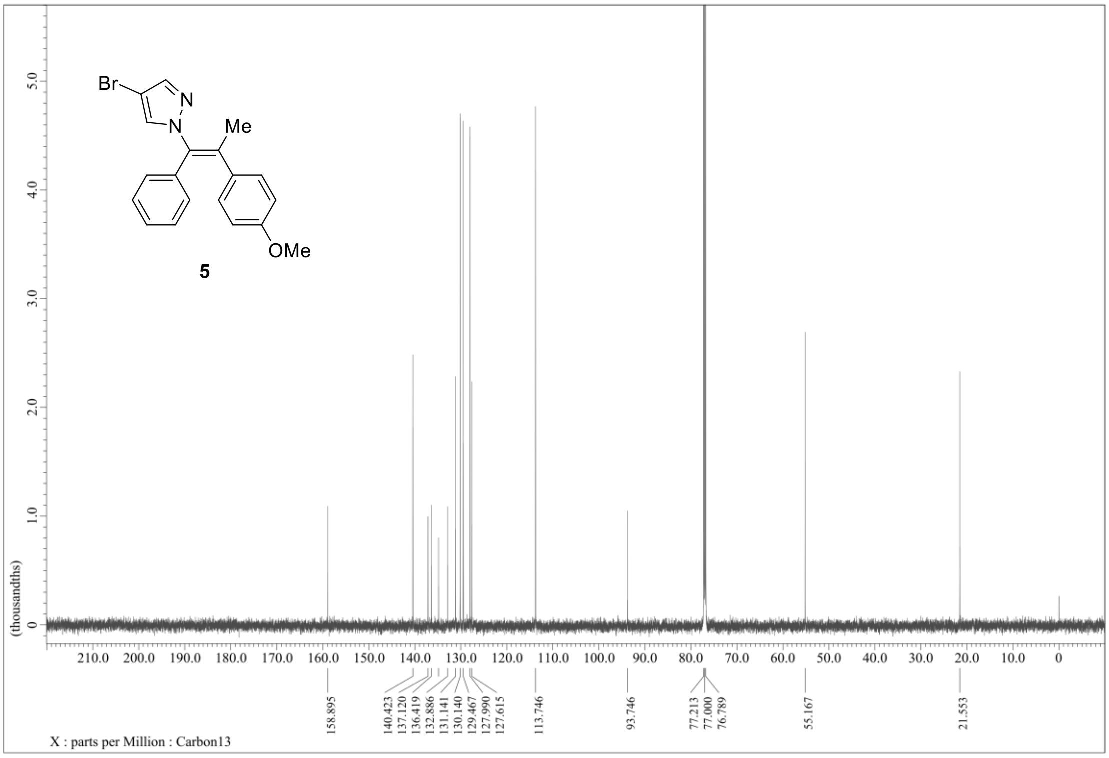

^{1}H NMR (600 MHz, $CDCl_3$) and $^{13}C\{^{1}H\}$ NMR (151 MHz, $CDCl_3$) spectra of **6**

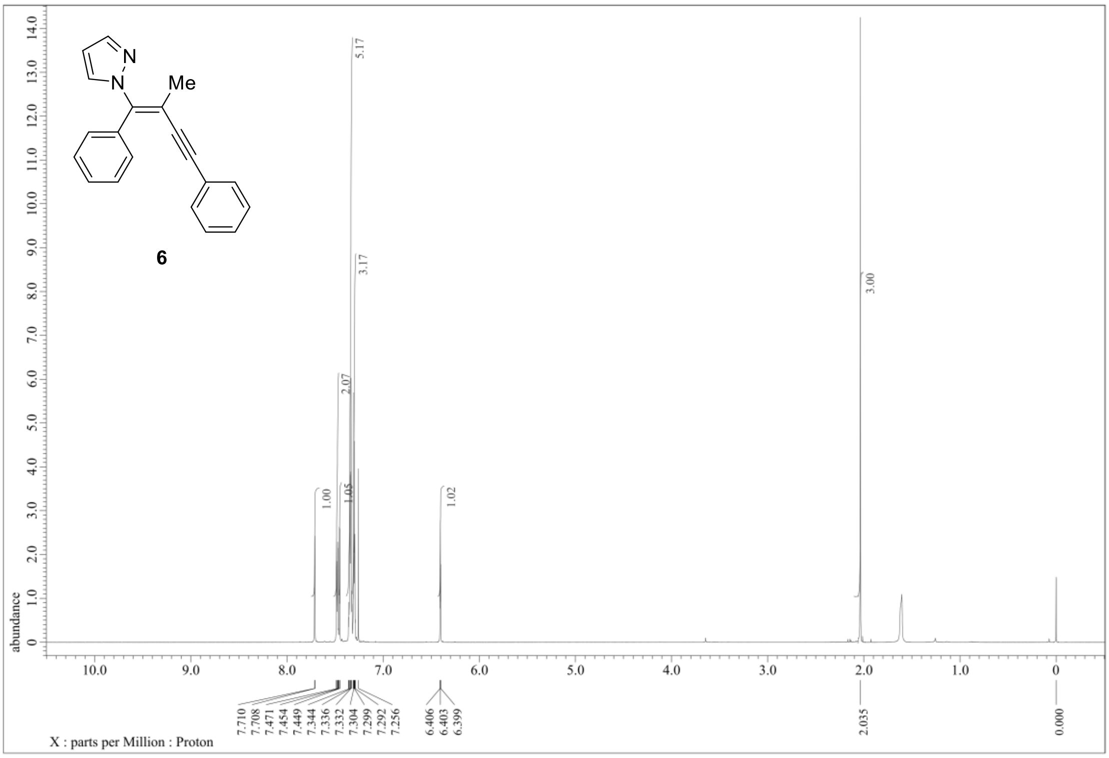

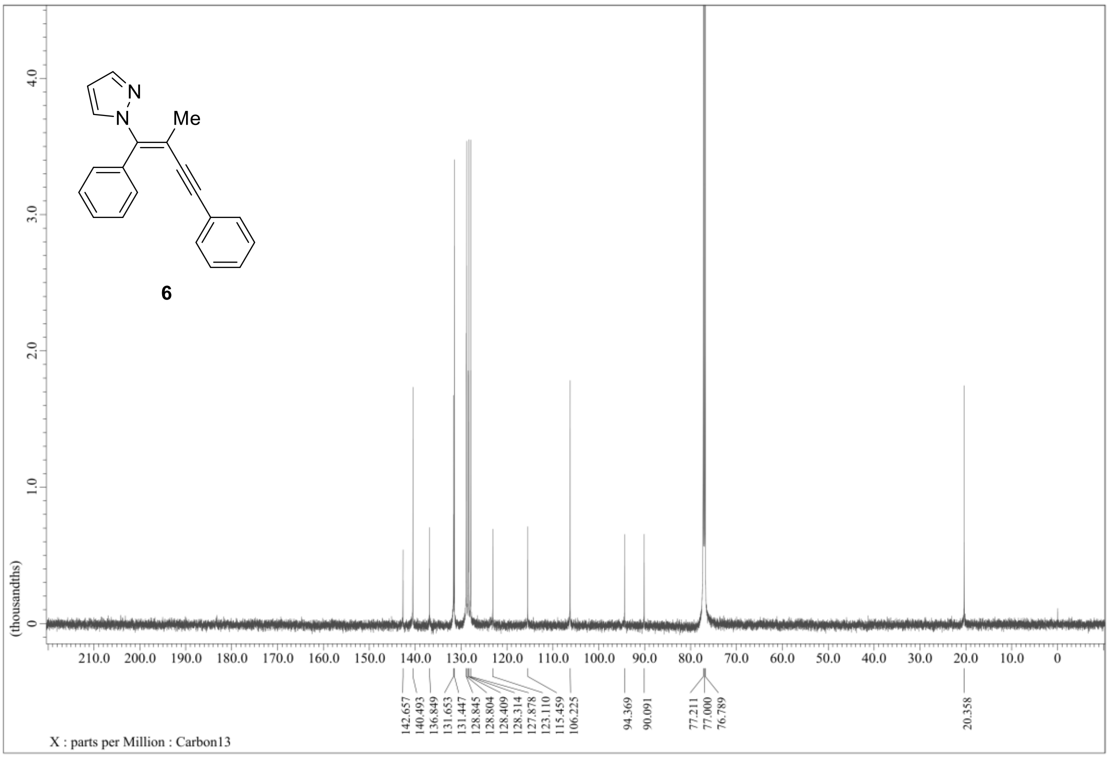

^{1}H NMR (600 MHz, $CDCl_3$) and ^{13}C{^{1}H} NMR (151 MHz, $CDCl_3$) spectra of **7**

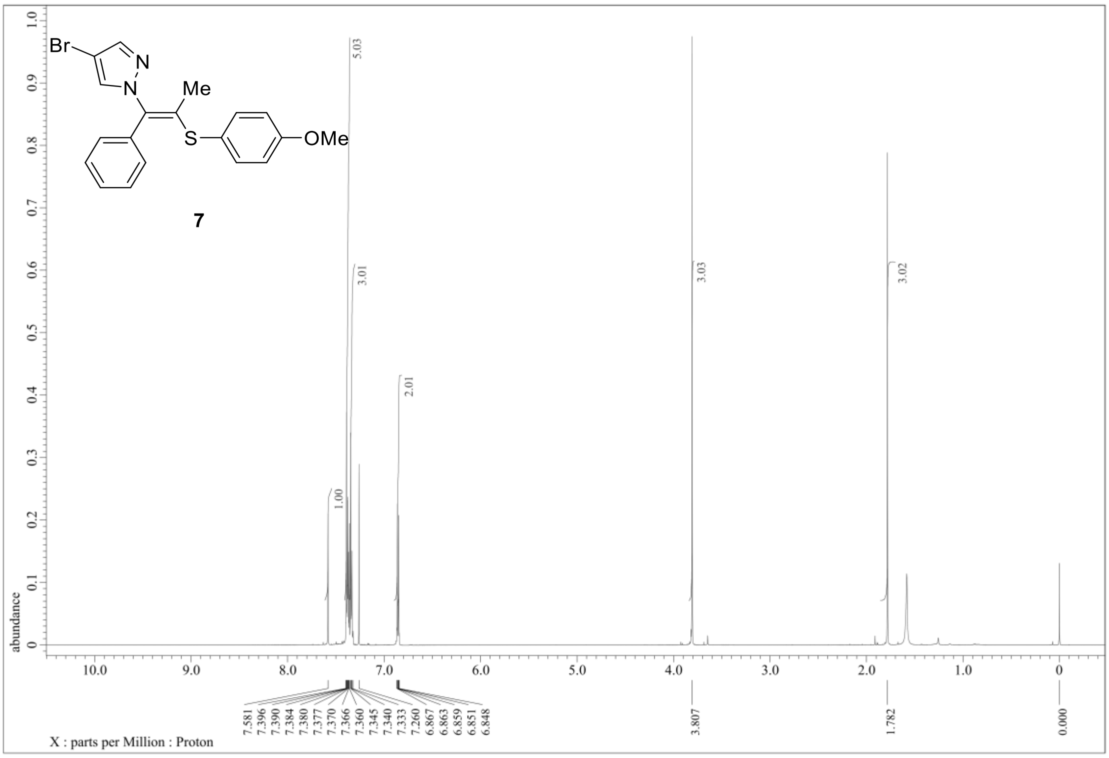

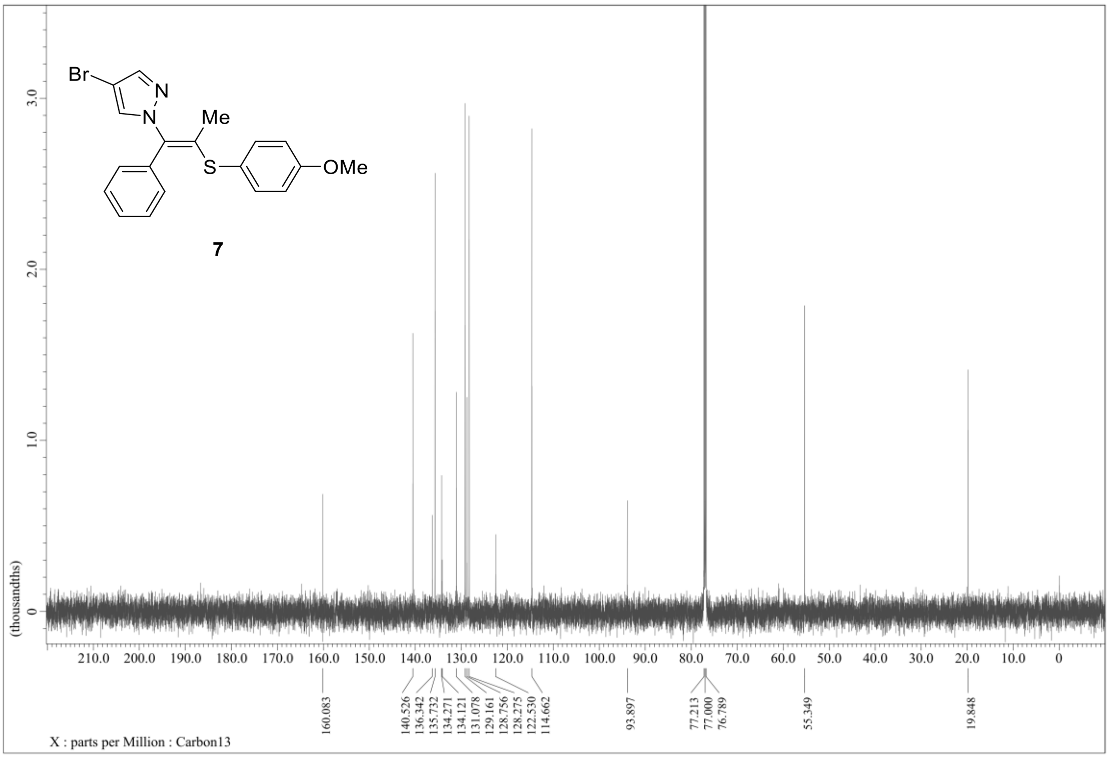

^1H NMR (600 MHz, $CDCl_3$) and $^{13}C\{^1H\}$ NMR (151 MHz, $CDCl_3$) spectra of **8**

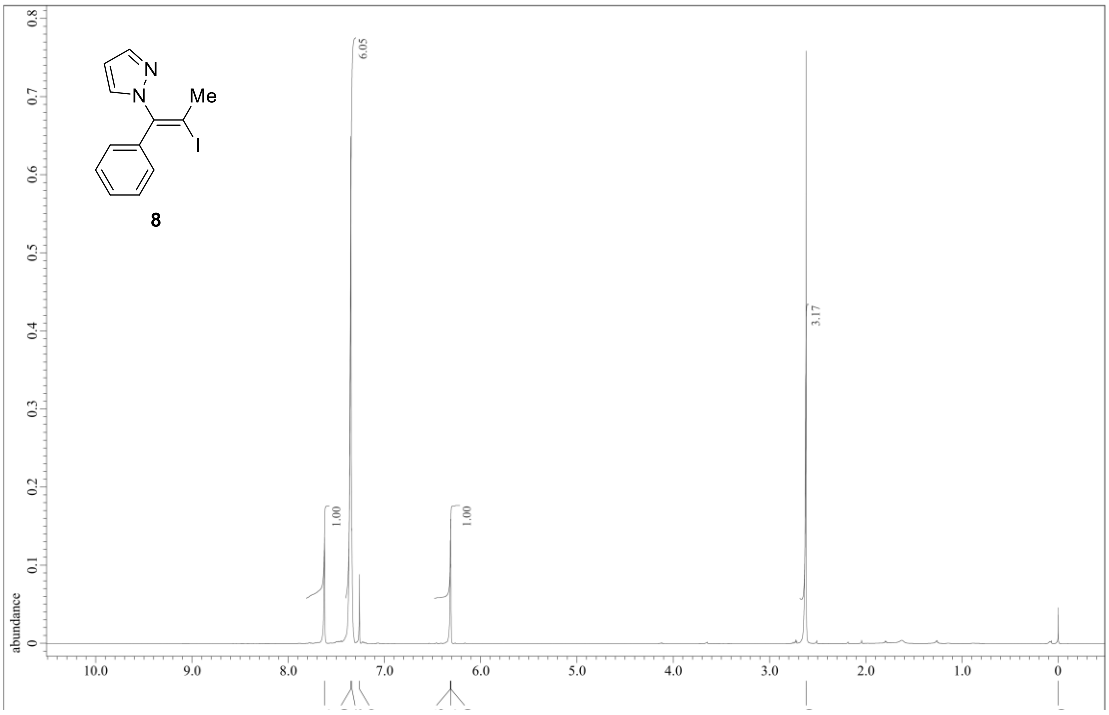

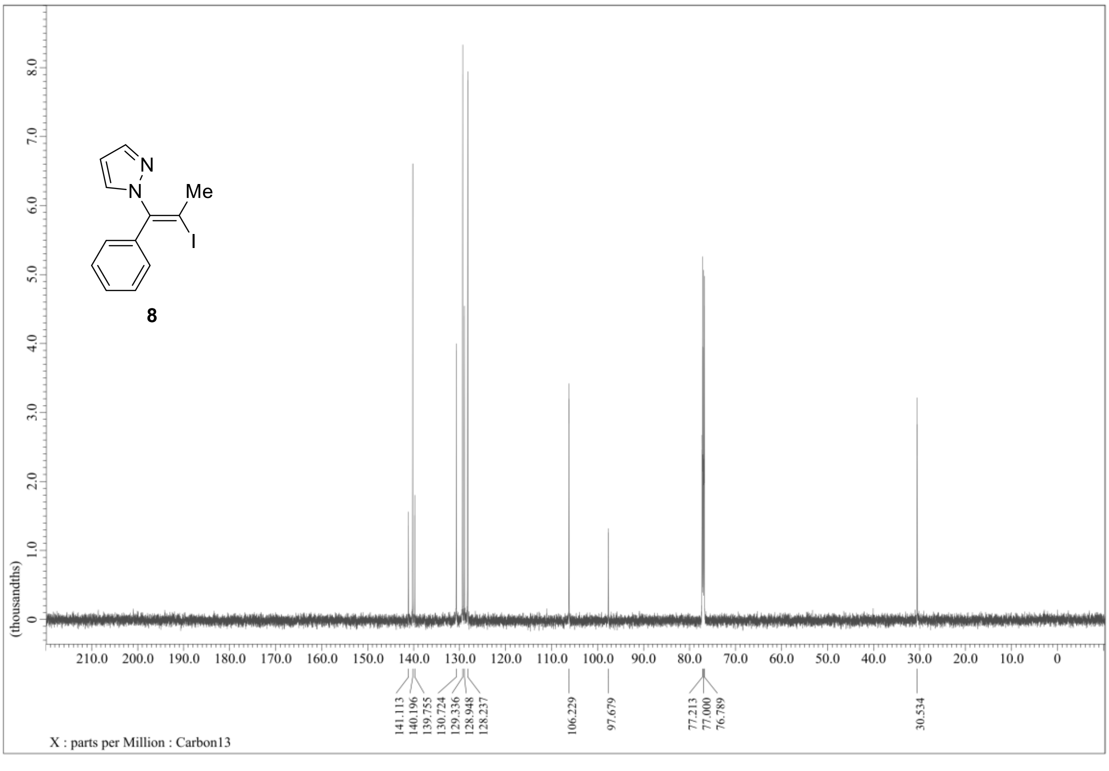

^{1}H NMR (600 MHz, $CDCl_3$) and ^{13}C{^{1}H} NMR (151 MHz, $CDCl_3$) spectra of **9**

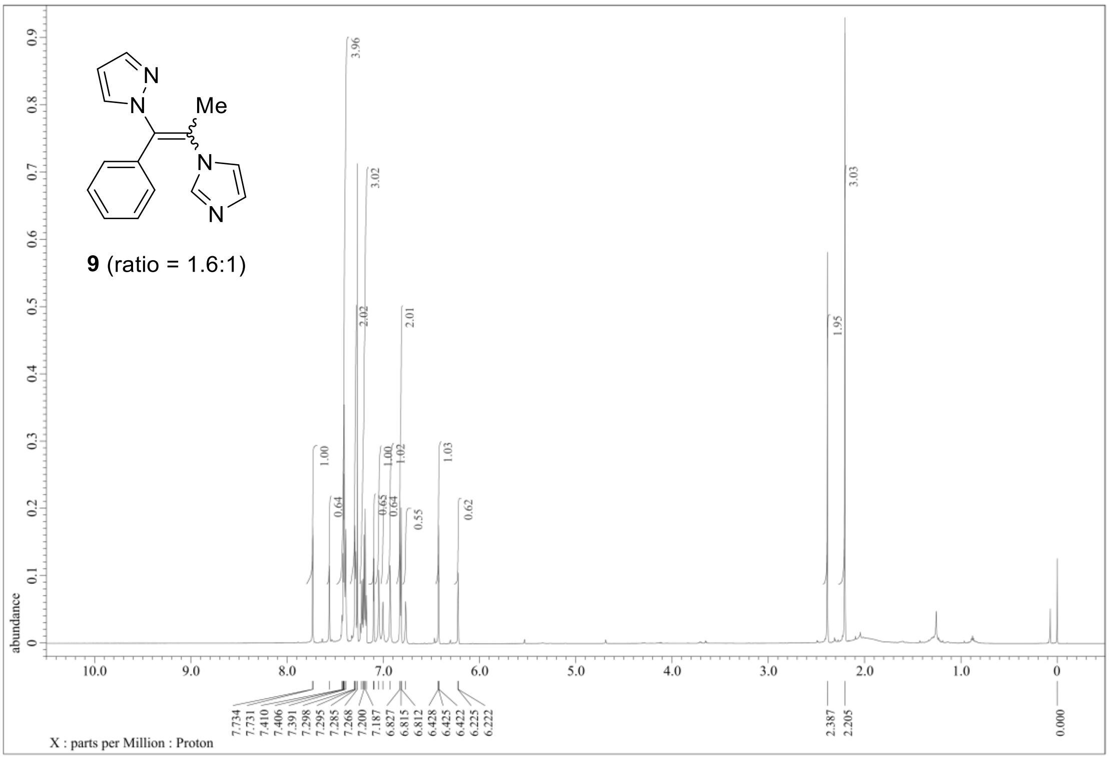

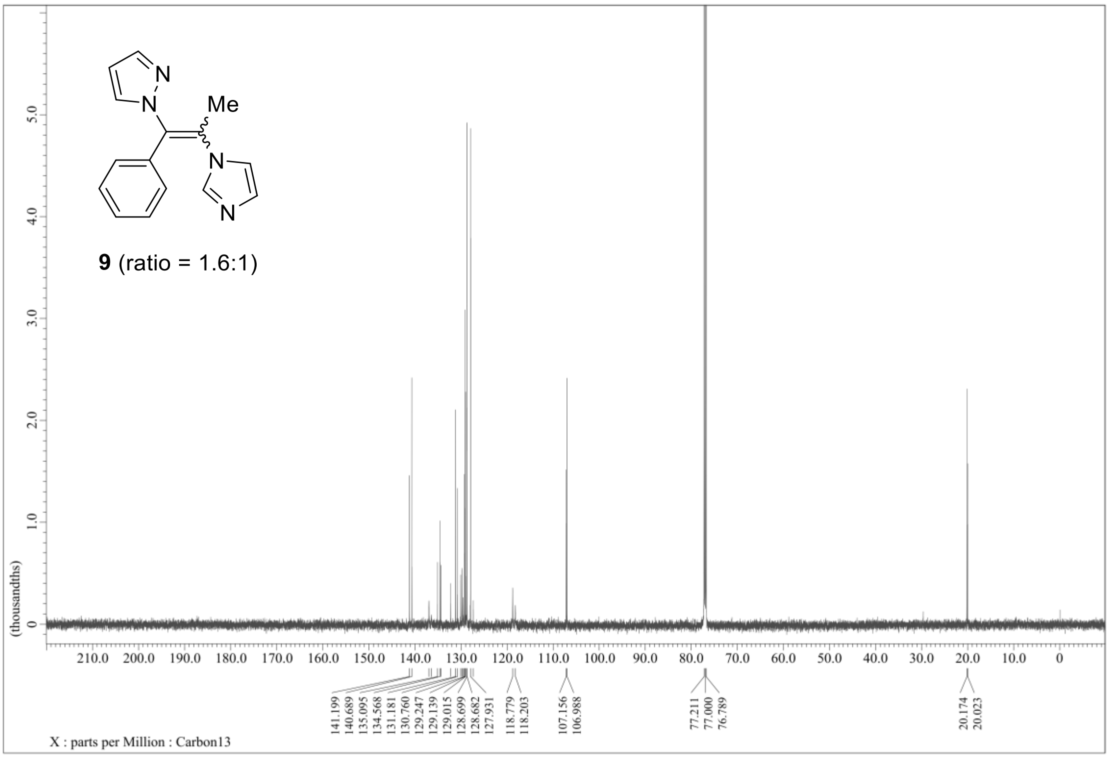

^{1}H NMR (600 MHz, $CDCl_3$) and $^{13}C\{^{1}H\}$ NMR (151 MHz, $CDCl_3$) spectra of **10**

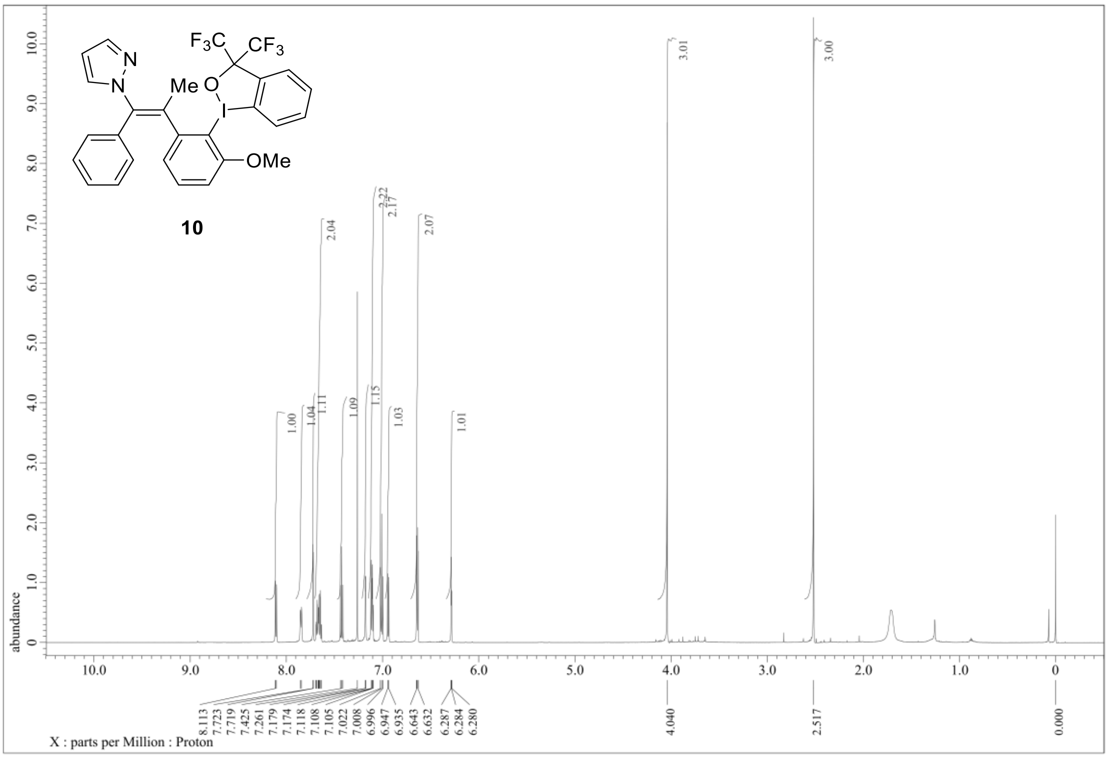

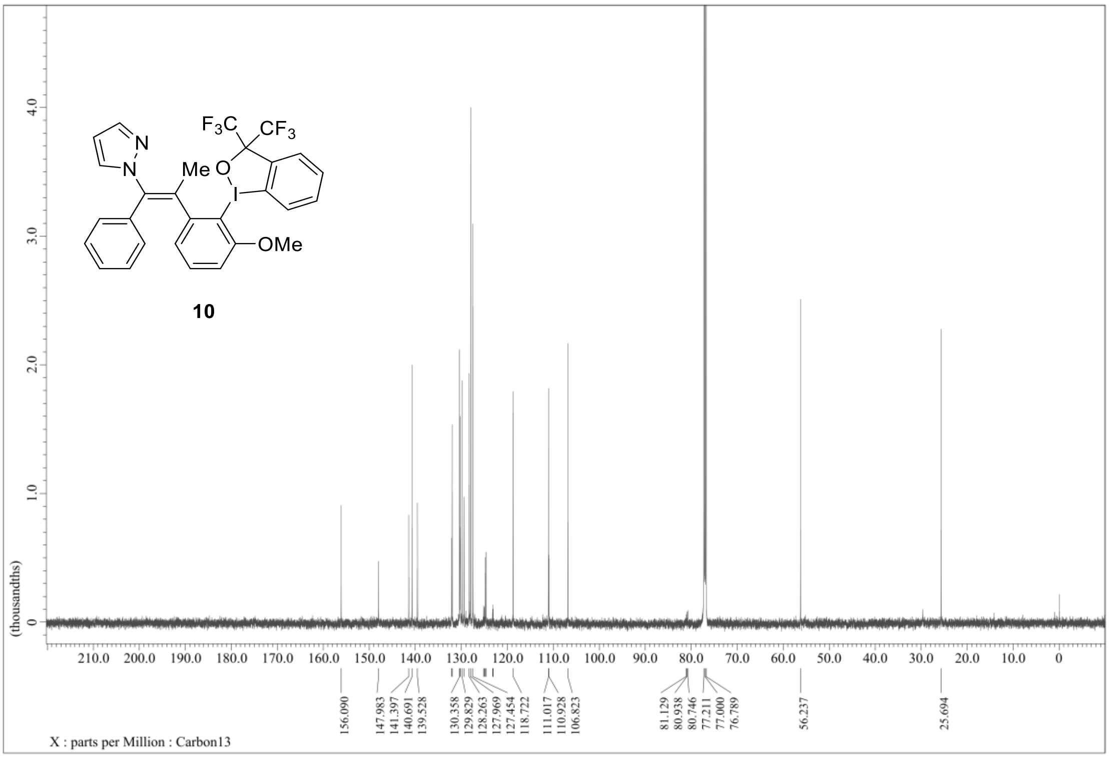

^{19}F NMR (565 MHz, $CDCl_3$) spectrum of **10**

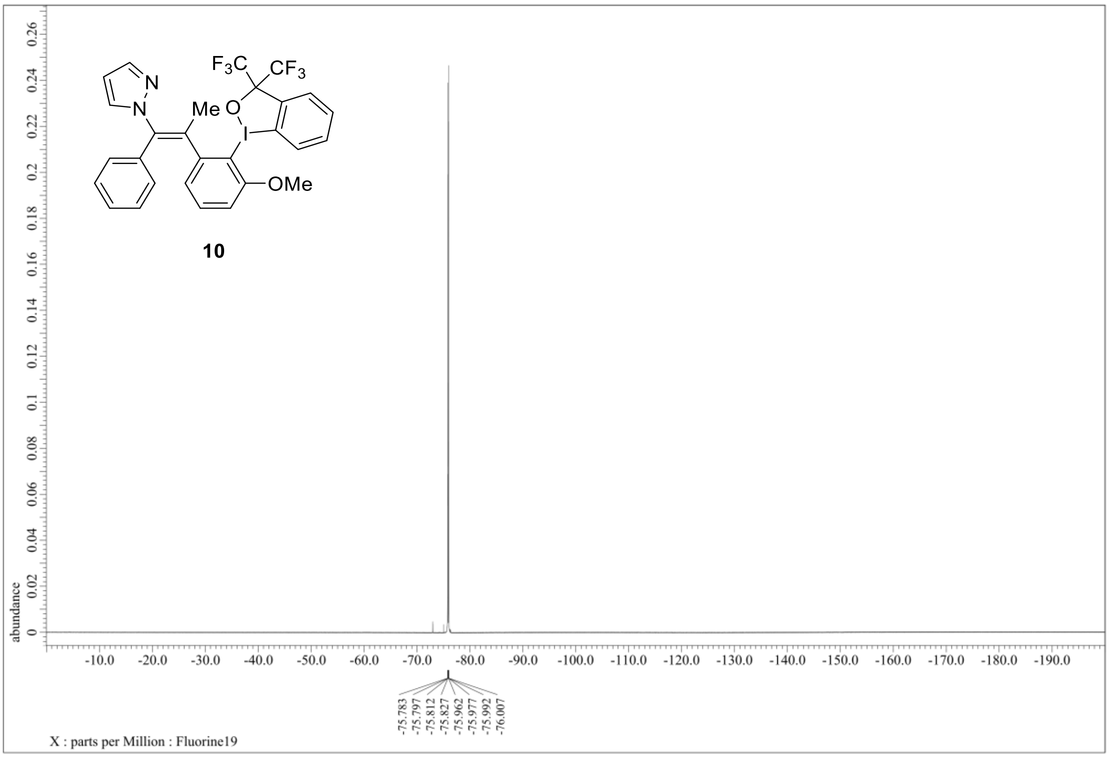

Supplement: File 1 — Experimental procedures and characterization data of new compounds. [file Beilstein_J_Org_Chem-20-891-s001.pdf]
